# FOXC2 and WT1 regulate transcriptional reprogramming during the podocyte response to injury

**DOI:** 10.1172/jci.insight.190175

**Published:** 2026-06-08

**Authors:** Sandrine Ettou, Anya Greenberg, Sangyoon Lee, Arjun Rajesh, Liang Sun, Nahid Tabibzadeh, Haruka Oishi, Ran Konoe, Phillip J. McCown, Sean Eddy, Victoria Driscoll, Tomoya Miyoshi, Ken Hiratsuka, Jason Lam, R. Sathish Srinivasan, Youngsook L. Jung, Biju Isaac, Mingwei Sun, Mary E. Taglienti, Keith Keller, Hong Chen, Matthias Kretzler, Astrid Weins, Ryuji Morizane, Shira Rockowitz, Valerie A. Schumacher, Dongwon Lee, Jordan A. Kreidberg

**Affiliations:** 1 Department of Urology, Boston Children’s Hospital, Boston, Massachusetts, USA.; 2 Department of Surgery, Harvard Medical School, Boston, Massachusetts, USA.; 3 Division of Nephrology, Department of Pediatrics, Boston Children’s Hospital, Boston, Massachusetts, USA.; 4 Division of Nephrology, Department of Medicine, Beth Israel Deaconess Medical Center, Boston, Massachusetts, USA.; 5 Research Informatics, Department of Information Technology, Boston Children’s Hospital, Boston, Massachusetts, USA.; 6 Nephrology Division, Massachusetts General Hospital, Boston, Massachusetts, USA.; 7 Department of Medicine, Harvard Medical School, Boston, Massachusetts, USA.; 8 Department of Internal Medicine, Division of Nephrology, University of Michigan, Ann Arbor, Michigan, USA.; 9 John A. Paulson School of Engineering and Applied Sciences, Harvard University, Cambridge, Massachusetts, USA.; 10 Wyss Institute for Biologically Inspired Engineering, Boston, Massachusetts, USA.; 11 Renal Division, Brigham and Women’s Hospital, Boston, Massachusetts, USA.; 12 Boston University School of Medicine, Boston, Massachusetts, USA.; 13 Cardiovascular Biology Program, Oklahoma Medical Research Foundation, Oklahoma City, Oklahoma, USA.; 14 Department of Biomedical Informatics, Harvard Medical School, Boston, Massachusetts, USA.; 15 Department of Pathology, Brigham and Women’s Hospital and Harvard Medical School, Boston, Massachusetts, USA.; 16 Broad Institute of Harvard and MIT, Cambridge, Massachusetts, USA.; 17 Program in Vascular Biology, Boston Children’s Hospital, Boston, Massachusetts, USA.; 18 Harvard Stem Cell Institute, Cambridge, Massachusetts, USA.; 19 The Manton Center for Orphan Disease Research,; 20 Children’s Rare Disease Collaborative, and; 21 Division of Genetics and Genomics, Boston Children’s Hospital, Boston, Massachusetts, USA.; 22 Department of Pediatrics, Harvard Medical School, Boston, Massachusetts, USA.

**Keywords:** Genetics, Nephrology, Molecular biology, Transcription

## Abstract

Transcriptional reprogramming has an important role in kidney glomerular disease. Using in vivo murine models of podocyte injury, we studied the roles of the FOXC2 and WT1 transcription factors (TFs) in podocyte injury. Podocytes are a crucial cell type of glomeruli, the filtration units of each nephron. Podocyte injury is often the incipient event leading to chronic kidney disease. It is well established that the TFs FOXC2 and WT1 are required in podocytes to maintain the glomerular filtration barrier. Their role in the response to injury is less well understood. Here, we tested the hypothesis that FOXC2 and WT1 act together to mediate transcriptional reprogramming in response to podocyte injury. Similarly to that of WT1, genome-wide FOXC2 binding to target genes is dynamic during the course of injury, initially increasing, but late in injury there is a dramatic decrease in FOXC2 expression and in its binding to target genes. Podocyte-specific inactivation of *FoxC2* or *Wt1* in adult mice limits the transcriptional response to injury. Correlating FOXC2 and WT1 ChIP-seq analyses demonstrated that they co-bind many genes expressed in podocytes. Thus, reprogramming the transcriptome involves dynamic changes in the binding of FOXC2 and WT1 to their target genes during a reparative injury response.

## Abstract 2

Chronic kidney disease most often results from injury to podocytes, a key cell type of the glomerulus, which is the filtering unit of each nephron. We have studied how podocytes respond to injury by regulating gene expression.

## Graphical abstract

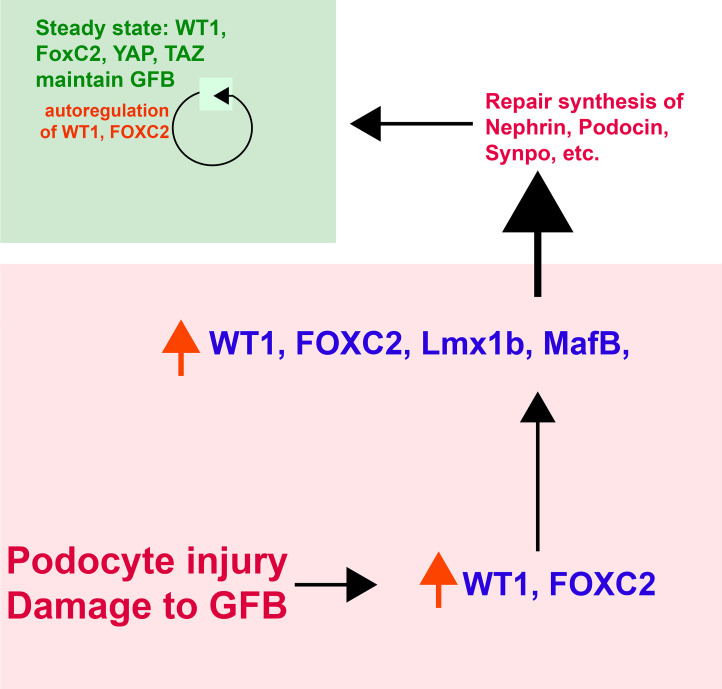

## Introduction

The glomerular filtration barrier (GFB) keeps plasma proteins such as albumin, as well as cells such as red blood cells, leukocytes, and platelets, in the circulation. Breakdown of the GFB, as occurs in several forms of severe kidney disease, results in proteinuria, i.e., the loss of albumin and other plasma proteins in the urine, and consequently edema due to decreased plasma oncotic pressure. This clinical condition is referred to as the nephrotic syndrome, which can be life-threatening and requires dialysis and/or kidney transplantation if recalcitrant to other treatments. Podocytes are terminally differentiated cells. As such, they are unable to proliferate in response to podocyte loss in order to repair a compromised GFB. Therefore, podocyte loss often results in devastating glomerular diseases such as focal segmental glomerulosclerosis (FSGS) (1, 2). However, podocytes also have a limited capacity to respond to damage and restore the GFB. We recently demonstrated that murine podocytes respond to injury through transcriptional reprogramming. During the response to adriamycin-mediated (ADR-mediated) injury, a transient increase in WT1 upregulates expression of target genes encoding podocyte proteins important to maintaining the GFB, such as *Nphs1*, *Nphs2*, and *Synpo*, encoding nephrin, podocin, and synaptopodin, respectively, among many others (3). In metazoan organisms, transcription factors (TFs) act in a combinatorial fashion, thereby allowing a limited number of TFs to confer complex patterns of lineage-specific and temporally regulated gene expression (4, 5). Less well understood is how the combinatorial action of TFs mediates transcriptional reprogramming in response to injury. Our previous study found Forkhead domain, MAFB, LMX1B, TEAD, and TCF21 motifs frequently adjacent to WT1-bound sites (3), raising the question of whether changes in their combinatorial binding play an important role in the response to injury and preservation of the GFB.

FOXC2 is a Forkhead family TF known to regulate gene expression in podocytes (6, 7). WT1 and FOXC2 are also crucial for podocyte differentiation (8, 9). Here, we examined how FOXC2 and WT1 act interdependently, mediating transcriptional reprogramming during podocyte injury. We demonstrate that FOXC2 maintains baseline gene expression in murine podocytes. Moreover, both FOXC2 and WT1 are essential for the response to injury. ChIP-seq analysis identified FOXC2 target genes in normal and injured podocytes, demonstrating the extent to which WT1 and FOXC2 share a common set of target genes. This study constitutes a first step in determining how dynamic combinatorial TF binding in podocytes affects transcriptional reprogramming during the response to injury.

## Results

### FoxC2 expression and target gene binding increase in response to injury.

**Figure 1:**
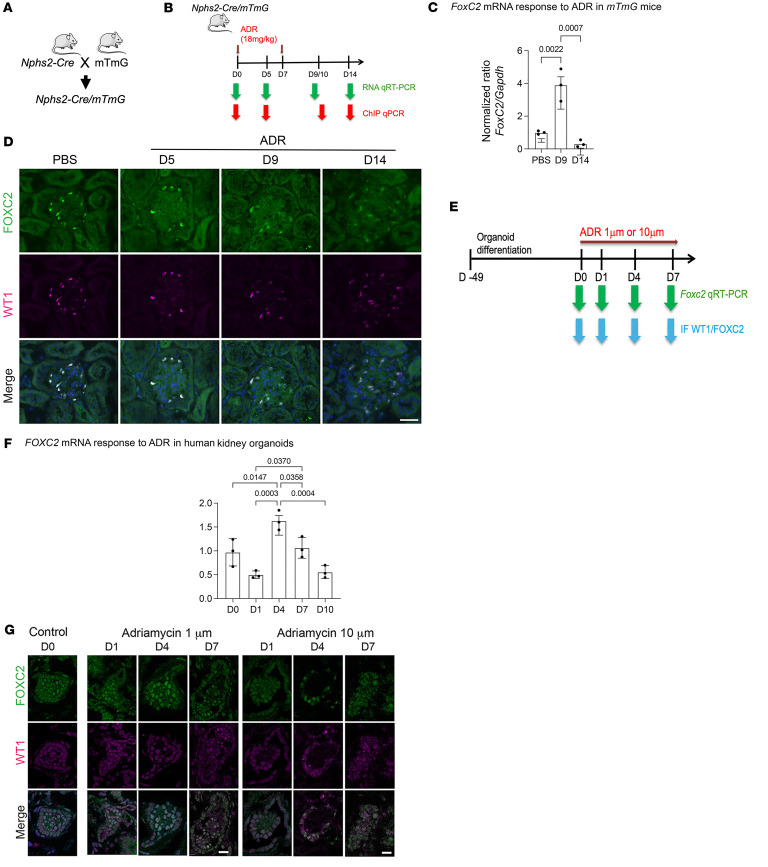
Time course of FOXC2 expression after injury in mice and human kidney organoids. (**A**) Mating scheme to obtain *Nphs2-Cre/mTmG* mice. (**B**) Experimental timeline with *Nphs2-Cre/mTmG* mice. (**C** and **D**) *FoxC2* mRNA levels detected by quantitative RT-qPCR after ADR injury (**C**) and immunofluorescent staining of FOXC2, WT1, and merged image (**D**) from *Nphs2-Cre/mTmG* mice. Each time point is representative of at least 20 glomeruli. Scale bar: 25 μm. (**E**) Experimental timeline with human kidney organoids. (**F** and **G**) *FoxC2* mRNA detected by RT-qPCR at time points after addition of 1 μM ADR (**F**) and immunofluorescent staining of FOXC2, WT1, and merged image (**G**) from human kidney organoids. Each set of panels is representative of at least 5 glomeruli in each of 3 organoids. Scale bars: 20 μm. Quantification of **D** and **G** is in Supplemental Figure 2. Data are presented as mean ± SD. One-way ANOVA with Tukey’s multiple-comparison test (**C** and **F**).

We first investigated dynamic changes in FOXC2 expression and target gene binding during the response to ADR-mediated podocyte injury, a murine model for FSGS (10–12). Our previous study demonstrated that ADR treatment causes a transient increased expression of WT1 and many of its target genes in glomeruli (3). Using the prototypical ADR-sensitive BALB/c strain (13), we found that *FoxC2* mRNA similarly increased in glomeruli after treatment with ADR (Supplemental Figure 1, A and B; supplemental material available online with this article; https://doi.org/10.1172/jci.insight.190175DS1). ADR treatment of *Nphs2-Cre/mTmG* mice (breeding and timeline in Figure 1, A and B), used to obtain purified podocytes by FACS, also produced a several-fold increase in *FoxC2* (Figure 1C). (Being less sensitive to ADR than the prototypical ADR-sensitive BALB/c strain, they require a higher dose and second treatment with ADR at day 7 [D7]). Two predicted Forkhead sites are among three WT1-bound sites near the *Nphs2* transcriptional start site (TSS), and one predicted Forkhead site is present at the most 5′ of three WT1-bound sites near the *Synpo* TSS (3). Direct ChIP-qPCR demonstrated dynamic FOXC2 binding at these sites during the course of injury, with maximal binding at several sites correlating with the peak levels of *FoxC2* mRNA (Supplemental Figure 1, C and D, and results for BALB/c in Supplemental Figure 1E). Late in injury, FOXC2 binding fell well below the baseline level observed in control mice. WT1 and FOXC2 were colocalized in podocytes (Figure 1D), and immunofluorescent (IF) staining for both was more intense in response to ADR (Figure 1D, and quantification in Supplemental Figure 2A). However, by D14 after ADR, FOXC2 protein fell well below baseline levels. Human kidney organoids were used as an additional model to study the response to injury, to add human relevance (timeline in Figure 1E) (3). As in ADR-treated mice, there was a transient increase in the *FoxC2* mRNA level (Figure 1F). Treatment of organoids with 1 μM ADR led to a transient increase of FOXC2 protein as detected by quantitative IF analysis at D4, whereas 10 μM ADR led to decreased intensity of FOXC2, greatly diminished at D7 (Figure 1G, and quantification in Supplemental Figure 2). Control organoids treated with PBS showed no change in FOXC2 over the same time course (Supplemental Figure 2B, and quantification in Supplemental Figure 2C).

### FOXC2 is required to maintain podocyte-specific gene expression and in the podocyte response to injury.

**Figure 2:**
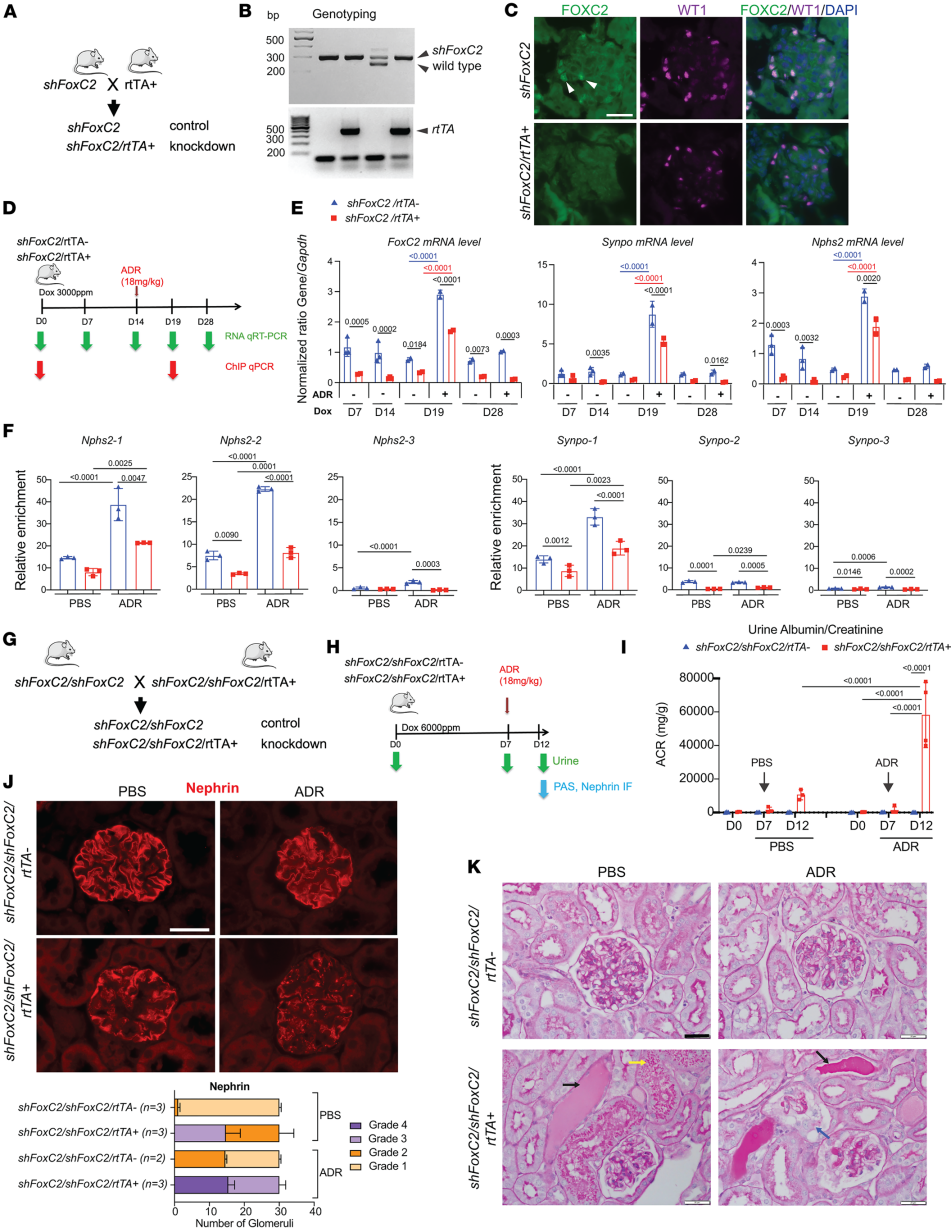
FOXC2 is required for the podocyte response to injury. An inducible *FoxC2* shRNA was expressed in podocytes of adult mice. (**A**) Breeding scheme to obtain *shFoxC2/rtTA+* mice used in **C**–**F**. *Nphs1-rtTA* mice are referred to in figures as *rtTA*. (**B**) Example of genotyping PCR. (**C**) Immunofluorescent staining of FOXC2, WT1, and merge, demonstrating loss of FOXC2 and retention of WT1 in *shFoxC2/rtTA+* mice. Arrowheads, FOXC2-positive nuclei. Scale bar: 25 μm. (**D**) Experimental timeline for **E** and **F**. (**E**) mRNA detected by RT-qPCR of *FoxC2*, *Synpo*, and *Nphs2*. The time points after beginning of doxycycline diet (3,000 ppm) and receiving of ADR or PBS control treatment are shown below the graphs. Blue, *FoxC2* shRNA without *Nphs1-rtTA* mice; red, *FoxC2* shRNA with *Nphs1-rtTA* mice. (**F**) ChIP-qPCR detection of FOXC2 binding. Sites in *Nphs2* and *Synpo* are indicated in Supplemental Figure 1C. Color designation as in **E**. (**G**) Mating scheme to obtain homozygous *shFoxC2/shfFoxC2/rtTA+* mice. (**H**) Experimental timeline for mice treated with 6,000 ppm doxycycline (**I**–**K**). (**I**) Urine albumin/creatinine ratios. (**J**) Top panel: Nephrin localization. Genotypes at left. Left, PBS treated; right, ADR treated. Scale bar: 25 μm. Bottom panel: Grading of nephrin localization patterns. (**K**) Periodic acid–Schiff–stained histological analysis. Black arrows, protein casts; yellow arrows, protein absorption granules; blue arrows, glomerular collapse. Scale bar: 20 μm. Evaluation of pathological changes is shown in Supplemental Table 1. Data are presented as mean ± SD. Two-way ANOVA or mixed-effects model (**E**), or 2-way ANOVA with Tukey’s multiple comparison test (**F** and **I**); for better visualization, not all *P* values are shown.

We tested the requirement of FOXC2 to maintain and amplify gene expression by expressing a podocyte-specific doxycycline-inducible *FoxC2* shRNA in control and ADR-treated adult mice (*FoxC2* shRNA/*Nphs1-rtTA* mice are henceforth referred to as *shFoxC2/rtTA+* mice; breeding, genotyping, and timeline in Figure 2, A, B, and D). IF staining for FOXC2 confirmed its loss in podocytes (Figure 2C). Baseline expression of *FoxC2* itself as well as *Synpo* and *Nphs2* was significantly reduced in *shFoxC2/rtTA+* mice (Figure 2E). *FoxC2* knockdown significantly diminished the typical increase in *FoxC2*, *Nphs2*, and *Synpo* mRNAs in response to ADR (Figure 2E). At the later time point after ADR, *FoxC2*, *Nphs2*, and *Synpo* levels were greatly decreased in knockdown versus control mice. The initial increase of *FoxC2* in ADR-treated knockdown mice suggests that *FoxC2* shRNA did not achieve a complete knockdown. Nevertheless, direct ChIP-qPCR demonstrated reduced FOXC2 binding at *Nphs2* and *Synpo* (Figure 2F), which was most pronounced after ADR treatment. Determining whether loss of FOXC2 resulted in proteinuria required that we use a higher dose of doxycycline and homozygosing the *Foxc2* shRNA transgene (Figure 2, G and H). *shFoxC2/shFoxC2/rtTA+* mice developed low levels of proteinuria (Figure 2I), demonstrating that the GFB is compromised in these mice. To further support this finding, the pattern of nephrin, a crucial component of the GFB, was quantified using the scale shown in Supplemental Figure 3. IF staining revealed abnormal localization of nephrin (Figure 2J). Furthermore, periodic acid–Schiff staining revealed pathological changes consistent with a compromised GFB, including protein casts and albumin reabsorption droplets (Figure 2K). Treatment of *shFoxC2/shFoxC2/rtTA+* mice with ADR resulted in high levels of proteinuria, greatly reduced localization of nephrin (Figure 2, I and J), and, by D12, decreased numbers of podocytes (Supplemental Figure 3) and histological evidence of glomerular collapse (Figure 2K). The pathological findings are summarized in Supplemental Table 1.

In addition, we tested the requirement for FOXC2 using a second model of podocyte injury. “Nephrotoxic serum” (NTS) was formulated by immunization of sheep with an extract of rat glomeruli and has been widely used to induce a transient proteinuria that is manifest 24 hours after intravascular infusion and completely resolves within the next 24–48 hours (14). Using a lower dose of NTS that induced very mild proteinuria in control *shFoxC2/shFoxC2/rtTA–* mice, we observed high levels of proteinuria in *shFoxC2/shFoxC2/rtTA+* mice 24 hours after infusion (Supplemental Figure 4, A and B). Normal sheep serum (NSS) used as a control did not elicit proteinuria. We followed these mice until D28, and observed that in contrast to control *shFoxC2/shFoxC2/rtTA–* mice, proteinuria persisted in *shFoxC2/shFoxC2/rtTA+* mice. While the level of proteinuria at D28 did not significantly differ between NSS- and NTS-treated *shFoxC2/shFoxC2/rtTA+* mice, the pathology was dramatically worse. In the former, many glomeruli showed a segmental hyalinosis; the latter exhibited many glomeruli with global hyalinosis or glomerulosclerosis (Supplemental Figure 4, B–E).

### WT1 is required for the podocyte response to injury.

WT1 is required to maintain normal podocyte gene expression (3, 15). Using adult *Wt1^fl/fl^/Nphs2-CreERT2* mice, we performed an inducible podocyte-specific knockout of *Wt1* (referred to as *Wt1* conditional knockout [*Wt1-*CKO] herein), to test the requirement for WT1 in the response to injury (breeding and genotyping in Supplemental Figure 5, A and B). Moderate levels of proteinuria developed 10–14 days after gene inactivation, providing an experimental window (Supplemental Figure 5C). ADR-treated *Wt1-*CKO mice rapidly developed proteinuria, far exceeding that of either knockout of *Wt1* or ADR alone (Supplemental Figure 5C). Additionally, in *Wt1*-CKO mice, mRNA levels of *Wt1* itself, and *Synpo*, a WT1 target gene (3, 15, 16), not only failed to show the transient increase demonstrated in our previous study, but fell far more precipitously than in control mice (Supplemental Figure 5, D and E).

### Dynamic FOXC2 binding at genes required to maintain the GFB.

**Figure 3:**
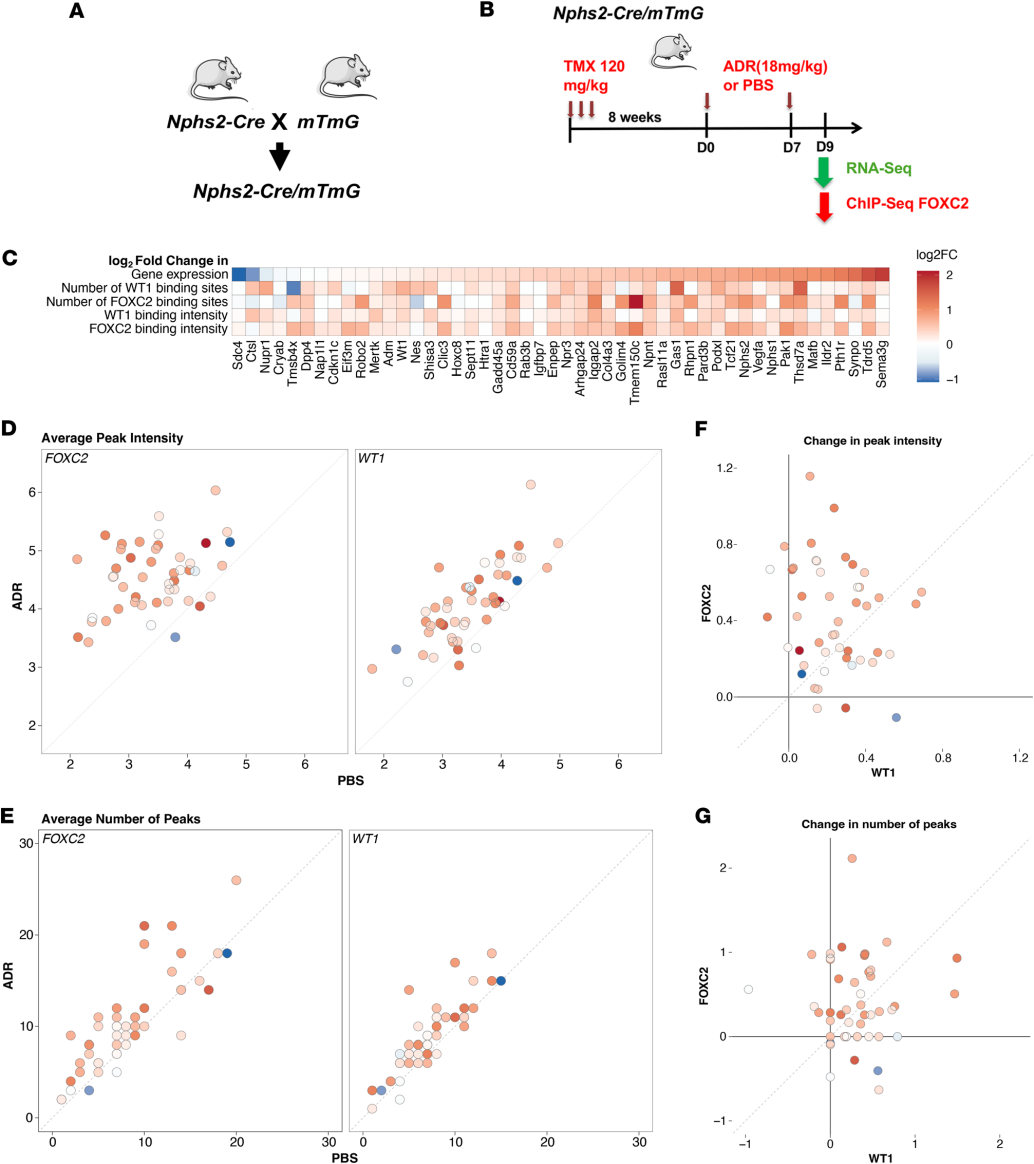
Dynamic FOXC2 and WT1 binding to a set of podocyte genes. (**A**) Breeding scheme for *Nphs2-Cre/mTmG* mice. (**B**) Experimental timeline for RNA-seq and FOXC2 ChIP-seq experiments. (**C**) Heatmap showing log_2_ fold changes (log2FC) between PBS and ADR at day 9 (D9) in gene expression, number of WT1 binding sites, number of FOXC2 binding sites, average WT1 peak intensity, and average FOXC2 peak intensity. Color bar represents log_2_ fold change in respective metrics and is scaled for all metrics in heatmap. (**D**) PBS versus ADR plot of average peak intensity for FOXC2 (left) and WT1 (right). Each dot represents one of the 48 genes characteristically expressed in podocytes, as discussed in the text and in ref. 18. For all scatterplots, dots are color-coded according to gene expression levels shown in the top row of the heatmap. (**E**) Same as **D** for number of peaks. (**F**) Log_2_ fold change in average peak intensity plotted as FOXC2 versus WT1. (**G**) Same as **F** for number of peaks.

Previous studies demonstrated that FOXC2 binds many genes in normal podocytes (17). In order to gain a more comprehensive understanding of the role of FOXC2 in glomerular disease, we produced FOXC2 ChIP-seq datasets that included a model of podocyte injury. As *FoxC2* expression is not restricted to podocytes, we performed ChIP on FACS-sorted podocytes. Adult *Nphs2-Cre/mTmG* mice were treated with ADR or PBS (control) at D0 and D7, and podocytes were sorted by FACS at D9; these are the same mouse strain and time points studied in our WT1 ChIP-seq analysis (breeding and timeline in Figure 3, A and B) (3). D9 after the initial ADR treatment was selected as the time point when FOXC2 binding was maximal at several target sites in *Nphs2* and *Synpo* (Figure 1). Importantly, despite the low levels of detection of FOXC2 binding by direct ChIP-qPCR (and in contrast to WT1 [ref. 3]), FOXC2 ChIP-seq studies were not possible at D14 after ADR treatment, as statistically significant genome-wide binding was essentially absent, suggesting that greatly decreased FOXC2 activity may be a major determinant of irreversible podocyte injury in human kidney disease.

The many studies on podocyte biology, including studies on inherited forms of glomerular disease, have identified a group of genes encoding proteins crucial for maintaining the GFB. Our previous study identified many of these as WT1 target genes (3). Furthermore, a recent study identified a set of 48 genes characteristically expressed in podocytes (18), which we also identified as WT1 target genes (3). Analysis of our RNA-seq dataset obtained from FACS-sorted podocytes demonstrated that 70% of these genes had increased expression during the response to ADR injury (34/48; Figure 3C), 9 of which had greater than 50% increase (*P* < 0.05). Compared with all WT1 and FOXC2 target genes with increased expression (excluding the 48), the subset had a significantly higher proportion of upregulated genes (48% vs. 88%; *P* = 1.88 × 10^–8^) (Supplemental Figure 6A). In addition, FOXC2 ChIP on FACS-sorted podocytes after ADR injury had increased peak intensity at 45/48 (Figure 3, C and D) and an increased number of peaks at 35/48 of these genes (Figure 3, C and E). Compared with all FOXC2 target genes (excluding the 48), the subset had significantly increased peak intensity (*P* = 3.40 × 10^–12^) and an increased number of peaks (*P* = 1.45 × 10^–3^) (Supplemental Figure 6, B and D). We analyzed our previous results with WT1 in a similar fashion. Compared with all WT1 target genes (excluding the 48), the subset showed significantly increased peak intensity (*P* = 2.00 × 10^–4^) but not a significantly increased number of peaks (*P* = 0.889) (Figure 3, C–E, and Supplemental Figure 6, B and D) (to facilitate comparisons, data from our previous study [ref. 3] are incorporated into Figure 3 and Supplemental Figure 7). Direct comparison of FOXC2 and WT1 binding demonstrated on average greater increases in FOXC2 than WT1 peak intensity (*P* = 5.75 × 10^–5^) (Figure 3F and Supplemental Figure 6C) and peak numbers upon injury (*P* = 1.89 × 10^–3^) (Figure 3G and Supplemental Figure 6E). The Integrative Genomics Viewer (IGV; https://igv.org) plots for these 48 genes, and additionally those for *FoxC2* and *Lmx1b*, are provided in Supplemental Figure 7. Sixty-five percent of peaks are bound by both FOXC2 and WT1 across these genes. (The raw data for Figure 3, C–G, are provided in Supplemental Data File 1.) These IGV plots also demonstrate increased peak intensity at one or more FOXC2 or WT1 binding sites as well as the acquisition of novel FOXC2 and/or WT1 binding sites during injury, at most of these genes. Loss of peaks or decreased peak intensity is much less frequently observed. Overall, these analyses demonstrate that FOXC2 and WT1 are integrally involved in regulating podocyte-specific gene expression and the response to injury, which may represent an attempt to repair the GFB.

### Interdependent coordinate target gene binding by FOXC2 and WT1.

**Figure 4:**
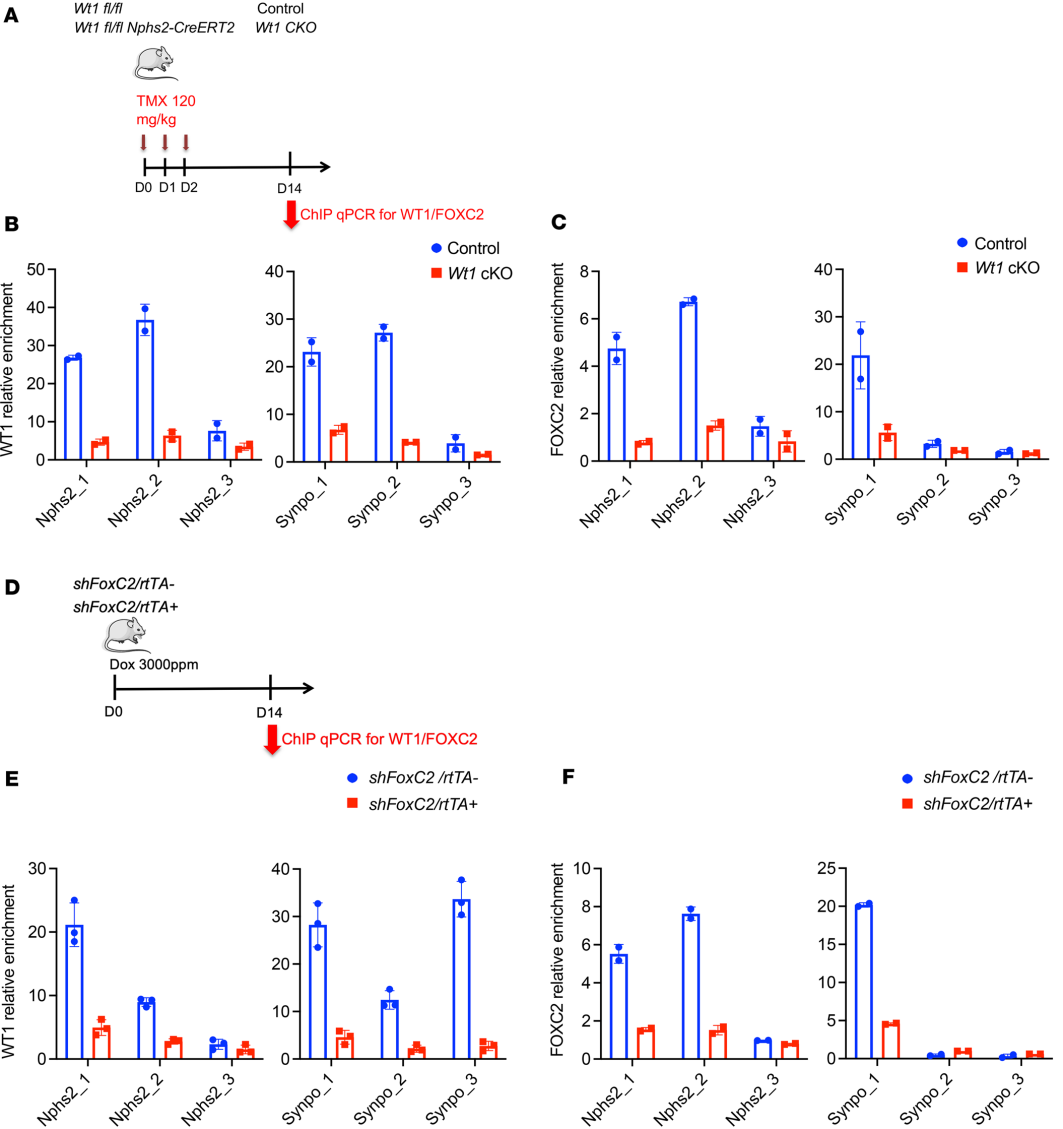
Interdependence of WT1 and FOXC2 binding. (**A**) Experimental timeline for *Wt1^fl/fl^/Nphs2-CreERT2* mice,\ referred to in the figure as *Wt1* CKO. Each pair of bars in **B**, **C**, **E**, and **F** shows binding at sites in *Nphs2* or *Synpo* designated in Supplemental Figure 1C. (**B** and **C**) Red, *Wt1^fl/fl^/Nphs2-CreERT2*; blue, control *Wt1^fl/fl^*. (**B**) WT1 ChIP-qPCR. (**C**) FOXC2 ChIP-qPCR. (**D**) Experimental timeline for *shFoxC2/Nphs1-rtTA* mice, referred to in the figure as *shFoxC2/rtTA*. (**E** and **F**) Red, shRNA *FoxC2/rtTA*; blue, *FoxC2* shRNA without *rtTA*. (**E**) WT1 ChIP-qPCR. (**F**) FOXC2 ChIP-qPCR. Data are presented as mean ± SD. Unpaired *t* test (**B**, **C**, **E**, and **F**).

Our results thus far strongly suggest that WT1 and FOXC2 coordinately regulate transcription in podocytes. Using the *Wt1*-conditional-knockout mouse (Figure 4, A–C) and the *FoxC2*-knockdown mouse (Figure 4, D–F), we demonstrated by direct ChIP-qPCR that FOXC2 and WT1 were mutually dependent on each other for binding to their target sites on *Nphs2* (Figure 4, B and E) and *Synpo* (Figure 4, C and F). Knockdown of *FoxC2* or *Wt1* in immortalized podocytes demonstrated a similar mutual dependence for binding to the *Nphs2-1* and *Synpo1-1* sites (Supplemental Figure 8, A–D). Furthermore, similarly to in vivo results, knockdown of *Wt1* or *FoxC2* in immortalized podocytes reduced expression of *Nphs2* and *Synpo* (Supplemental Figure 8, E and F).

### Genome-wide analysis of FOXC2 binding after injury.

The genome-wide analysis of FOXC2 binding serves to emphasize the dynamic nature of TF binding. Over 38,000 peaks were called as significant over input for PBS (*n* = 38,882) and ADR (*n* = 38,331) conditions, representing FOXC2-bound sites in the genome (Supplemental Figure 9A). Half were present in both conditions, 25% present only in PBS, and 25% present only in ADR. A more stringent comparison was made by directly comparing ChIP-seq signals in PBS with those in ADR (Supplemental Figure 9A, pie charts). About 75% of sites bound in both conditions did not show a significant change. In the remaining 25%, many more had increased than decreased intensity in ADR. Among peaks bound only in PBS, one-third had decreased binding or became unbound in ADR (Supplemental Figure 9A). Conversely, among peaks bound only in ADR, about two-thirds had increased binding.

Motif enrichment analysis identified WT1, FOXC2, LMX1B, TEAD3, and KLF6 among the most enriched motifs found near FOXC2 binding sites (Supplemental Figure 9B). In a more stringent analysis, looking at only sites with increased FOXC2 binding after injury, LMX1B and TEAD motifs were among the most prominent (Supplemental Figure 10A, left). LMX1B is a crucial TF for podocyte function (19, 20) previously demonstrated to act with FOXC2 to regulate *Nphs2* (21). TEAD is another important podocyte TF associated with YAP and TAZ transcriptional effectors (22–26). Gene Ontology (GO) analyses showed increased FOXC2 binding peaks in genes known to regulate kidney-related and cytoskeletal assembly pathways (Supplemental Figure 10B). This enrichment suggests that increased FOXC2 binding may be involved in regulating genes most crucial for podocytes and their response to injury. In contrast, FOXC2 binding decreased at sites near FOS/JUN motifs that are commonly found at the TSS of many genes (Supplemental Figure 10A, right) and in genes regulating pathways related to filamentous actin assembly (Supplemental Figure 10B), suggesting that these sites are less likely to regulate genes with specific roles in podocytes during injury. Most FOXC2 binding was intronic, intergenic, or TSS proximal (Supplemental Figure 10C). Most differential binding sites were intronic or intergenic (Supplemental Figure 10D), consistent with general findings that lineage-specific regulation occurs primarily at enhancers rather than promoters (5).

### Correlation of FOXC2 binding with RNA-seq.

**Figure 5:**
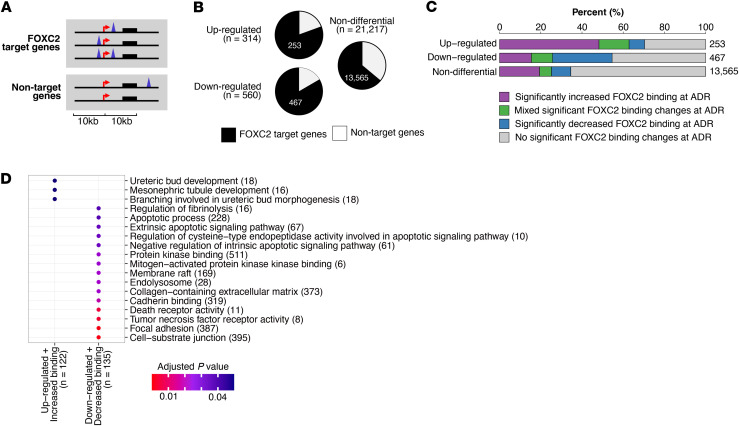
Correlation of FOXC2 binding with gene expression. (**A**) Schematic designating FOXC2 target genes as having at least one FOXC2 site within 10 kb of the TSS. (**B**) Pie charts showing proportion of up- and downregulated genes and non-differentially expressed genes that are classified as FOXC2 target genes. (**C**) Bar plots showing proportion of upregulated (top), downregulated (middle), and non-differentially expressed (bottom) FOXC2 target genes with indicated FOXC2 binding change in ADR compared with PBS condition. (**D**) GO terms enriched among genes upregulated with increased FOXC2 binding (left column) and downregulated with decreased binding (right column).

The correlation of genome-wide ChIP-seq and RNA-seq datasets demonstrates an important concept in TF biology: within a particular cell lineage, a single TF may be a component of both an activating and a repressive complex. Furthermore, it suggests an important role for FOXC2 in regulating genome-wide expression. Eighty percent of genes that were upregulated after ADR injury were found to be FOXC2 target genes (*P* = 9.9 × 10^–11^) (compared with 64% of non-differentially expressed genes), as were 83% of downregulated genes (*P* = 2.2 × 10^–24^) (Figure 5, A and B). Moreover, there was significant enrichment of differential binding of FOXC2 in response to ADR in differentially expressed genes compared with non-differentially expressed genes. Specifically, compared with 34% of non-differentially expressed genes, nearly 70% of upregulated genes (*P* = 4.3 × 10^–31^) and 50% of downregulated genes (9.4 × 10^–19^) showed significant changes in FOXC2 binding (Figure 5C). GO analysis identified developmental kidney terms enriched among genes with increased FOXC2 binding and expression, in agreement with the results presented in Supplemental Figure 10. Conversely, many pathways that might be undesirable during podocyte repair, such as those related to cell death, were downregulated and correlated with decreased FOXC2 binding (Figure 5D).

### Coordinate ChIP-seq and RNA-seq analysis of FOXC2 and WT1 target genes.

**Figure 6:**
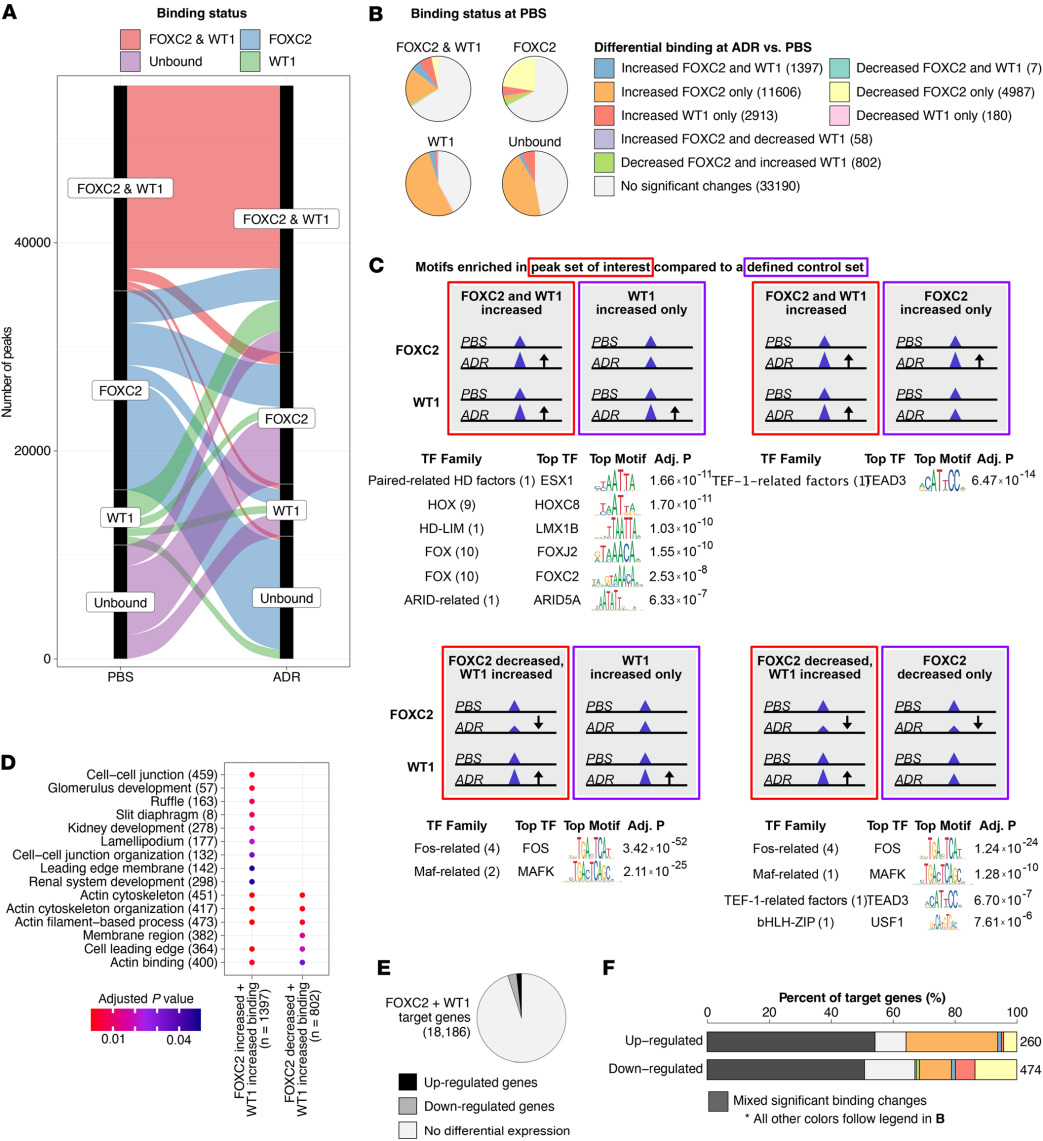
Analysis of FOXC2 and WT1 co-binding. (**A**) Alluvial plot of FOXC2 and WT1 co-binding in PBS and ADR conditions. Binding is based on statistically significant increased intensity over background. (**B**) Pie charts show proportion of sites for each binding status in PBS that have indicated peak intensity changes in ADR compared with PBS. Color key denoted by boxes to the right of charts. (**C**) Statistically significant motifs enriched when comparing regions where the intensity of co-binding changed, versus a defined control set. In each pair of gray boxes, the peak set of interest is outlined in red, and the direction of change for FOXC2 and WT1 is indicated; the control set used for comparison is outlined in purple. Below the gray boxes, TF motif families enriched with adjusted *P* value ≤ 1 × 10^–5^ in each comparison, the TF within the family with the lowest *P* value, the TF motif, and the adjusted *P* value are noted. (**D**) GO analysis based on increased binding of both FOXC2 and WT1 in ADR (left column) and decreased binding of FOXC2 and increased WT1 in ADR (right column). (**E**) Pie chart shows proportion of up- and downregulated genes that are classified as target genes co-bound by FOXC2 and WT1. The white portion includes genes that are targets of only one TF. (**F**) Bar plot shows proportion of up- and downregulated co-bound target genes with indicated binding change in ADR. The color key follows that in **B** with the addition of a mixed category (dark gray).

To examine FOXC2 and WT1 co-binding throughout the genome, we identified sites bound significantly above input, categorizing binding sites depending on whether FOXC2 and/or WT1 binding was present in the PBS and/or the ADR condition. FOXC2 bound many more sites than were previously found for WT1 (3). The largest set was bound by both TFs in both conditions, of which 11% changed binding status after injury (Figure 6A). Furthermore, significant proportions of sites bound by FOXC2 only or WT1 only in the PBS condition became co-bound in ADR (16% and 55%, respectively). Furthermore, only 20% of FOXC2-only sites remained FOXC2-only in ADR. The remainder became co-bound or completely unbound in ADR, with 8% becoming bound by WT1 only.

To further examine how co-binding changed in response to injury, we directly compared binding in the ADR condition with that in the PBS condition (Figure 6B). We found that 60% of peaks did not significantly change between PBS and ADR (gray), suggesting that much binding is stable and is not affected by injury. However, this comparison also demonstrates the dynamic nature of FOXC2 binding, showing significantly increased FOXC2 binding (orange), particularly among the sites unbound or WT1-only-bound in PBS. Moreover, about 20% of sites that bound only to FOXC2 showed decreased FOXC2 binding in ADR (yellow). Very few sites showed decreased binding of both FOXC2 and WT1 (teal), suggesting that their binding is not always mutually dependent. Sites with increased binding of both FOXC2 and WT1 (27) were less common than sites where only FOXC2 (orange) or WT1 increased (red), suggesting that they do not always drive the other’s binding. Overall, the large number of FOXC2-only sites and the dynamic nature of FOXC2 binding indicate that, while FOXC2 very often co-binds with WT1, especially at genes important for podocyte function, it also functions independently, in many cases acquiring binding after injury and, in others, losing that binding. As discussed above, these results are consistent with the ability of a single TF to participate in multiple different processes, both activating and repressive, in a single cell type.

Motif enrichment analysis identified motifs enriched at sites where binding of both TFs increased compared against controls where only WT1 or only FOXC2 binding increased. The former comparison identified LMX1B motifs as one of the most significantly enriched, whereas the latter identified TEAD motifs (Figure 6C). While the number of sites where both FOXC2 and WT1 binding increased is limited, these results are consistent with the individual analyses (Supplemental Figures 9 and 10) (3) and suggest that FOXC2 and WT1, together with LMX1B and/or TEAD, may be jointly involved in regulating genes most crucial for the podocyte response to injury. GO analysis identified glomerular development, slit diaphragm, and several pathways related to cytoskeletal assembly enriched in sites where both FOXC2 and WT1 binding increased (Figure 6D), consistent with the hypothesis that they co-bind to genes important in the podocyte response to injury. In contrast, sites where FOXC2 binding decreased and WT1 increased compared against sites where only WT1 increased or only FOXC2 decreased showed enrichment of FOS/JUN motifs (Figure 6C), consistent with previous analyses. Interestingly, FOS and JUN, both strong transcriptional activators (25), are associated with decreased binding of FOXC2, as well as increased binding of WT1, presenting complex patterns of transcriptional regulation.

We next examined how patterns of differential binding related to differential expression of target genes co-bound by WT1 and FOXC2. There were 18,186 co-bound target genes in total, only a fraction of which had differential gene expression. There were nearly twice as many genes with decreased expression (*n* = 474) as with increased expression (*n* = 260) in the ADR condition (Figure 6E). Because a single gene can contain multiple sites, we classified genes based on FOXC2 and/or WT1 peaks with differential binding, using categories in Figure 6B plus a category for mixed patterns (dark gray) (see *Differential binding categories for co-bound genes*). We found that increased FOXC2 binding only (orange) was more abundant in genes with increased expression, while decreased FOXC2 (yellow) was more abundant in genes with decreased expression (Figure 6F; raw data in Supplemental Data File 2), suggesting that co-bound genes often increased their expression as a consequence of increased FOXC2 binding. In contrast, at a small portion of co-bound genes, increased FOXC2 binding correlated with decreased expression, again suggestive of a repressive function of FOXC2-containing complexes. Finally, genes with decreased FOXC2 and increased WT1 (green) more commonly had decreased expression, suggesting that WT1 may also have a repressor function for a limited set of genes.

### FoxC2 in human kidney disease.

To better understand this process at the single-cell level in humans, we interrogated the new Omnibus of Cells and Nuclei (OCEAN) single-nucleus RNA-seq glomerular disease atlas, produced by the Nephrotic Syndrome Study Network (NEPTUNE) consortium (28). Given that FOXC2 is a TF with typically low abundance, the global detection rate in this experiment was low: while 18.9% of podocytes from living donors (LDs) were *FOXC2* positive, this proportion dropped to 5.9% in FSGS and 5.6% in minimal-change disease (MCD). However, among these *FOXC2*-positive podocytes, FOXC2 transcript levels were significantly higher in FSGS and MCD, compared with LDs (Supplemental Figure 11A). This suggests 2 distinct podocyte subpopulations in the diseased glomeruli. First, there are podocytes that have progressed to a late stage of injury, with very low or no expression of *FOXC2*. Second, there are surviving podocytes attempting to repair injury by upregulating *FOXC2* expression. Known *FOXC2* target genes (*WT1*, *SYNPO*, and *NPHS2*) followed a similar pattern within these *FOXC2*-positive cells, supporting this “repairing” hypothesis (Supplemental Figure 11A). Moreover, we observed a similar pattern of target upregulation, even when all podocytes were included in the analysis (both *FOXC2* positive and negative), indicating that many podocytes were in an active repair process (Supplemental Figure 11B). In this broader context, the aggregate *FOXC2* expression was significantly lower in FSGS and MCD, consistent with the later stages of injury. We note that *SYNPO* expression was attenuated compared with *WT1* and *NPHS2*, suggesting that regulation dynamics may differ across specific targets during injury.

Our FOXC2 ChIP-seq results identified many novel FOXC2 target genes that have received little or no examination of a potential role in podocyte injury. From this list and from the RNA-seq data, we selected a subset of genes where FOXC2 binding and their expression increased after treatment of mice with ADR. We then examined the expression of these genes in the OCEAN data. Among the 28 genes we evaluated (listed in Supplemental Data File 3), 19 were expressed in ≥5% of the podocytes, providing robust data for statistical evaluation. In diseased podocytes, 4 of these were upregulated, 12 were downregulated, and 3 showed no differential expression (Supplemental Figure 11C). The direction of gene expression change in disease (FSGS or MCD) compared with LDs was largely concordant.

**Figure 7:**
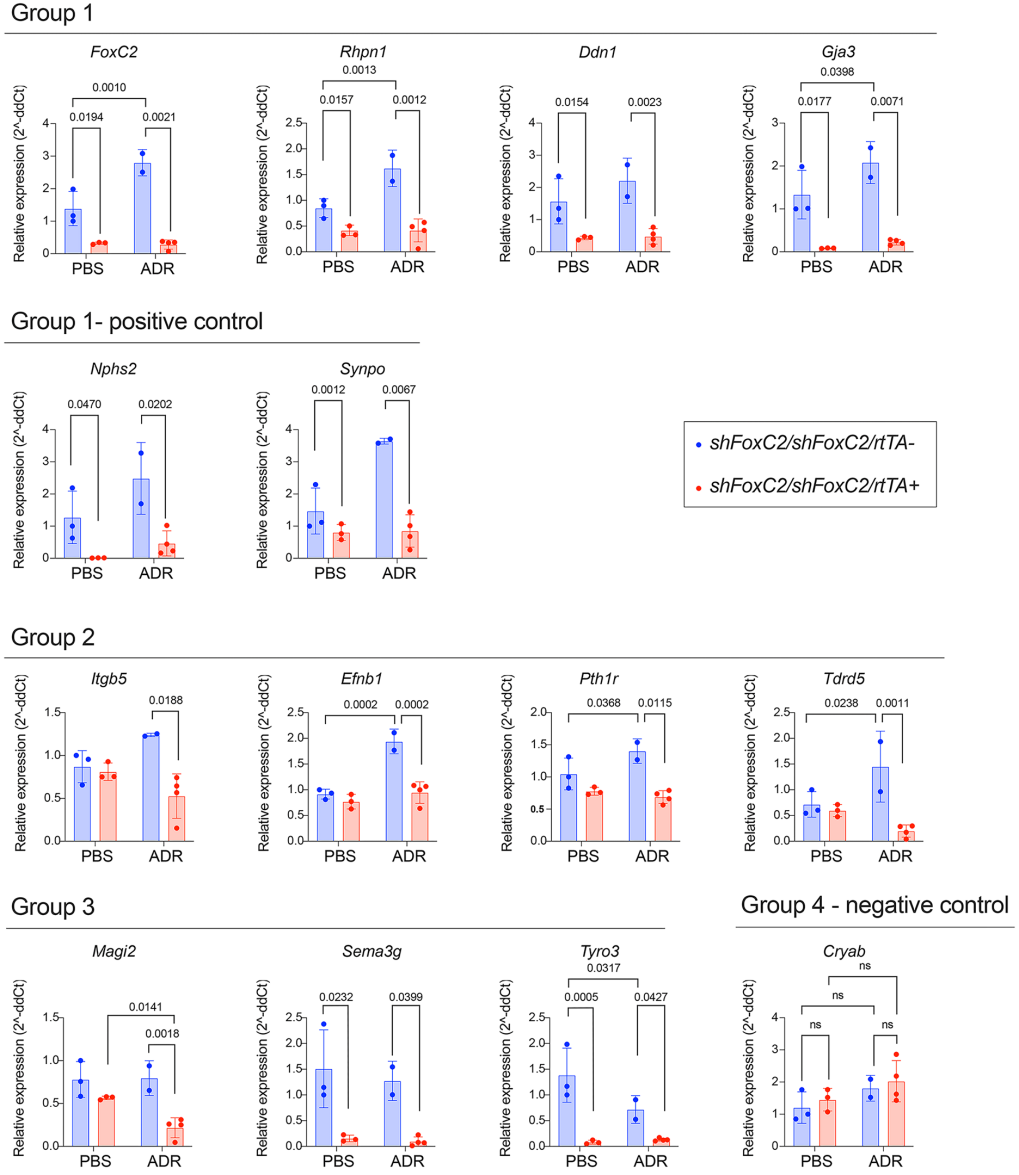
Gene expression analysis of novel FOXC2 target genes. RT-qPCR analysis of isolated glomeruli of FoxC2 and select FOXC2 target genes. Grouping is described in Results. For each target gene, *shFoxC2/shFoxC2/rtTA–* and *shFoxC2/shFoxC2/rtTA*+ mice are compared. Bars on the left show control PBS-treated mice, and bars on the right show ADR-treated mice. Data are presented as mean ± SD. Two-way ANOVA with Tukey’s multiple-comparison test.

Those genes that showed increased or decreased expression in the single-cell human data were then used for quantitative RT-PCR (RT-qPCR) analyses to directly examine their expression in isolated glomeruli from PBS- or ADR-treated *shFoxC2/shFoxC2/rtTA+* or control mice (Figure 7). Three different patterns emerged: genes that showed increased expression after ADR treatment of control mice, and dramatically decreased expression in both PBS- and ADR-treated knockdown mice (group 1); genes with increased expression after ADR but for which the effect of *FoxC2* knockdown was only observed in ADR-treated mice (group 2); and genes with unchanged or decreased expression after ADR treatment of control mice, and decreased expression in knockdown mice (group 3). The known FOXC2 target genes *Nphs2* and *Synpo* were included as positive controls and fell into group 1. Group 4 was a negative control, *Cryab*, for which neither its expression nor FOXC2 binding increased. As noted previously, podocyte depletion was evident in ADR-treated *shFoxC2/shFoxC2/rtTA+* mice (Supplemental Figure 3) but not to an extent that would by itself account for the greatly reduced expression observed for most target genes.

**Figure 8:**
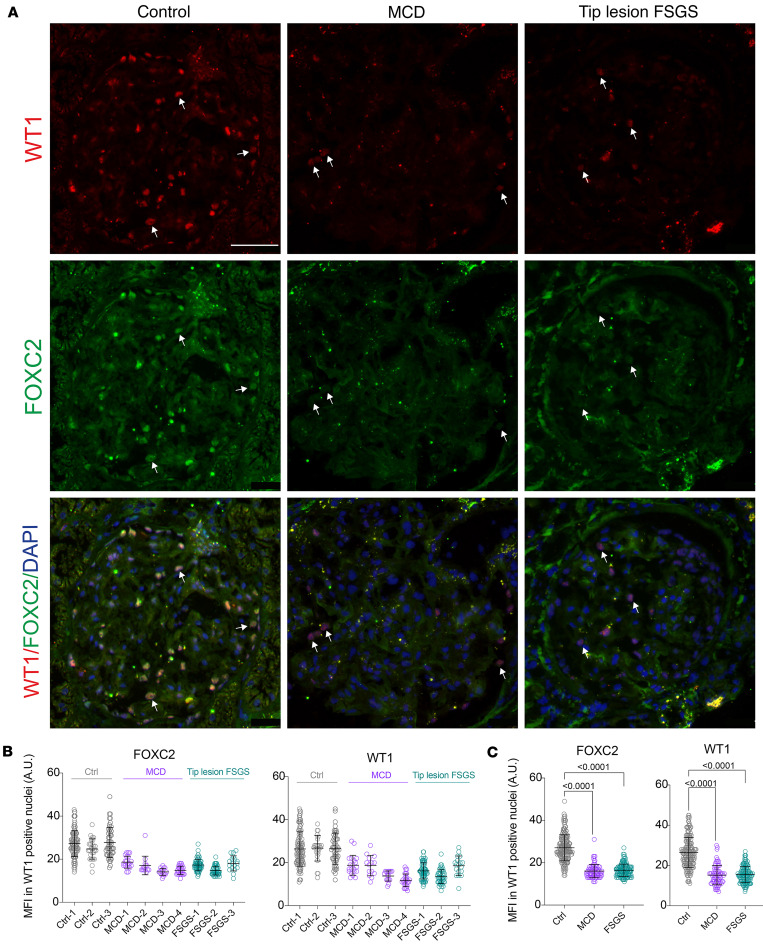
FOXC2 and WT1 in glomeruli of human kidneys. (**A**) The protein stained is given on the left of each row, and the disease condition is given at the top of each column. Each image is representative of at least 3 biopsies examined from distinct individuals and at least 4 glomeruli imaged per biopsy. White arrows indicate nuclei positive for WT1 and their location in the FOXC2 panels. Scale bar: 50 μm. (**B**) Quantification of staining of nuclei from individual biopsies; each data point is one nucleus. (**C**) Staining intensities from all individuals combined for statistical analysis, presented as mean ± SD. Kruskal-Wallis test with Dunn’s multiple-comparison test.

Colocalization of FOXC2 and WT1 was examined at the protein level in human kidney biopsies. Decreased staining intensity of both FOXC2 and WT1 was observed in pretreatment biopsies of individuals with MCD and tip lesion FSGS (Figure 8A). The criteria used for the pathological diagnoses are shown in Supplemental Table 2. The intensity of staining among several biopsies was decreased from controls but not significantly different between MCD and tip lesion FSGS (Figure 8, B and C). These observations may suggest that by the time these individuals come to clinical attention, WT1 and FOXC2 are largely unable to maintain expression of genes encoding components of the GFB. The low levels of FOXC2 and WT1 protein in human MCD and FSGS biopsies are in distinction to the results demonstrating higher levels of their respective mRNAs in a subset of podocytes. This may be due to differences in the timing of the biopsies in relation to the disease process, or may suggest that post-transcriptional processes have a role in human glomerular disease.

## Discussion

**Figure 9:**
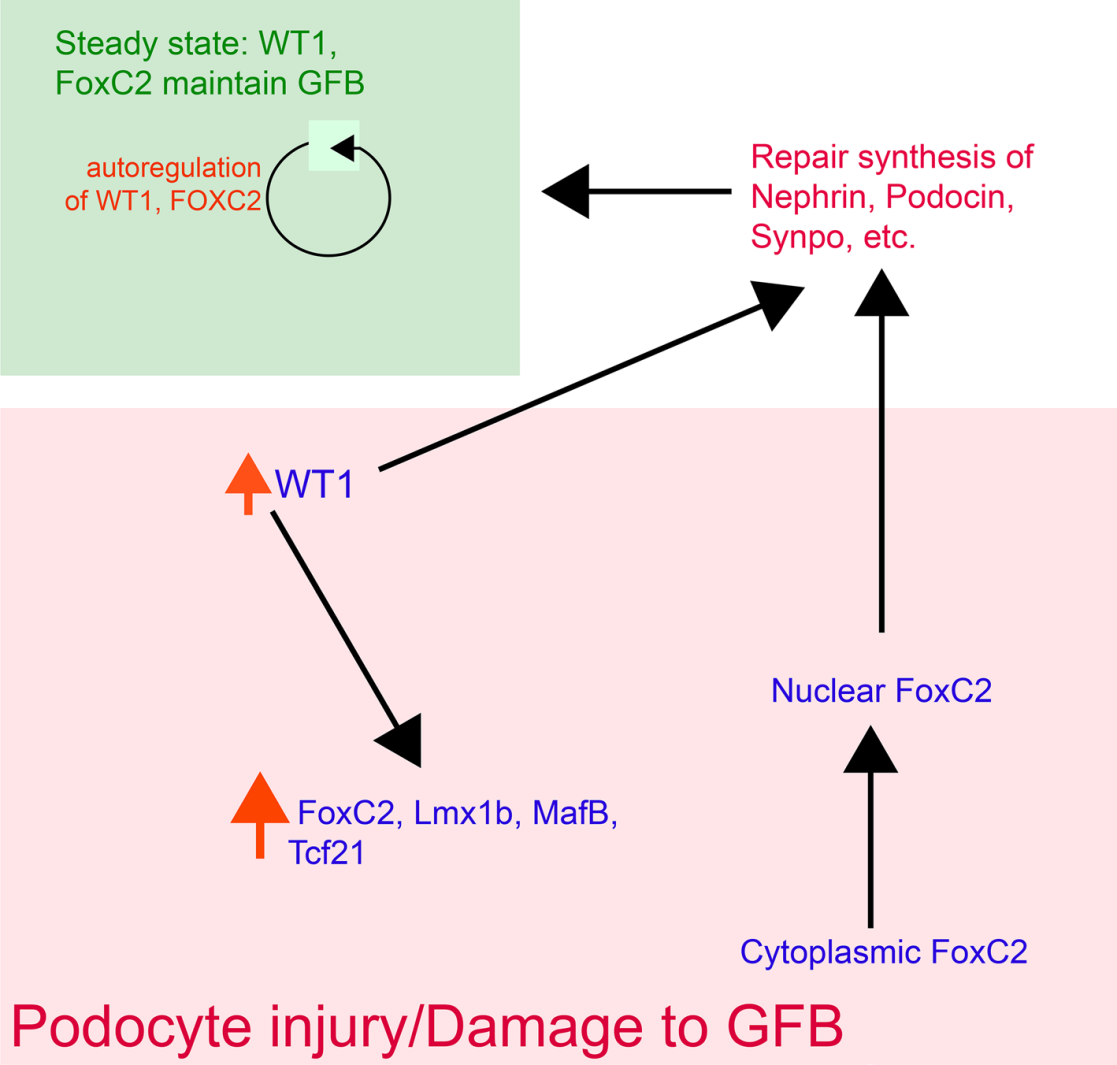
A model of the regulation of gene expression by WT1 and FOXC2 in podocytes. At steady state, autoregulation maintains expression of *FoxC2*, *Wt1*, and their target genes. Upon injury, increased expression of *Wt1* boosts expression of *FoxC2*, thereby boosting expression of other podocyte TFs, thereby increasing expression of genes encoding components of the GFB.

WT1 and FOXC2 are known to co-bind the majority of genes in podocytes in non-injury condition (17). Here we demonstrated that, after injury, binding of both WT1 and FOXC2 increases at many genes crucial for maintaining the GFB. Moreover, at late time points in a model of irreversible podocyte injury, the binding of FOXC2 is barely detectable. These observations demonstrate that transcriptional reprogramming, regulated by FOXC2 and WT1, plays a crucial role in determining the course and outcome of podocyte injury. Binding of FOXC2 and WT1 increased after injury at the great majority of the set of 48 genes characteristically expressed in podocytes and led to enhanced expression of nearly all of these genes. This leads us to hypothesize that genes crucial for maintaining the GFB may represent a “privileged” set that has evolved such that FOXC2 and WT1, and presumably other TFs prominently expressed in podocytes, act coordinately to boost their expression during the podocyte response to injury. A limitation of this study is that a single model of podocyte injury, that of ADR toxicity to podocytes, was used to generate the ChIP-seq and RNA-seq datasets. A model summarizing our results is presented in Figure 9.

Our study identified many novel FOXC2 target genes expressed in podocytes. Those that were validated by direct examination (Figure 7) emphasize some interesting mechanisms important in podocyte function. The importance of rhophilin-1 (*Rhpn1*), a GTPase-interacting protein, has previously been demonstrated (29). TYRO3 is a TAM receptor highly expressed in podocytes that may have a protective effect through the activation of AKT and suppression of NF-κB (reviewed in ref. 30). MAGI2, a WW and PDZ domain–containing membrane-associated guanylate kinase belonging to the MAGUK family, is known to be important in podocyte structure (29). In contrast to these FOXC2 targets that are podocyte protective, dendrin (*Ddn1*) is a large protein of uncertain function that localizes to the nucleus after podocyte injury (31). Loss of dendrin attenuates podocyte injury; thus it is paradoxical that its expression appears to be stimulated by FOXC2. On the other hand, *Ddn1* is also a FOXC2 target in non-injury conditions, suggesting that in non-injury conditions, dendrin may help preserve normal structure.

In contrast to the observations with genes encoding proteins important for the GFB, our genome-wide results suggest that FOXC2 and WT1 may also be components of repressive complexes, emphasizing the important concept that TFs may be involved in both gene activation and repression, even in the same cell type. This is most likely due to the context of their binding at any particular site, where distinct combinations of other TFs may be bound. For example, FOXC2 and/or WT1 might be involved in repression at sites not co-bound by LMX1B or MAFB. The presence of different protein isoforms or posttranslational modifications of FOXC2 or WT1 may provide an alternate mechanism determining activator versus repressor functions. This is plausible for WT1, which is known to have several isoforms based on alternative splicing and multiple translational start sites. In contrast, FOXC2 has a single exon and is not known to have multiple isoforms. However, FOXC2 has multiple well-described phosphorylation sites (32), which offer possibilities for distinct posttranslational modifications.

Our previous study and the present one identify several TFs known to be crucial for podocyte function, including LMX1B, MAFB, KLF6, and others (20, 21, 33–36), as WT1 and FOXC2 target genes, suggesting that WT1 and FOXC2 may be among the most upstream TFs in podocytes. Furthermore, the observation that FOXC2 and WT1 bind their own and each other’s genes leads us to suggest that TF autoregulation maintains baseline expression and increases expression of FOXC2 and WT1 in response to injury (Figure 9). Indeed, autoregulation has been suggested for WT1 (37). Loss of FOXC2 and WT1 might eventually lead to insufficient expression of crucial target genes, including themselves. There are likely additional processes during podocyte injury that are also responsible for the great decrease in FOXC2 binding to its target genes. We speculate that the great decrease in FOXC2 binding to its target genes (something not observed to the same degree with WT1) may be an important determinant of irreversible podocyte injury and may be relevant to the progression of human kidney diseases such as FSGS. The low abundance of FOXC2 RNA in podocytes found in the OCEAN dataset likely contributes to an underestimation of FOXC2-positive podocytes. This may explain why most podocytes were continuing to express *NPHS2* and *SYNPO*, although many of the other FOXC2 target genes examined in Supplemental Figure 11 were expressed in fewer podocytes and at low levels or were not expressed. Nevertheless, the low percentage of podocytes expressing *FOXC2* found in the OCEAN data is consistent with its low levels at the later time point after ADR injury in mice. It would be interesting for future studies to determine whether FOXC2 is more highly expressed and in a greater proportion of podocytes in treated individuals recovering from MCD.

## Methods

### Sex as a biological variable

All studies were done using male mice, as they are more susceptible than female mice to the glomerular injury models used in this report.

### Cell culture

Immortalized mouse podocytes (38) were cultured with RPMI 1640 medium (Corning), 10% fetal bovine serum, 5% sodium pyruvate solution (100 mM; Thermo Fisher Scientific). Undifferentiated cells were cultured at 33°C in the presence of murine interferon-γ (IFN-γ; 10 U/mL) (R&D Systems). To induce podocyte differentiation, cells were shifted to 37°C for 14 days in the absence of IFN-γ.

### Mice

Animal studies were approved by the Institutional Animal Care and Use Committees at Boston Children’s Hospital and Beth Israel Deaconess Medical Center. BALB/cJ mice were from The Jackson Laboratory. *R26-mTmG* mice were from The Jackson Laboratory (stock 007676). All experiments in this report involved using mice of mixed genetic background and comparing littermate mice. ADR (Cayman Chemical) or PBS was administered to BALB/cJ (Charles River Laboratories) and *Nphs2-Cre/mTmG* mice by retro-orbital injection (10.5 and 18 mg/kg, respectively) under isoflurane anesthesia. *Nphs2-Cre/mTmG* mice received 2 injections at a 1-week interval. *Nphs2-CreERT2/WT1^fl/fl^*/*R26R-tdTomato* mice (WT1 CKO) were obtained using *WT1^fl/+^* mice (39) with *Nphs2-CreERT2* (40) and *R26R-tdTomato* mice (The Jackson Laboratory 007909). *Nphs2-CreERT2 WT1^fl/fl^ R26R-tdTomato* mice were given tamoxifen (120 mg/kg) during 3 consecutive days by intraperitoneal injections. *shFoxC2* mice were derived according to published procedures (41, 42) and crossed with *Nphs1-rtTA* [Tg(Nphs1-rtTA*3G)8Jhm] mice (43) (obtained from Jeff Miner, Washington University School of Medicine, St. Louis, Missouri, USA). *Nphs1-rtTA/shFoxC2* mice received a modified rodent diet containing 3,000 ppm doxycycline (ScottPharma), and designated mice received ADR (18 mg/kg) or NTS/NSS (30 mg/kg; obtained from David Salant, Boston University School of Medicine, Boston, Massachusetts, USA). For studies of proteinuria, *Nphs1-rtTA shFoxC2* received 6,000 ppm doxycycline. Glomerular isolation has been described previously (3).

### Kidney organoid generation and ADR treatment

H9 hESCs were differentiated into kidney organoids, as reported previously (44). Organoids were treated with ADR or PBS as described previously (3) and harvested after 1, 4, 7, and 10 days of ADR injury (on days 50, 53, 56, and 59 of differentiation). All control samples were treated with PBS at an equivalent vehicle dilution ratio to that used for the 10 μM ADR condition. Human organoid and stem cell experiments were approved by the Mass General Brigham Institutional Biosafety Committee.

### Human biopsy specimens

Deidentified frozen tissue sections were obtained from the Department of Pathology at the Brigham and Women’s Hospital, under IRB protocol BWH-2011P002692. These were stained as described in “Immunofluorescence” (below).

### Immunofluorescence

Frozen sections were fixed for 10 minutes in 4% paraformaldehyde, washed with PBS, and incubated in 30% sucrose for 5 minutes, followed by permeabilization with 0.1% Triton X-100 for 5 minutes. Blocking solution was applied for 1 hour at room temperature (5% donkey serum with 2% BSA in TBS). Sections were incubated with primary antibody (anti-FoxC2, R&D Systems AF6989, 1:50; anti-WT1, Santa Cruz Biotechnology sc-192, 1:100; anti-nephrin, Progen GP-N2, 1:200) diluted in blocking solution at 4°C overnight. Secondary antibodies were incubated at room temperature for 1 hour and sections counterstained with 4′,6-diamidino-2-phenylindole (DAPI). After final washes with PBS, slides were mounted using Prolong Gold (Invitrogen). Sections were imaged using a Nikon Eclipse Ni Widefield microscope fitted with a DS-QilMc camera. Images were evaluated in ImageJ (NIH). The freehand selection tool was used to manually outline WT1-positive nuclei and to measure the mean FoxC2 fluorescence intensity of these nuclei. Organoids were fixed by 4% paraformaldehyde (45) for 1 hour at room temperature, incubated in 30% sucrose overnight at 4°C, and embedded in optimal cutting temperature (46) compound, and frozen sections (10 μm) were cut by cryostat. Frozen section samples were blocked with blocking buffer (0.3% Triton X-100 and 5% normal donkey serum) for 1 hour at room temperature and then incubated with primary antibodies (anti-WT1, Santa Cruz Biotechnology sc-192; anti-FOXC2, R&D Systems AF6989) in antibody dilution buffer (ADB; 0.3% Triton X-100 and 1% BSA in PBS) overnight at 4°C. After the organoids were washed 3 times with PBS, they were incubated with secondary antibodies and SYTOX Blue (Thermo Fisher Scientific) in ADB for 1 hour at room temperature. The organoids were washed 3 times with PBS, mounted with VECTASHIELD (Vector Laboratories), and then sealed by cover glass. Immunofluorescent imaging was performed using a Leica Stellaris 8 confocal microscope. FOXC2 fluorescence quantification was semi-automatically measured using QuPath software (47). Briefly, a cell detection method was trained from manual annotation of all the podocyte clusters (detected by WT1 positivity) to detect all the nuclei. Mean fluorescence intensity of FOXC2 was then exported from each “nucleus” object. An average of 182 ± 88.2 nuclei were analyzed per condition.

### RNA extraction, complementary DNA, and RT-qPCR analysis

Total RNA from immortalized mouse podocytes, isolated podocytes, and organoids was obtained as described previously (3). All RT-qPCR data were normalized to *Gapdh* using the ΔΔCt method. Primer sequences are listed in Supplemental Table 3.

### ChIP-qPCR and ChIP-seq

Chromatin for ChIP-qPCR and ChIP-seq was obtained as described previously (3). Fold enrichment of ChIP versus immunoglobulin G (IgG) control was calculated as 2((Ct(IgG) – Ct(input)) – (Ct(ChIP) – Ct(input))). Primer sequences are listed in Supplemental Table 3.

### Library preparation ChIP-seq

ChIP-seq libraries were prepared using NEBNext DNA library preparation reagents (New England Biolabs E6040) and the protocol and reagent concentrations described in the Illumina Multiplex ChIP-seq DNA Sample Prep Kit. Libraries were indexed using a single indexed PCR primer. Libraries were quantified by Qubit (Invitrogen) and sequenced using a HiSeq 2000 (Illumina) to generate 50 bp single-end reads.

### Informatics methods

#### RNA-seq analysis.

We reanalyzed the previously generated RNA-seq data collected at day 9 (D9) after treatment with ADR or PBS using our established pipelines (3). Specifically, we used Trimmomatic v0.39 (48)to trim the next-generation sequencing reads (-threads 20 ILLUMINACLIP:TruSeq3-PE.fa:2:30:10 LEADING:3 TRAILING:3 SLIDINGWINDOW:4:20 MINLEN:30). High-quality trimmed reads were aligned to mouse reference genome (GRCm38) using STAR 2.7.2b (49). The read counts were calculated by featureCounts software (50). After the initial quality assessment, we excluded the third replicates from the following differential expression analysis owing to their suboptimal quality. We used DESeq2 (51) to identify differentially expressed genes between ADR and PBS conditions. A nominal *P* value of 0.05 (not adjusted by multiple testing) and fold change of 50% were used to identify differentially expressed genes. To evaluate significance of the proportion of up- and downregulated genes, a binomial test was used with the probability of success (i.e., increased measure).

#### ChIP-seq data processing.

We uniformly processed newly generated FOXC2 ChIP-seq along with WT1 ChIP-seq data from the previous study (3) using our established pipelines. For each replicate, ChIP-seq raw reads were trimmed and aligned to mouse reference genome (GRCm38). Narrow and consensus peaks were called and annotated using the Nextflow nf-core/chipseq pipeline (52). We then pooled all replicates for each condition and TF to maximally identify regions bound by these TFs. These peaks were subsequently merged to generate a final list of TF-bound regions (either WT1 or FOXC2 in PBS and ADR conditions) for downstream analyses.

#### Association between FOXC2 peaks and genes.

To infer potential target genes of FOXC2 peaks, we determined whether a binding site was in a distal region, defined as 10,000 bp upstream and downstream from the TSS. If so, then we considered that gene (or genes) to be a target gene of the FOXC2 binding site. To infer potential target genes of co-bound peaks, we took the intersection of FOXC2 target genes and WT1 target genes.

### Gene-based analysis of peak intensity and peak number

For the 48 podocyte genes, we identified regions within 10,000 bp upstream and downstream of the TSS. We calculated the number of peaks per gene as the total number of peaks in that gene region. Additionally, we calculated average peak intensity as the average of all peaks within a gene region. Peak intensity was calculated as the log_2_ normalized read counts for each condition and TF, separately. To evaluate the significance of the number of genes with increased expression, a binomial test was used with the probability of success (i.e., increased measure) defined as increased number of binding sites or increased peak intensity. To evaluate the significance of differences in peak intensity and number of peaks between ADR and PBS conditions and between FOXC2 and WT1, Wilcoxon’s rank-sum test was used.

#### Differential TF binding analysis.

Differentially bound sites between ADR and PBS groups within WT1 and FOXC2 were identified with the R/Bioconductor package DiffBind using the DESeq2-based differential analysis tests (53). We compared the PBS and ADR conditions for FOXC2 and WT1, separately. Regions bound by both FOXC2 and WT1 in the same condition were considered co-bound. Regions with significant changes in binding (FDR ≤ 0.05 and fold change ≥ 1.4 or ≤ –1.4) of both FOXC2 and WT1 in the ADR or PBS condition were considered differentially co-bound.

#### TF motif enrichment analysis.

As described in ref. 3, TF motif enrichment analysis was performed for the significantly increased FOXC2 bindings in the ADR compared with the PBS condition (FDR ≤ 0.05 and fold change ≥ 1.4) using Multiple Expression motifs for Motif Elicitation (MEME) and Analysis of Motif Enrichment (AME) with default parameters (54). The same number of control sequences, as primary sequences, were chosen from FOXC2 binding sites where FOXC2 binding did not change significantly in ADR (FDR ≥ 0.05 and absolute fold change ≤ 1.4). TF motif sequences were obtained from the JASPAR CORE 2022 database (55). Among enriched TFs, those that are lowly expressed or not expressed in podocytes were removed (average transcripts per million ≤ 1 in both PBS and ADR). The same procedure was followed for significantly decreased FOXC2 bindings in ADR. Similarly, for regions with significantly increased FOXC2 bindings and significantly increased WT1 bindings, DNA sequences were obtained with a 200 bp window around the midpoint between the summits of the FOXC2 and WT1 bindings. The same number of co-bound binding sites was chosen for 2 different control sets. Sequences for one control set were chosen from regions where there was significantly increased FOXC2 binding and WT1 binding did not change significantly in ADR. Sequences for the other control set were chosen from regions where FOXC2 binding did not change significantly and there was significantly increased WT1 binding. The enrichment of TF motifs associated with increased FOXC2 and WT1 co-bindings compared with each of these control sets was carried out separately. A similar procedure was followed for regions with significantly decreased FOXC2 and significantly increased WT1 bindings. All significant motifs are provided in Supplemental Data File 4.

For assessment of motifs enriched near FOXC2-bound sites in the PBS condition, bound sites were compared with sites that were unbound in PBS. Similarly, for FOXC2-bound sites in the ADR condition, unbound sites in ADR were used as the control set. The same filters and methods listed above were used to assess results.

#### Differential binding categories for co-bound genes.

Because this is a gene-based analysis and a single gene can contain multiple bound regions, we classified genes into 2 groups. The first group consisted of genes with only one FOXC2- and/or WT1-bound region. The second group consisted of genes with multiple bound regions. For genes in the first group, we categorized them based on the binding pattern of their single peak, following these categories: (a) increased FOXC2 and WT1, (b) increased FOXC2 only, (c) increased WT1 only, (d) decreased FOXC2 and WT1, (e) decreased FOXC2 only, (f) decreased WT1 only, (g) increased FOXC2 and decreased WT1, (h) decreased FOXC2 and increased WT1, and (i) no significant changes. For genes in the second group, we further divided them into 3 subclasses based on the binding patterns represented by their associated peaks. The first subclass consisted of genes for which all peaks had no significant FOXC2 or WT1 binding changes (gray). The second subclass consisted of genes for which at least 1 peak had a significant binding change, and all peaks that did had the same differential binding pattern. These genes were categorized in a similar manner to the genes in the first group. The third subclass consisted of genes for which multiple peaks had significant binding changes and at least 2 peaks did not have the same differential binding pattern. These genes were categorized as mixed (dark gray), an additional category added to Figure 6F for this analysis.

#### GO analysis.

For regions with significantly changed FOXC2 binding and significantly changed co-binding of FOXC2 and WT1, GREAT version 4 (56) was used to determine GO terms associated with these sites. We used the basal plus extension, which defined a proximal region of 5,000 bp upstream and 1,000 bp downstream and a distal region of up to 50,000 bp, to associate genomic regions with genes. For the genes with differential expression changes and association with significant FOXC2 binding changes, Enrichr (57–59) was used. Data for Figure 5D, Figure 6D, and Supplemental Figure 10B are provided in Supplemental Data File 5.

#### NEPTUNE OCEAN single-nucleus RNA-seq analysis.

We used the OCEAN single-nucleus RNA-seq (snRNA-seq) dataset generated by the NEPTUNE project (28). Starting from the original published data as a Seurat object, we extracted cells that met the following criteria: (a) cells generated by the 10x Genomics snRNA-seq platform; (b) cells from 3 main cohorts: LDs, FSGS, and MCD; and (c) cells classified as podocytes. To ensure data robustness, we further filtered out cells with ≤1,000 unique molecular identifiers (UMIs). This process yielded a total of 4,059 high-quality podocytes for analysis, distributed as follows: LDs, *n* = 306; FSGS, *n* = 1,586; and MCD, *n* = 2,167. The default normalized gene expression values (LogNormalize by Seurat) were used for comparison. For differential expression test, a 2-sided Wilcoxon’s rank-sum test was used to compare LDs and each disease group (FSGS or MCD). The direction of expression change was determined based on the mean difference between the disease group and the LD reference.

### Statistics

Two-tailed paired Student’s *t* test was used to determine statistical significance between PBS and ADR conditions. Bars represent means and error bars ± SEMs. *P* values are included in the figures. Analysis of variance (ANOVA) with Tukey’s multiple-comparison test was used to compare different time points for WT1 ChIP-qPCR and FOXC2 ChIP-qPCR. Multiple 2-tailed *t* tests with FDR determined using the 2-stage linear step-up procedure of Benjamini, Krieger, and Yekutieli were used to compare different conditions (PBS/ADR and control/WT1 CKO). *P* values for analyses were assigned using a 1-sided paired Wilcoxon’s test.

### Data availability

The data reported here are available in the NCBI’s Gene Expression Omnibus (GEO) under accession GSE213174. A table containing all peak data, FOXC2 and WT1 binding properties under different conditions (PBS vs. ADR), and the differentially expressed gene status of their target genes is provided as Supplemental Data File 2.

## Author contributions

S Ettou conducted in vivo and in vitro experiments and analysis of results. AG conducted the informatics analyses and analysis of results and wrote the manuscript. SL conducted in vivo experiments. LS conducted informatics analyses. RK, NT, HO, TM, and KH conducted the organoid experiments. VD, AR, and JL contributed to the in vivo experiments. RSS and HC derived and contributed transgenic mice. BI, MS, and YLJ contributed to the informatics analyses. KK prepared human biopsy material. MET managed transgenic mice and contributed to the in vivo experiments. AW performed pathological analyses. RM supervised organoid experiments. SR supervised informatics analyses. PJM, S Eddy, MK, and DL generated data and performed analyses within the NEPTUNE OCEAN snRNA-seq glomerular disease atlas. VAS and JAK supervised in vivo and in vitro experiments, analyzed results, and wrote the manuscript. DL supervised the informatics analysis and wrote the manuscript. The order of co–first authors was determined by S Ettou originating the project, though contributions of S Ettou and AG were equal.

## Conflict of interest

MK reports grants and contracts through the University of Michigan from the Chan Zuckerberg Initiative, AstraZeneca PLC, Novo Nordisk, Eli Lilly and Co., Boehringer Ingelheim, European Union Innovative Medicine Initiative, Certa Therapeutics, RenalytixAI, Regeneron, Sanofi, Dimerix, Travere Therapeutics, and Vera Therapeutics. He has received consulting fees through the University of Michigan from Novo Nordisk, Alexion, Novartis, Roche Diagnostics, and Vera Therapeutics. MK has a licensed patent, PCT/EP2014/073413, “Biomarkers and methods for progression prediction for chronic kidney disease.” MK has served on the NIH-NCATS council, is the committee chair for the American Society of Nephrology Program, and is on the board of NephCure Kidney International. S Eddy reports grants and contracts through the University of Michigan from AstraZeneca PLC, Boehringer Ingelheim, Eli Lilly and Co., Certa Therapeutics, Novo Nordisk, Sanofi, Dimerix, Travere Therapeutics, and Vera Therapeutics.

## Funding support

This work is the result of NIH funding, in whole or in part, and is subject to the NIH Public Access Policy. Through acceptance of this federal funding, the NIH has been given a right to make the work publicly available in PubMed Central.

- National Institutes of Health (NIH) R01DK109972 to JAK.
- Department of Defense PR211099 to JAK.
- NIH R01HL131652 and NIH R01HL163095 to RSS.
- NIH R01HG012871 to DL.
- NIH DP2EB029388/DK133821 and NIH R01DK141567 to RM.
- DL is supported by a Manton Center Endowed Scholar Award.
- The Nephrotic Syndrome Study Network (NEPTUNE) is alumni of the Rare Diseases Clinical Research Network (RDCRN), which is funded by the NIH and led by the National Center for Advancing Translational Sciences (NCATS) through its Division of Rare Diseases Research Innovation. NEPTUNE has been funded under grant U54DK083912 as a collaboration between NCATS and the National Institute of Diabetes and Digestive and Kidney Diseases.
- Additional funding and/or programmatic support was provided by the University of Michigan, NephCure Kidney International, the Alport Syndrome Foundation, and the Halpin Foundation.
- RDCRN active consortia and alumni are supported by the RDCRN Data Management and Coordinating Center, funded by NCATS and the National Institute of Neurological Disorders and Stroke under U2CTR002818.
- Applied Systems Biology Core at the University of Michigan George M. O’Brien Kidney Translational Core Center (2P30-DK-08194).

## Supplementary Material

Supplemental data

Supplemental data set 1

Supplemental data set 2

Supplemental data set 3

Supplemental data set 4

Supplemental data set 5

Unedited blot and gel images

Supporting data values

## Version 1 06/08/2026

Electronic publication
