# Supplementary material for: FOXC2 and WT1 regulate transcriptional reprogramming during the podocyte response to injury
Source: JCI Insight. 2026 Jun 8;11(11):e190175. doi: 10.1172/jci.insight.190175 (PMC13313500; doi:10.1172/jci.insight.190175)
Supplement: Supplemental data [file jciinsight-11-190175-s311.pdf]

# Supplemental tables 1-3

Table 1. Pathological evaluation of histology of *Foxc2* knockdown mice

| Genotype-treatment | | Total gloms | Global sclerosis | Segmental hyalinosis | Glomerular collapse | Collapse + PEC reaction | dilated tubules with protein casts | Int Fibrosis (%) |
|---|---|---|---|---|---|---|---|---|
| | | | | | | | | |
| *shFoxc2/rtTA-* | PBS | 163 | 0 | 0 | 0 | 0 | 0 | 0 |
| *shFoxc2/rtTA-* | PBS | 155 | 0 | 0 | 0 | 0 | 0 | 0 |
| *shFoxc2/rtTA-* | PBS | 160 | 0 | 0 | 0 | 0 | 0 | 0 |
| *shFoxc2/rtTA* - | ADR | 159 | 0 | 0 | 0 | 0 | 0 | 0 |
| *shFoxc2/rtTA* - | ADR | 131 | 0 | 0 | 0 | 0 | 0 | 0 |
| *shFoxc2/rtTA* + | PBS | 125 | 0 | 0 | 0 | 0 | rare (trace) | 0 |
| *shFoxc2/rtTA* + | PBS | 134 | 0 | 0 | 0 | 0 | 0 | 0 |
| *shFoxc2/rtTA* + | PBS | 142 | 0 | 0 | 0 | 0 | 1+ | 0 |
| *shFoxc2/rtTA* + | ADR | 88 | 0 | 16 | 0 | 0 | 2+ | 0 |
| *shFoxc2/rtTA* + | ADR | 100 | 0 | 0 | 0 | 0 | 1+ | 0 |
| *shFoxc2/rtTA* + | ADR | 110 | 0 | 7 | 0 | 0 | 1+ | 0 |
| *shFoxc2/rtTA* + | ADR | 108 | 0 | 4 | 2 | 0 | 2+ | 0 |

PAS-stained sections were evaluated blindly by a pathologist (AW).

Table 2. Human biopsy information

| ***Case #*** | ***Age*** | ***Gender*** | ***Bx Indication*** | ***Proteinuria [UPCR or dipstick]*** | ***Renal Biopsy Diagnosis*** | ***Podocyte Effacement [%]*** | ***GS [%]*** | ***SS [%]*** | ***IFTA [%]*** |
|---|---|---|---|---|---|---|---|---|---|
| **Ctrl-1** | 25 | M | mild persistent proteinuria | 0.3g/gCr | **Unremarkable Parenchyma** | 0 | 0 | 0 | 0 |
| **Ctrl-2** | 67 | F | AKI | 0.7g/gCr | **Acute tubular injury** | 0 | <5 | <5 | 10 |
| **Ctrl-3** | 70 | F | AKI | trace | **Acute tubular injury** | 5 | 7 | 0 | 5-10 |
| **MCD-1** | 73 | F | NS | 10g/gCr | **Diffuse Podocytopathy/MCD, anti-nephrin +** | 100 | 10 | 0 | <5 |
| **MCD-2** | 94 | M | NS | 3+ | **Diffuse Podocytopathy/MCD, anti-nephrin +** | 100 | 62 | 0 | 50 |
| **MCD-3** | 66 | M | NS | 12g/gCr | **Diffuse Podocytopathy/MCD, anti-nephrin +** | 100 | 14 | 0 | 5-10 |
| **MCD-4** | 53 | M | NS | 6.2g/gCr | **Diffuse Podocytopathy/MCD, anti-nephrin +** | 100 | 8 | 0 | 5-10 |
| **TLFSGS-1** | 78 | M | NS | 15g/gCr | **Diffuse Podocytopathy/tip lesion variant, anti-nephrin +** | 100 | 23 | 5 | 20 |
| **TLFSGS-2** | 41 | M | NS | 4+ | **Diffuse Podocytopathy/tip lesion variant, anti-nephrin +** | 100 | 18 | 4 | <5 |
| **TLFSGS-3** | 63 | M | NS | 13g/gCr | **Diffuse Podocytopathy/tip lesion variant, anti-nephrin -** | 100 | 12 | 15 | 15 |

## Table 3: Primer sequences for RT-qPCRs and ChIP qPCRs

| Gene | forward primer (5'-3') | reverse primer (5'-3') |
|---|---|---|
| **RT-qPCR primers** | | |
| *Cryab* | GTTCTTCGGAGAGCACCTGTT | GAGAGTCCGGTGTCAATCCAG |
| *Ddn* | GACCCTGGGGACTAAGCGA | ACATCCCGGTAGATTCGAGGA |
| *Efnb1* | TGTGGCTATGGTCGTGCTG | CCAAGCCCTTCCCACTTAGG |
| *FoxC2* | AACCCAACAGCAAACTTTCCC | GCGTAGCTCGATAGGGCAG |
| *Gja3* | CACAGGAGCACTCTACAGTCA | CGGTCGTAGCAGACGTTCTC |
| *Itgb5* | GCTGCTGTCTGCAAGGAGAA | AAGCAAGGCAAGCGATGGA |
| *Magi2* | AGAAAAGTGGTGCTCTCCTAGA | CACGCCGTTTCCATTGACTAC |
| *Nphs2* | GCATCAAGCCCTCTGGATTAG | AGACGGAGATCAACCTTGTGATA |
| *Pth1r* | CAGGCGCAATGTGACAAGC | TTTCCCGGTGCCTTCTCTTTC |
| *Rhpn1* | GCGAGGATGACTTCTTCGAGG | CATCGGTTCTTGGTTGAAAACAC |
| *Sema3g* | AGGTGGGGAGCTATACACAGG | ACCGGGGTTCATGTAGGAGG |
| *Synpo* | CTGCATCCGTGGTCAACAG | GGGACTCCTATCCGCCATAC |
| *Tdrd5* | GGAGCCATAAGGTCCGAAACT | AGAACGGGAGACAATGCCAAA |
| *Tyro3* | TTACTGGTGGAGAGGACTCAC | CTTGAAGGCGAACAATGGCTG |
| **ChIP qPCR primers** | | |
| *Nphs2_peak1* | CACCTGGTCTCTTCACAGCA | GTCCGCAGTGACCTGGTATT |
| *Nphs2_peak2* | TCACTTCCCTGGACTCTGCT | GGAGGAGGAACTCACTGTCG |
| *Nphs2_peak3* | CATCCAGACCCAAGAAGGAA | CCCTGCTGTCCATTCTCAAT |
| *Synpo_peak1* | ATGCTTTCCTCCTGCTCAGA | GGGAAGGAGAAGGTTCCAAG |
| *Synpo_peak2* | TCCACTCTGCCATCTGTGTC | GTGCCGTCACTCTCATTCCT |
| *Synpo_peak3* | CCTGCCTTGAGTCCTTTCTG | CTGTTAGGGCAGAGCAGACC |

## Supplemental Figure 1

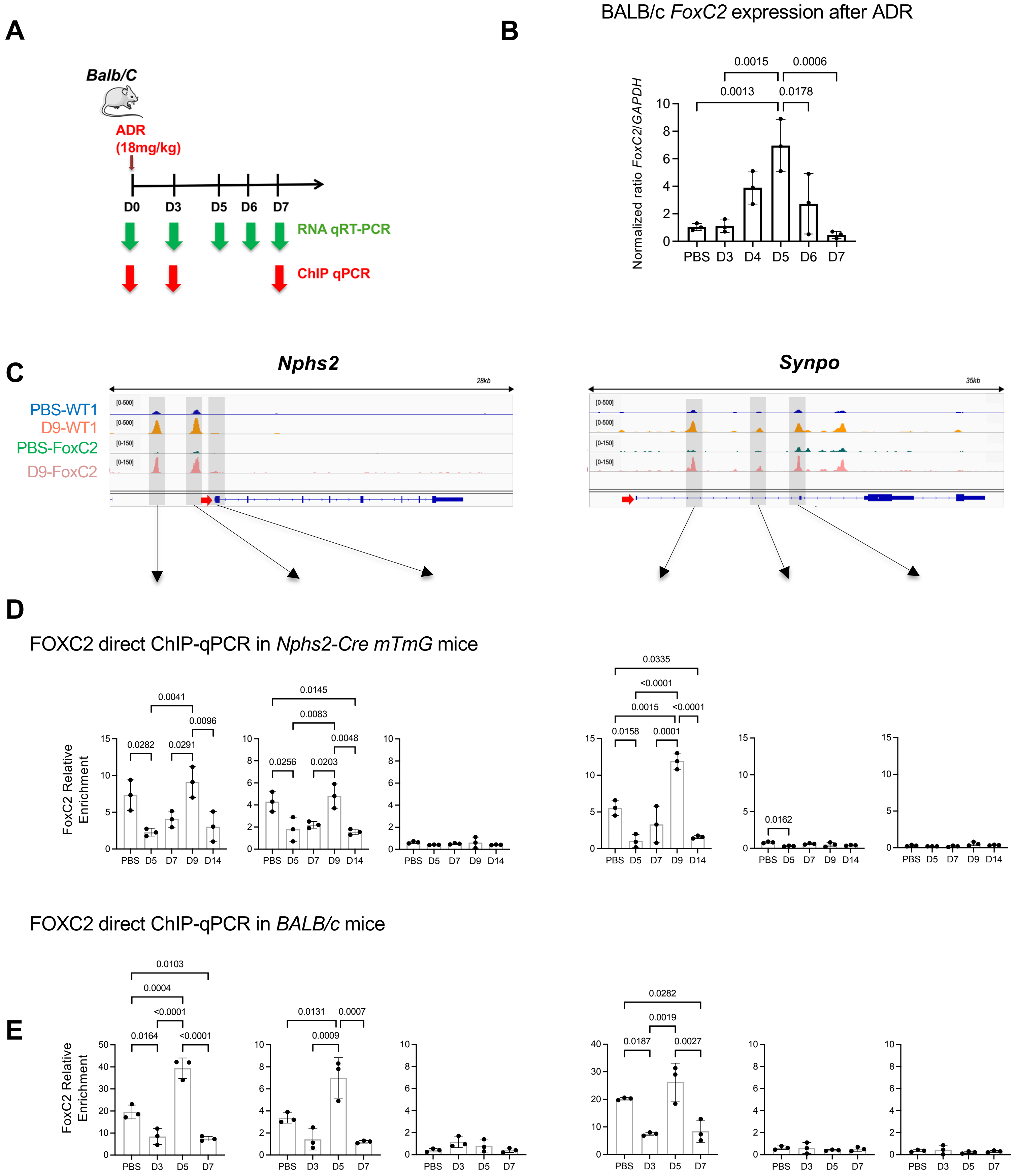

**Figure S1. FOXC2 response to ADR.** (A) Experimental timeline for Balb/c mice (B,E). Timeline for *Nphs2-Cre/mTmG* mice (D) in Figure 1. (B) *FoxC2* mRNA levels detected by RT-qPCR of isolated glomeruli from Balb/C mice after ADR injury. (C) IGV plots of FOXC2 and WT1 binding to *Nphs2* and *Synpo* after PBS or ADR at D9. (FOXC2 data is from ChIP-Seq reported herein, WT1 data is from the ChIP-seq experiment analyzed in (*3*)). *Synpo* is positioned in reverse orientation to typical IGV displays for clarity, 5' to 3' from left to right. (D) FOXC2 direct CHIP-qPCR to binding sites designated in (C), using isolated podocytes from *Nphs2-Cre/mTmG* mice. Time points at bottom of each graph. (E) same as in (D) except using chromatin from isolated glomeruli of BALB/c mice. Data are presented as mean ± SD. One-way ANOVA with Tukey's multiple comparisons test used for (B, D, E).

Figure S2

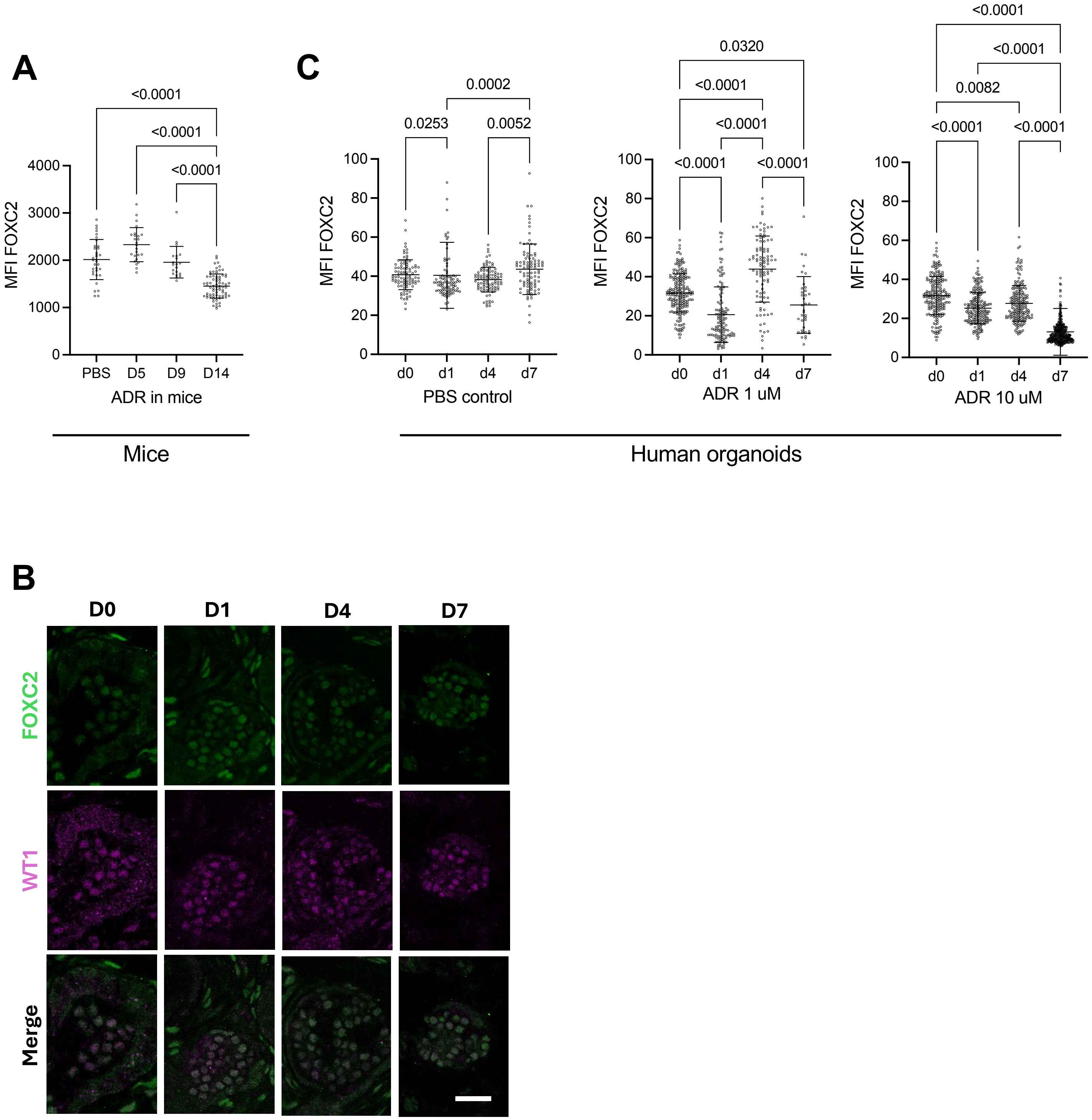
A
<0.0001
<0.0001
<0.0001
MFI FOXC2
4000
3000
2000
1000
0
PBS
D5
D9
D14
ADR in mice
Mice
C
0.0002
0.0253
0.0052
MFI FOXC2
100
80
60
40
20
0
d0
d1
d4
d7
PBS control
0.0320
<0.0001
<0.0001
<0.0001
<0.0001
ADR 1 uM
<0.0001
<0.0001
0.0082
<0.0001
<0.0001
ADR 10 uM
Human organoids
B
D0
D1
D4
D7
FOXC2
WT1
Merge

**Figure S2. Quantification of immunofluorescent staining.** (A) Quantification of the mean fluorescent intensity of FOXC2 in mice. A representative image is shown in Figure 1D. Data are presented as mean ± SD. Kruskal–Wallis test with Dunn's multiple comparisons. (B) Immunofluorescent staining of FOXC2 and WT1 in human organoids treated with PBS. Scale bar: 20μm. (C) Same as (A) for organoids shown in Figure 1 (PBS, 1μM AD, 10μM ADR). Data are presented as mean ± SD. Kruskal–Wallis test with Dunn's multiple comparisons.

Figure S3

# Nephrin Grading

**A**

**Grade 1**
- Nephrin is normal appearing with linear pattern

**Grade 2**
- Nephrin is lower intensity
- Mild vesicular appearance

**Grade 3**
- Predominantly vesicular appearing
- Breaks and disruption within linear pattern

**Grade 4**
- Severe loss of nephrin (>50% glomerular area)

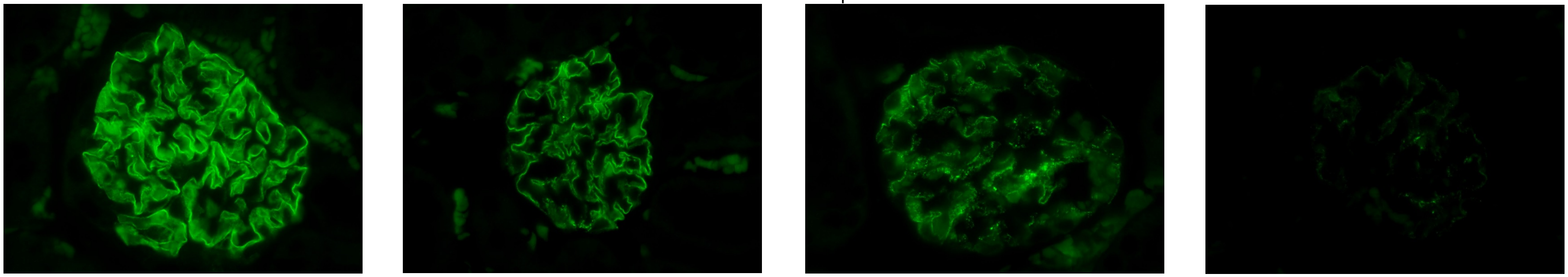

**B**

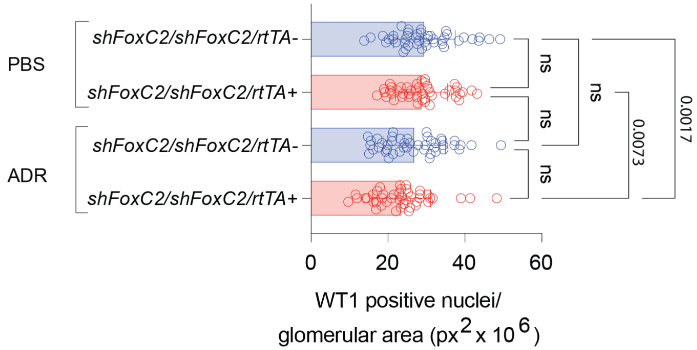

**Figure S3. Grading of nephrin localization and podocyte counts.** (A) Immunofluorescent stained sections were evaluated for nephrin localization, based on criteria shown in the figure. A blinded observer categorized 30 glomeruli per sample. (B). Quantification of WT1+ cells per glomerular area. Data are presented as mean ± SD. Two-way ANOVA with Tukey's multiple comparisons test. N=3 mice, 15 glomeruli counted per mouse.

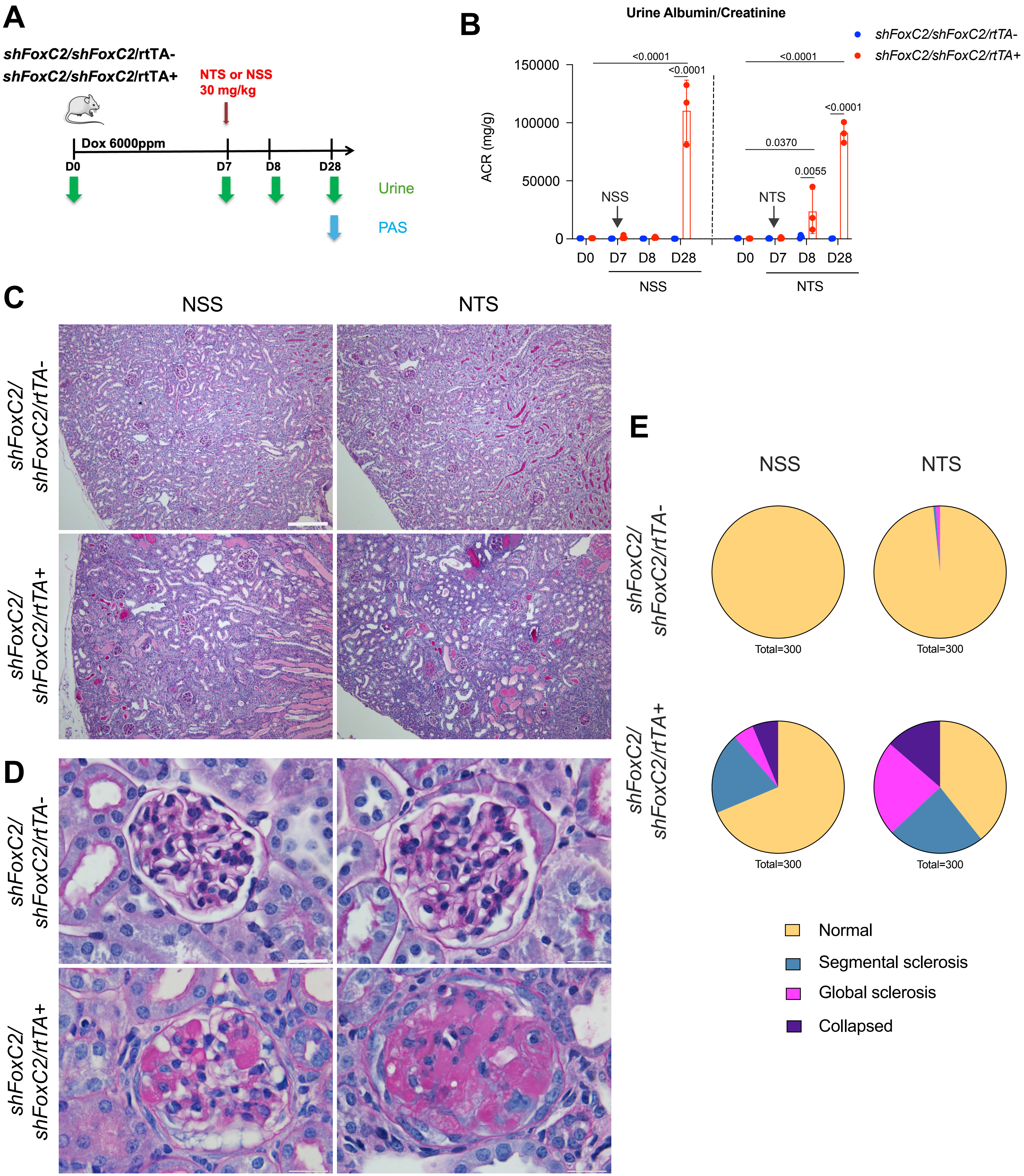
A
shFoxC2/shFoxC2/rtTA-
shFoxC2/shFoxC2/rtTA+
NTS or NSS
30 mg/kg
Dox 6000ppm
D0
D7
D8
D28
Urine
PAS
B
Urine Albumin/Creatinine
shFoxC2/shFoxC2/rtTA-
shFoxC2/shFoxC2/rtTA+
ACR (mg/g)
150000
100000
50000
0
<0.0001
<0.0001
<0.0001
0.0370
0.0055
<0.0001
NSS
NTS
D0 D7 D8 D28
D0 D7 D8 D28
NSS
NTS
C
NSS
NTS
shFoxC2/shFoxC2/rtTA-
shFoxC2/shFoxC2/rtTA+
D
shFoxC2/shFoxC2/rtTA-
shFoxC2/shFoxC2/rtTA+
E
NSS
NTS
shFoxC2/shFoxC2/rtTA-
shFoxC2/shFoxC2/rtTA+
Total=300
Total=300
Total=300
Total=300
Normal
Segmental sclerosis
Global sclerosis
Collapsed

**Figure S4 Treatment of FoxC2 knockdown mice with NTS.** (A) Experimental scheme. (B) Albumin to creatinine ratios, blue: shFoxC2/shFoxC2/rTTA-; Red: shFoxC2/shFoxC2/rTTA+ mice. D0: mice placed on doxycycline chow; The time point of NSS or NTS treatment is indicated. Data are presented as mean ± SD. Two-way ANOVA with Tukey's multiple comparisons test. (C) PAS stained representative sections low power magnification. Scale Bar 200 μm. (D) PAS stained representative glomeruli high power magnification. Scale bar: 20 μm. (E) Quantifcation of histological analysis.

Figure S5

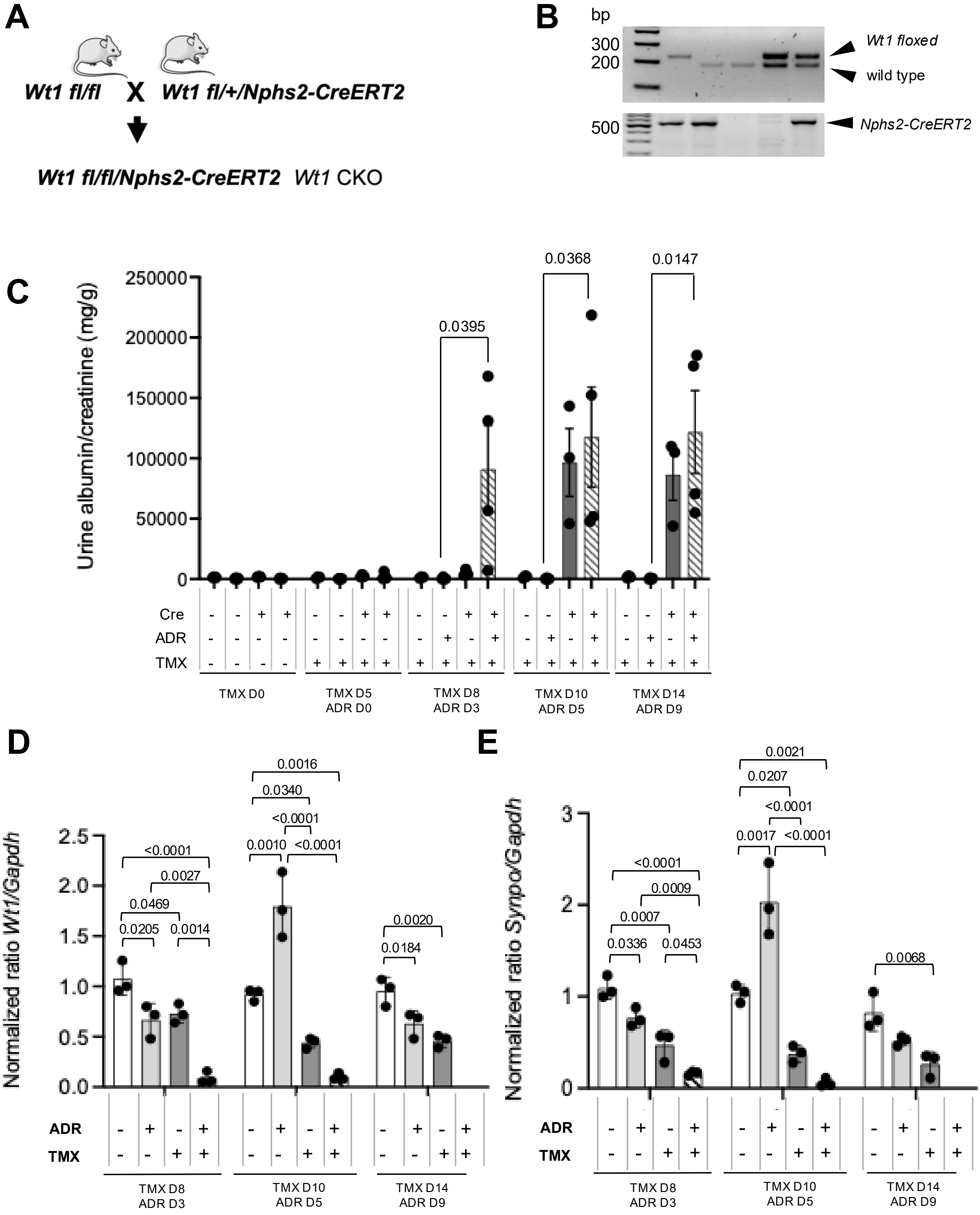

A
Wt1 fl/fl X Wt1 fl/+/Nphs2-CreERT2
Wt1 fl/fl/Nphs2-CreERT2 Wt1 CKO
B
bp
300
200
500
Wt1 floxed
wild type
Nphs2-CreERT2
C
Urine albumin/creatinine (mg/g)
250000
200000
150000
100000
50000
0
0.0395
0.0368
0.0147
Cre
ADR
TMX
TMX D0
TMX D5 ADR D0
TMX D8 ADR D3
TMX D10 ADR D5
TMX D14 ADR D9
D
Normalized ratio Wt1/Gapdh
2.5
2.0
1.5
1.0
0.5
0.0
<0.0001
0.0027
0.0469
0.0205
0.0014
0.0016
0.0340
<0.0001
0.0010
<0.0001
0.0020
0.0184
ADR
TMX
TMX D8 ADR D3
TMX D10 ADR D5
TMX D14 ADR D9
E
Normalized ratio Synpo/Gapdh
3
2
1
0
<0.0001
0.0009
0.0007
0.0336
0.0453
0.0021
0.0207
<0.0001
0.0017
<0.0001
0.0068
ADR
TMX
TMX D8 ADR D3
TMX D10 ADR D5
TMX D14 ADR D9

**Figure S5. Podocyte injury after conditional knockout of *Wt1.*** (A). Breeding scheme to obtain *Wt1 fl/fl/Nphs2-CreERT2.* (B) Genotyping example. (C) Urine albumin/creatinine ratios obtained from mice induced with tamoxifen and treated with ADR at the time points indicated below the graph. Data are presented as mean ± SD. One-way ANOVA with Tukey's multiple comparisons test. (D) RT-pPCR of *Wt1* expression in isolated glomeruli of mice at same time points, treatments as in (C). (E) Same as (B) except for *Synpo.* Data are presented as mean ± SD. One-way ANOVA with Tukey's multiple comparisons test.

# Figure S6

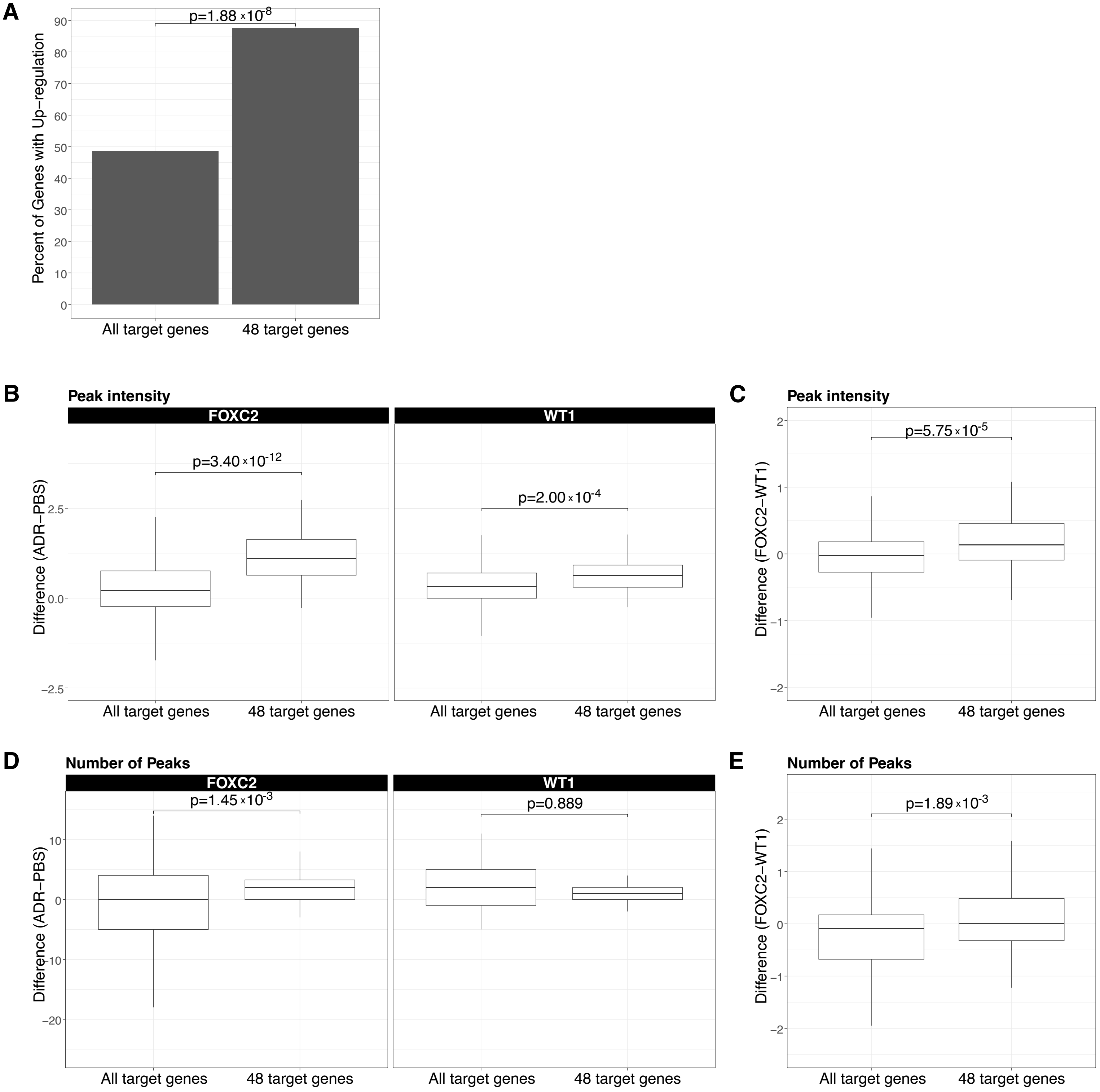
A
p=1.88 ×10-8
Percent of Genes with Up−regulation
90
80
70
60
50
40
30
20
10
0
All target genes
48 target genes
B
Peak intensity
FOXC2
WT1
p=3.40 ×10-12
p=2.00 ×10-4
Difference (ADR−PBS)
2.5
0.0
−2.5
All target genes
48 target genes
All target genes
48 target genes
C
Peak intensity
p=5.75 ×10-5
Difference (FOXC2−WT1)
2
1
0
−1
−2
All target genes
48 target genes
D
Number of Peaks
FOXC2
WT1
p=1.45 ×10-3
p=0.889
Difference (ADR−PBS)
10
0
−10
−20
All target genes
48 target genes
All target genes
48 target genes
E
Number of Peaks
p=1.89 ×10-3
Difference (FOXC2−WT1)
2
1
0
−1
−2
All target genes
48 target genes

**Figure S6. Comparison of FOXC2 and WT1 binding to a set of podocyte genes**. All plots compare all FOXC2 and/or WT1 target genes to the defined set of podocyte genes. (A) Bar plot of percent of genes with up-regulation. (B) Boxplot of difference between peak intensity of ADR and PBS (right). (C) Boxplot of difference between FOXC2 and WT1 peak intensity. (D) Boxplot of difference between number of peaks in ADR and PBS. (E) Boxplot of difference between FOXC2 and WT1 peak intensity. Wilcoxon rank sum tests were used to calculate p-values.

**Figure S7.** IGV plots of the gene set identified as characteristically expressed in podocytes. (Karaiskos et al 2018, reference in text). *FoxC2* and *Lmx1b* were not part of this published set and have been included here as WT1 and FOXC2 bound genes of interest as described in the text. WT1 ChIP-seq data has been previously published (*3*).

The chromosomal position is indicated at the top of each page.

From top to bottom of the IGV plots:

F_ADR: FOXC2 peak signature in ADR condition (red)

F_ADR_peaks: FOXC2 statistically significant called peaks in ADR condition

F_PBS: FOXC2 peak signature in PBS condition (blue)

F_PBS_peaks: FOXC2 statistically significant called peaks in PBS condition

W_ADR: WT1 peak signature in ADR condition (maroon)

W_ADR_peaks: WT1 statistically significant called peaks in ADR condition

W_PBS: WT1 peak signature in PBS condition (turquoise)

W_PBS_peaks: WT1 statistically significant called peaks in PBS condition

Consensus_peaks: union of all peaks called for FOXC2 and WT1 in both ADR and PBS conditions

For any particular gene, the scales are the same within the FOXC2 group and within the WT1 group but not between these two groups. The FOXC2 and WT1 scales vary gene to gene.

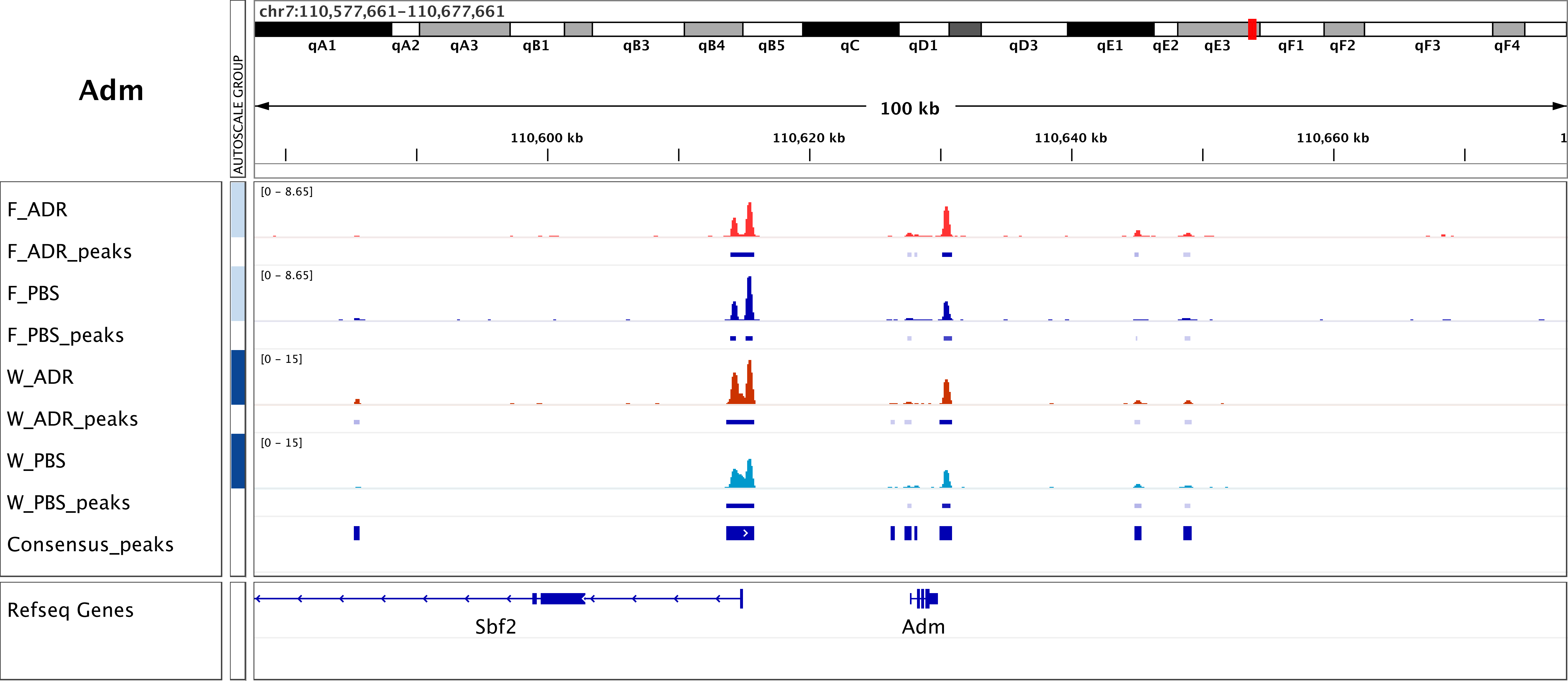

Adm
AUTOSCALE GROUP
chr7:110,577,661-110,677,661
qA1
qA2
qA3
qB1
qB3
qB4
qB5
qC
qD1
qD3
qE1
qE2
qE3
qF1
qF2
qF3
qF4
100 kb
110,600 kb
110,620 kb
110,640 kb
110,660 kb
F_ADR
[0 - 8.65]
F_ADR_peaks
F_PBS
[0 - 8.65]
F_PBS_peaks
W_ADR
[0 - 15]
W_ADR_peaks
W_PBS
[0 - 15]
W_PBS_peaks
Consensus_peaks
Refseq Genes
Sbf2
Adm

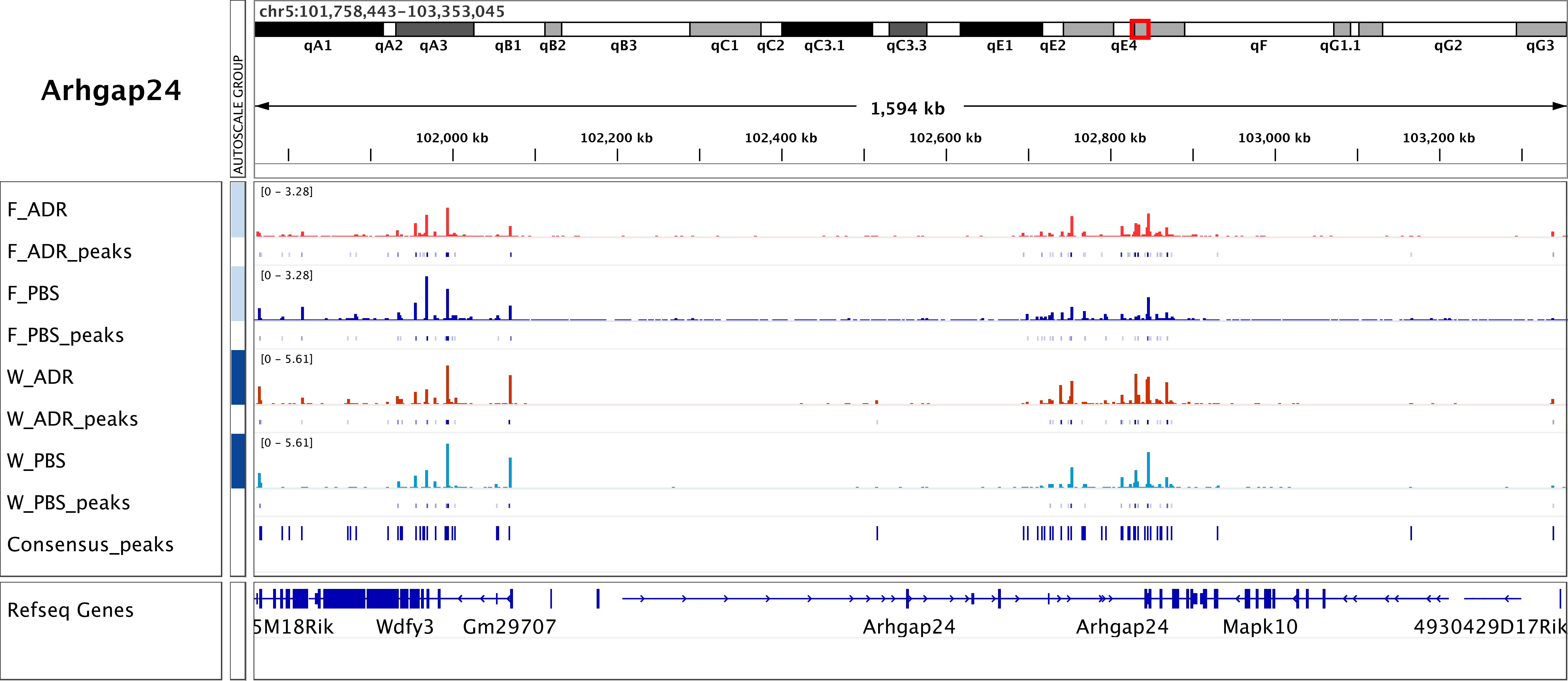

Arhgap24
AUTOSCALE GROUP
chr5:101,758,443-103,353,045
qA1
qA2
qA3
qB1
qB2
qB3
qC1
qC2
qC3.1
qC3.3
qE1
qE2
qE4
qF
qG1.1
qG2
qG3
1,594 kb
102,000 kb
102,200 kb
102,400 kb
102,600 kb
102,800 kb
103,000 kb
103,200 kb
F_ADR
[0 - 3.28]
F_ADR_peaks
F_PBS
[0 - 3.28]
F_PBS_peaks
W_ADR
[0 - 5.61]
W_ADR_peaks
W_PBS
[0 - 5.61]
W_PBS_peaks
Consensus_peaks
Refseq Genes
5M18Rik
Wdfy3
Gm29707
Arhgap24
Arhgap24
Mapk10
4930429D17Rik

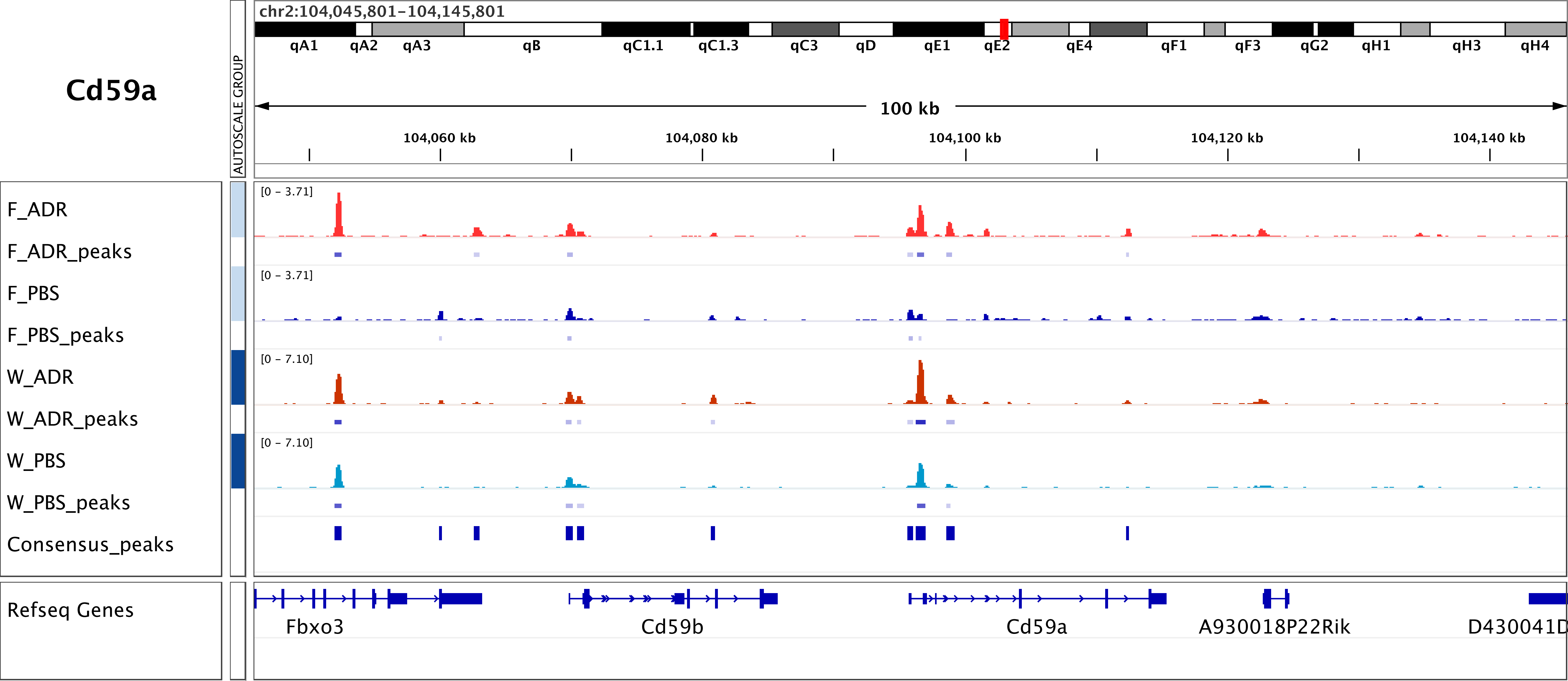
Cd59a
AUTOSCALE GROUP
chr2:104,045,801-104,145,801
qA1
qA2
qA3
qB
qC1.1
qC1.3
qC3
qD
qE1
qE2
qE4
qF1
qF3
qG2
qH1
qH3
qH4
100 kb
104,060 kb
104,080 kb
104,100 kb
104,120 kb
104,140 kb
F_ADR
F_ADR_peaks
F_PBS
F_PBS_peaks
W_ADR
W_ADR_peaks
W_PBS
W_PBS_peaks
Consensus_peaks
[0 - 3.71]
[0 - 3.71]
[0 - 7.10]
[0 - 7.10]
Refseq Genes
Fbxo3
Cd59b
Cd59a
A930018P22Rik
D430041D

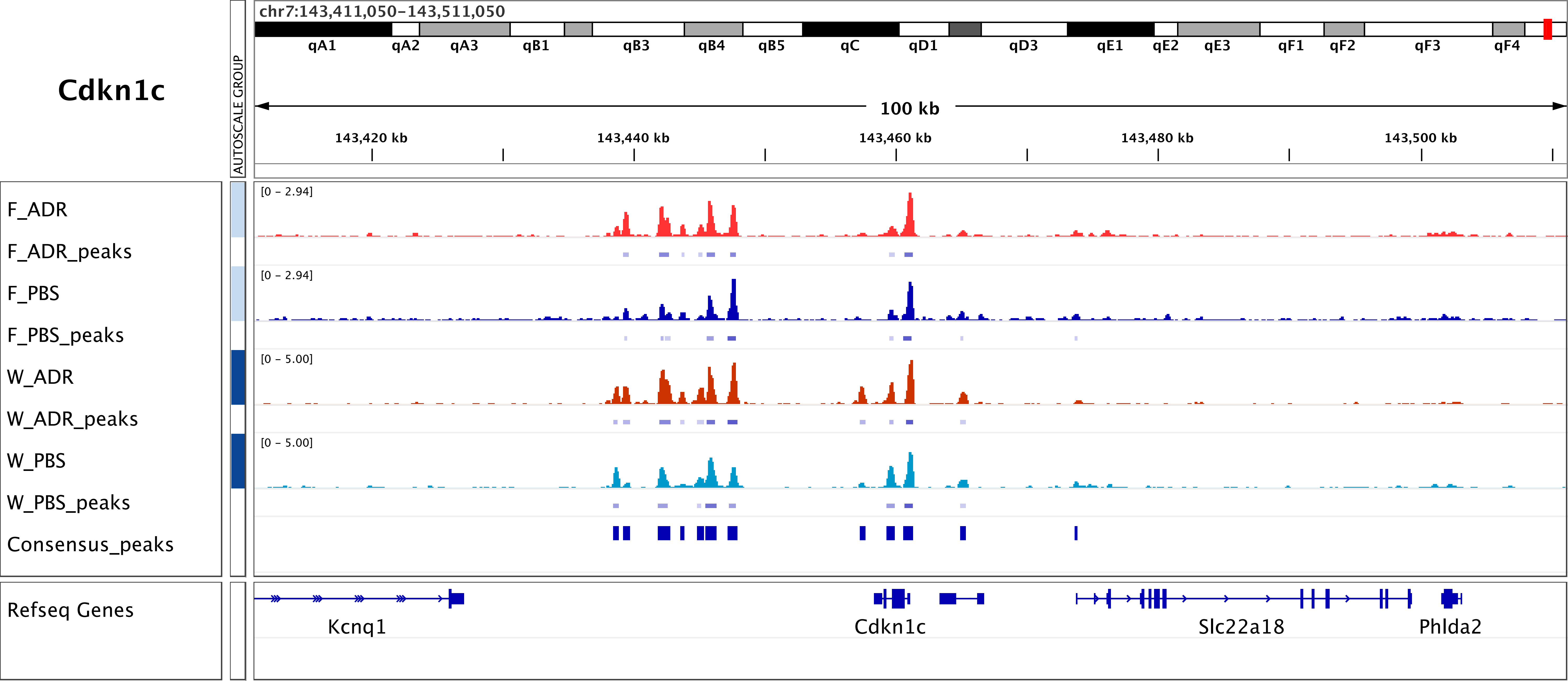
Cdkn1c
AUTOSCALE GROUP
chr7:143,411,050-143,511,050
qA1
qA2
qA3
qB1
qB3
qB4
qB5
qC
qD1
qD3
qE1
qE2
qE3
qF1
qF2
qF3
qF4
100 kb
143,420 kb
143,440 kb
143,460 kb
143,480 kb
143,500 kb
F_ADR
[0 - 2.94]
F_ADR_peaks
F_PBS
[0 - 2.94]
F_PBS_peaks
W_ADR
[0 - 5.00]
W_ADR_peaks
W_PBS
[0 - 5.00]
W_PBS_peaks
Consensus_peaks
Refseq Genes
Kcnq1
Cdkn1c
Slc22a18
Phlda2

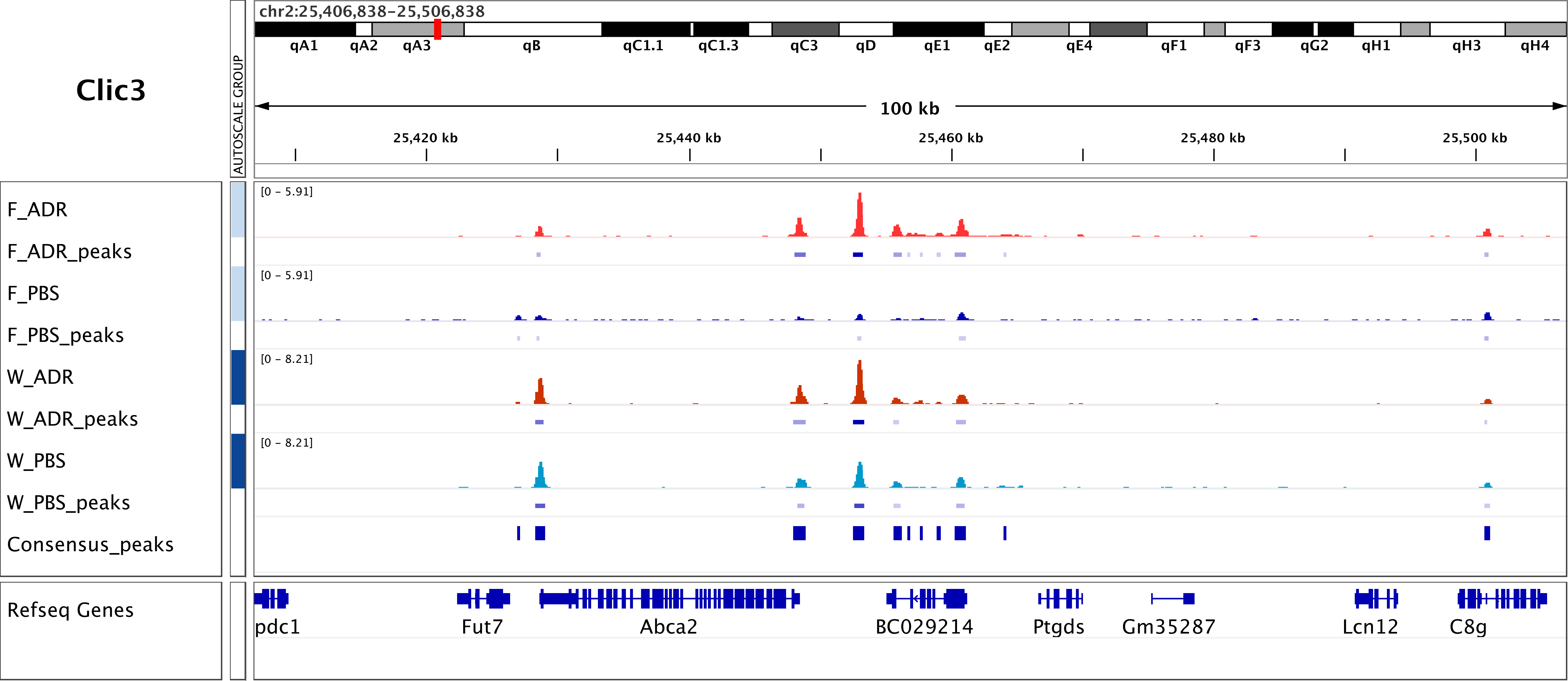
Clic3
chr2:25,406,838-25,506,838
qA1
qA2
qA3
qB
qC1.1
qC1.3
qC3
qD
qE1
qE2
qE4
qF1
qF3
qG2
qH1
qH3
qH4
100 kb
25,420 kb
25,440 kb
25,460 kb
25,480 kb
25,500 kb
AUTOSCALE GROUP
F_ADR
[0 - 5.91]
F_ADR_peaks
F_PBS
[0 - 5.91]
F_PBS_peaks
W_ADR
[0 - 8.21]
W_ADR_peaks
W_PBS
[0 - 8.21]
W_PBS_peaks
Consensus_peaks
Refseq Genes
pdc1
Fut7
Abca2
BC029214
Ptgds
Gm35287
Lcn12
C8g

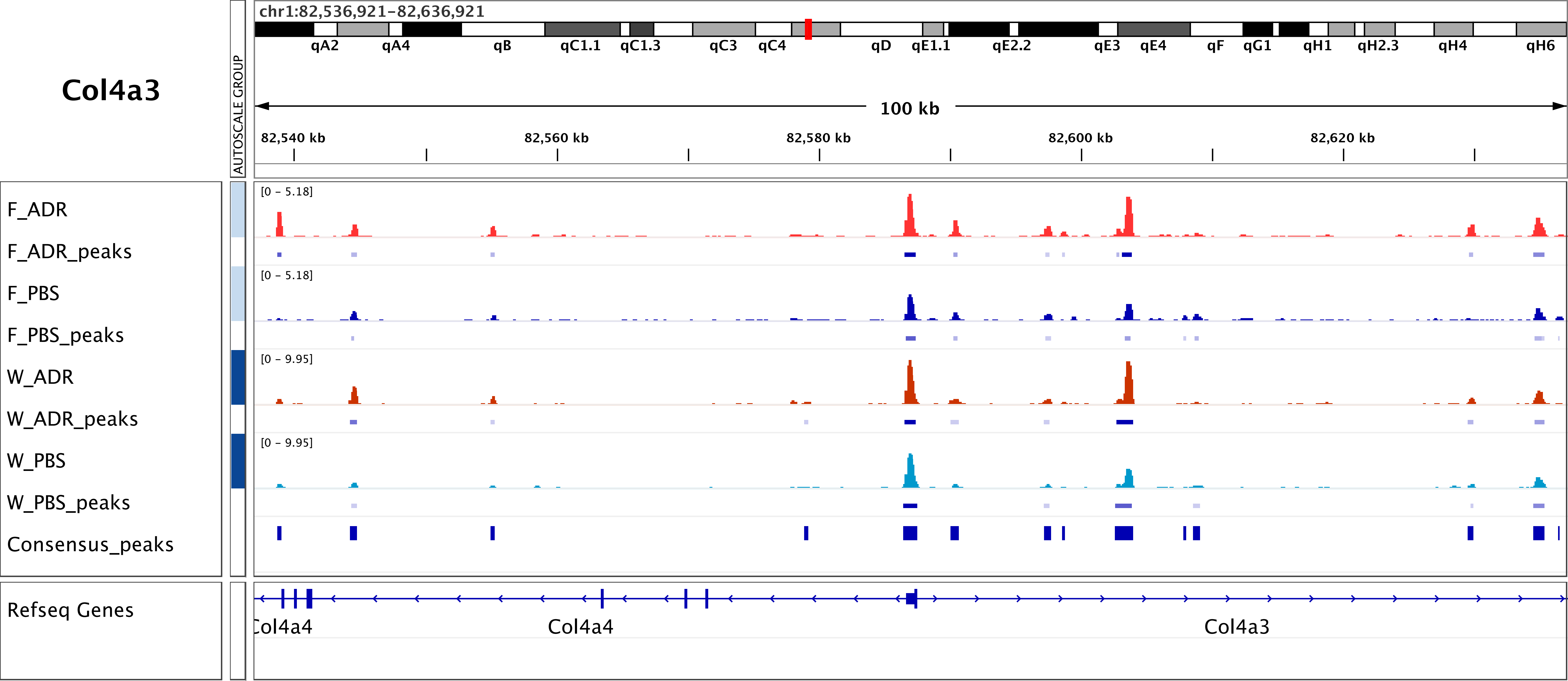

Col4a3
AUTOSCALE GROUP
chr1:82,536,921-82,636,921
qA2
qA4
qB
qC1.1
qC1.3
qC3
qC4
qD
qE1.1
qE2.2
qE3
qE4
qF
qG1
qH1
qH2.3
qH4
qH6
100 kb
82,540 kb
82,560 kb
82,580 kb
82,600 kb
82,620 kb
F_ADR
[0 - 5.18]
F_ADR_peaks
F_PBS
[0 - 5.18]
F_PBS_peaks
W_ADR
[0 - 9.95]
W_ADR_peaks
W_PBS
[0 - 9.95]
W_PBS_peaks
Consensus_peaks
Refseq Genes
Col4a4
Col4a4
Col4a3

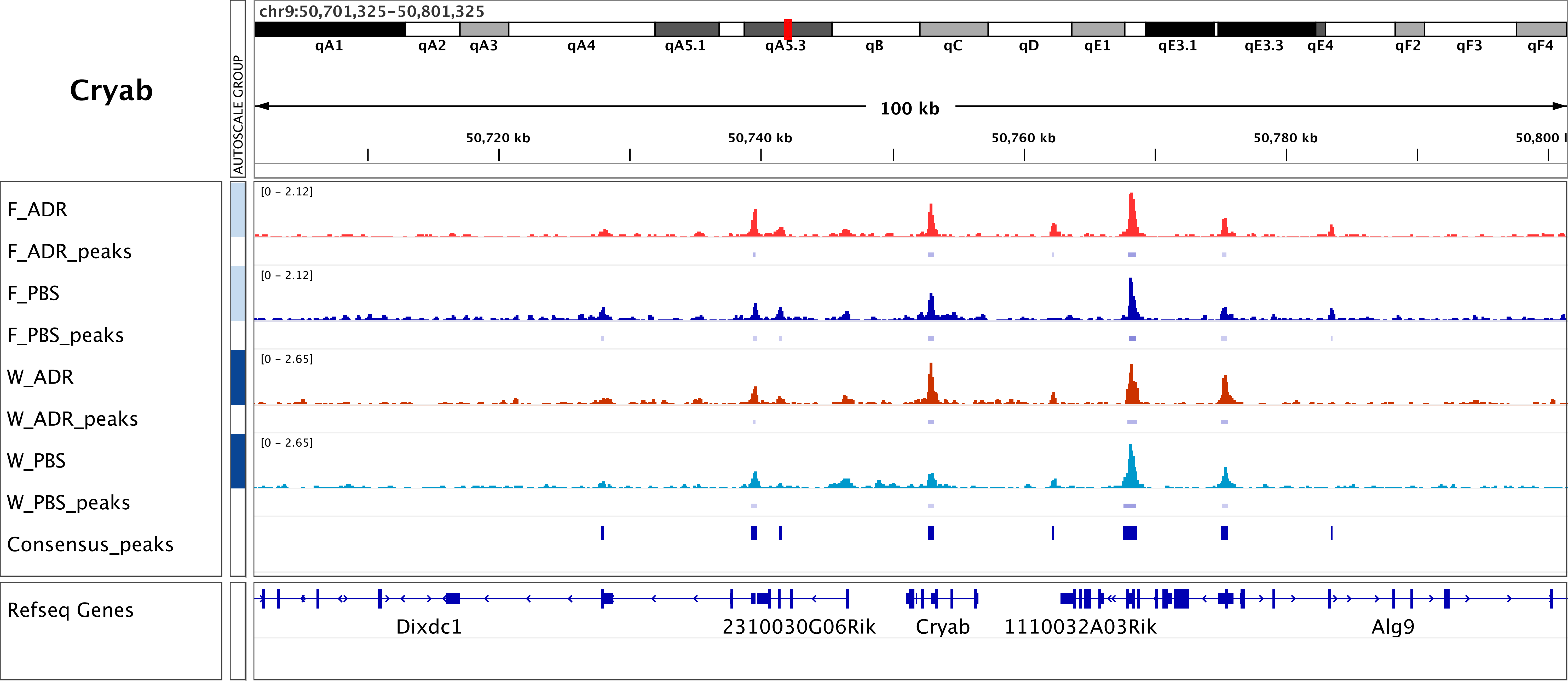

Cryab
AUTOSCALE GROUP
chr9:50,701,325-50,801,325
qA1
qA2
qA3
qA4
qA5.1
qA5.3
qB
qC
qD
qE1
qE3.1
qE3.3
qE4
qF2
qF3
qF4
100 kb
50,720 kb
50,740 kb
50,760 kb
50,780 kb
50,800 kb
F_ADR
F_ADR_peaks
F_PBS
F_PBS_peaks
W_ADR
W_ADR_peaks
W_PBS
W_PBS_peaks
Consensus_peaks
[0 - 2.12]
[0 - 2.12]
[0 - 2.65]
[0 - 2.65]
Refseq Genes
Dixdc1
2310030G06Rik
Cryab
1110032A03Rik
Alg9

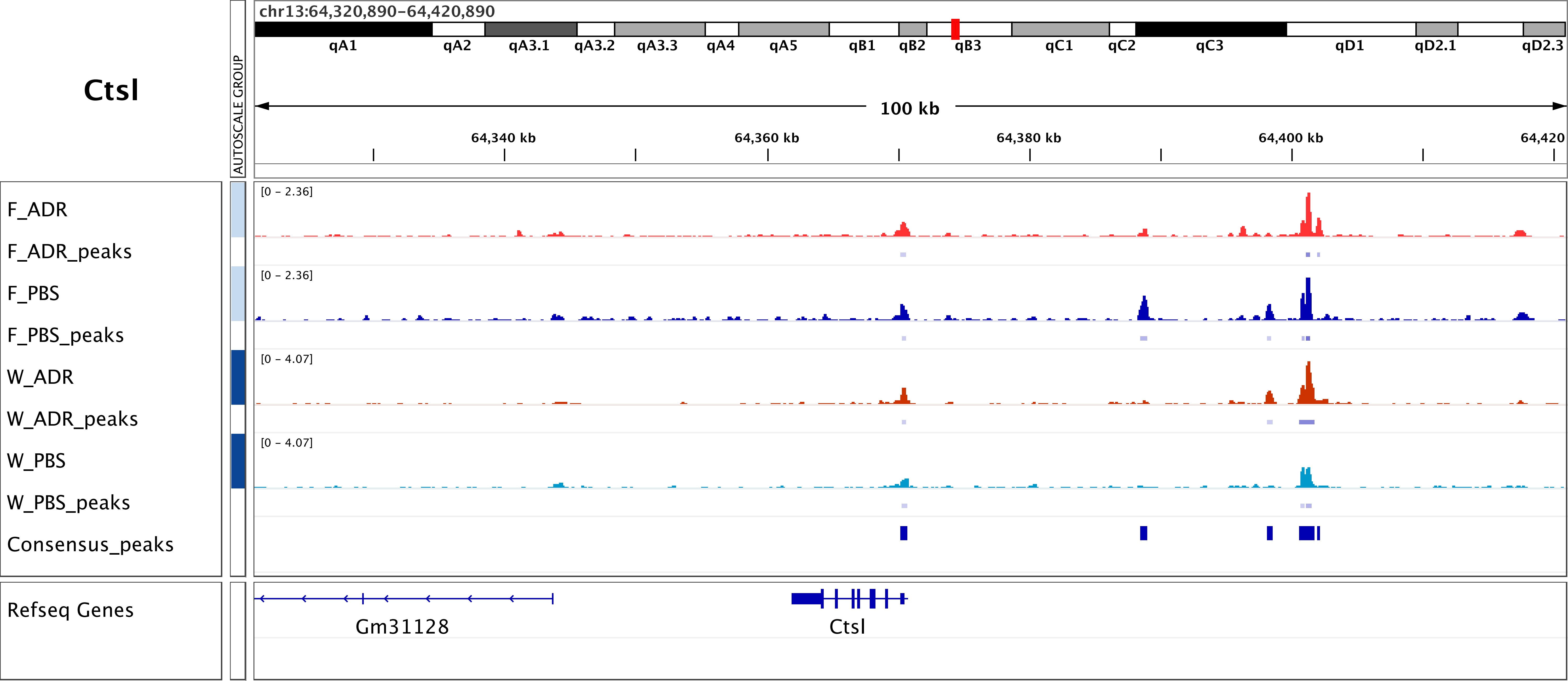
Ctsl
AUTOSCALE GROUP
chr13:64,320,890-64,420,890
qA1
qA2
qA3.1
qA3.2
qA3.3
qA4
qA5
qB1
qB2
qB3
qC1
qC2
qC3
qD1
qD2.1
qD2.3
100 kb
64,340 kb
64,360 kb
64,380 kb
64,400 kb
64,420
F_ADR
[0 - 2.36]
F_ADR_peaks
F_PBS
[0 - 2.36]
F_PBS_peaks
W_ADR
[0 - 4.07]
W_ADR_peaks
W_PBS
[0 - 4.07]
W_PBS_peaks
Consensus_peaks
Refseq Genes
Gm31128
Ctsl

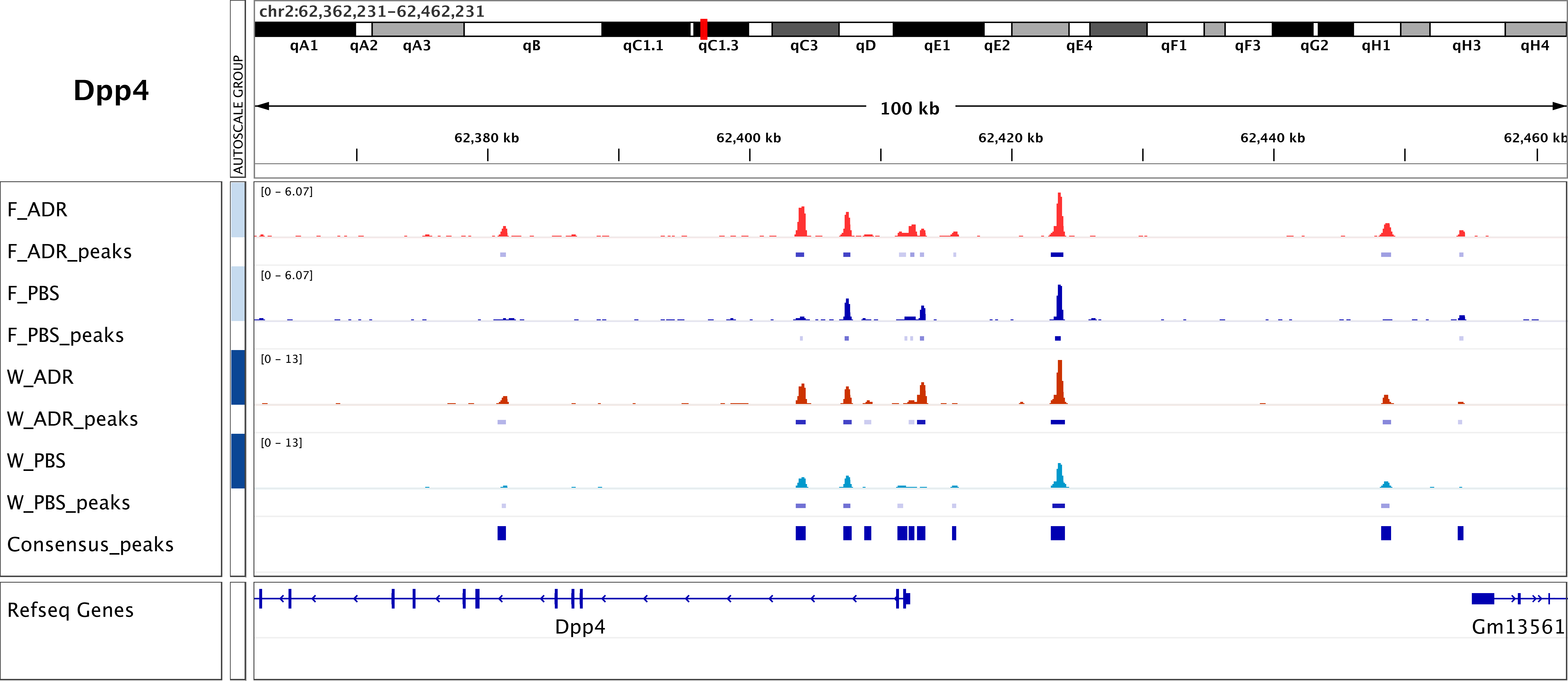
Dpp4
AUTOSCALE GROUP
chr2:62,362,231-62,462,231
qA1
qA2
qA3
qB
qC1.1
qC1.3
qC3
qD
qE1
qE2
qE4
qF1
qF3
qG2
qH1
qH3
qH4
100 kb
62,380 kb
62,400 kb
62,420 kb
62,440 kb
62,460 kb
F_ADR
F_ADR_peaks
F_PBS
F_PBS_peaks
W_ADR
W_ADR_peaks
W_PBS
W_PBS_peaks
Consensus_peaks
[0 - 6.07]
[0 - 6.07]
[0 - 13]
[0 - 13]
Refseq Genes
Dpp4
Gm13561

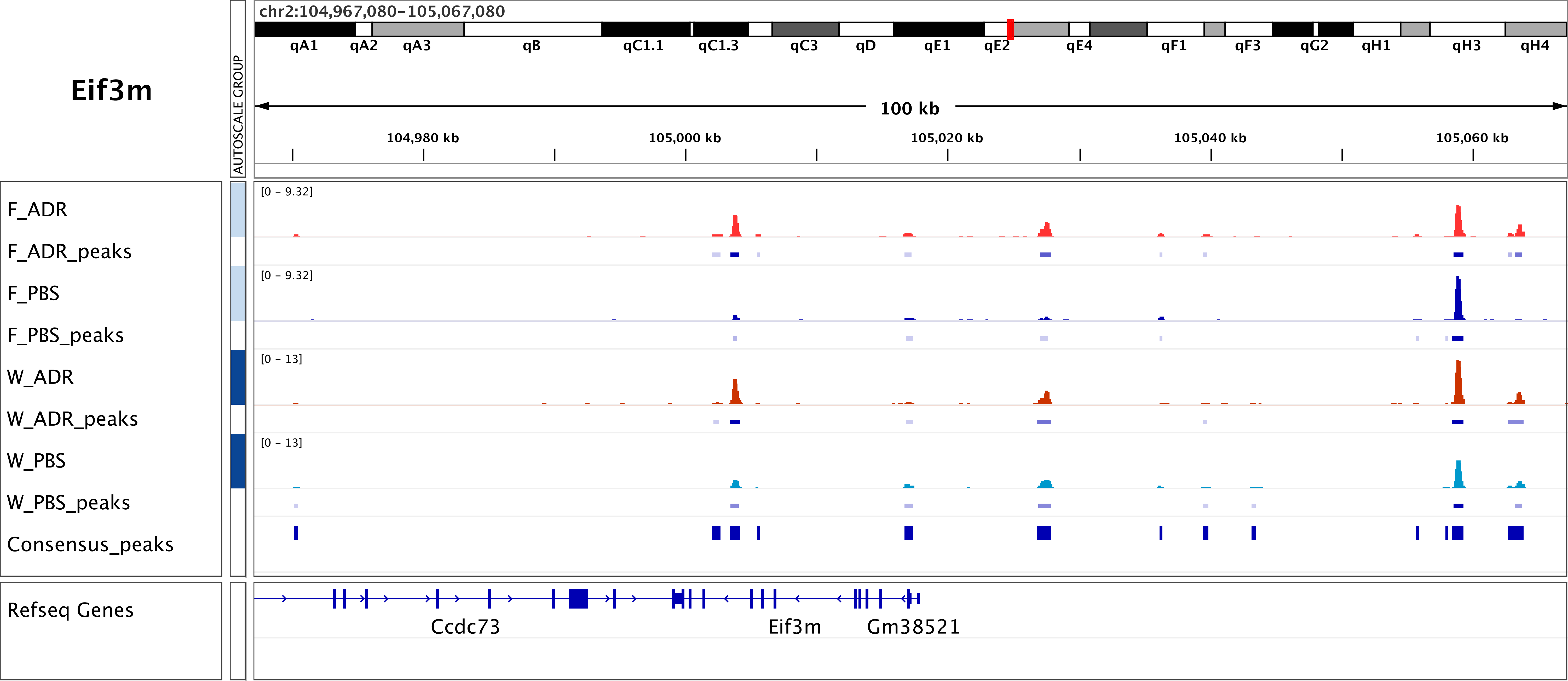

Eif3m
AUTOSCALE GROUP
chr2:104,967,080-105,067,080
qA1
qA2
qA3
qB
qC1.1
qC1.3
qC3
qD
qE1
qE2
qE4
qF1
qF3
qG2
qH1
qH3
qH4
100 kb
104,980 kb
105,000 kb
105,020 kb
105,040 kb
105,060 kb
F_ADR
[0 - 9.32]
F_ADR_peaks
F_PBS
[0 - 9.32]
F_PBS_peaks
W_ADR
[0 - 13]
W_ADR_peaks
W_PBS
[0 - 13]
W_PBS_peaks
Consensus_peaks
Refseq Genes
Ccdc73
Eif3m
Gm38521

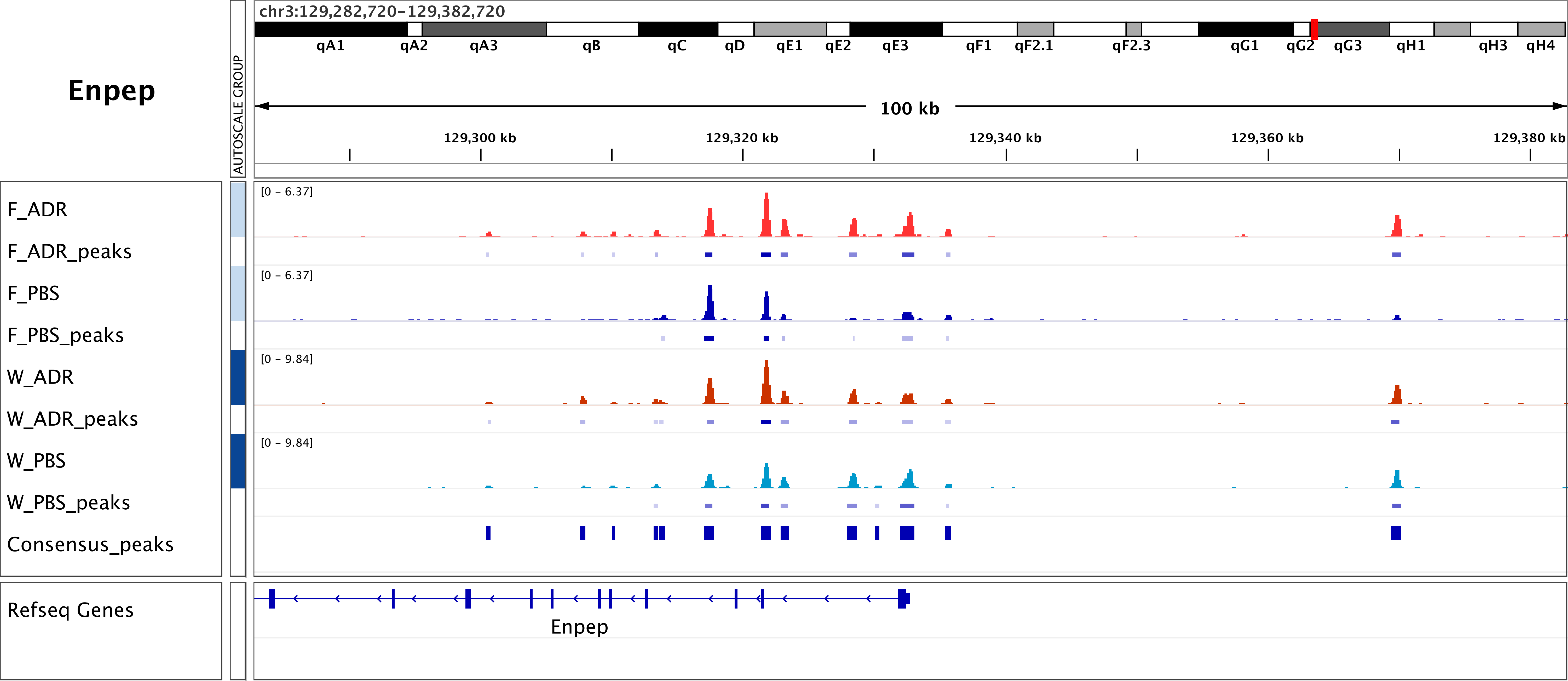

Enpep
AUTOSCALE GROUP
chr3:129,282,720-129,382,720
qA1
qA2
qA3
qB
qC
qD
qE1
qE2
qE3
qF1
qF2.1
qF2.3
qG1
qG2
qG3
qH1
qH3
qH4
100 kb
129,300 kb
129,320 kb
129,340 kb
129,360 kb
129,380 kb
F_ADR
F_ADR_peaks
F_PBS
F_PBS_peaks
W_ADR
W_ADR_peaks
W_PBS
W_PBS_peaks
Consensus_peaks
[0 - 6.37]
[0 - 6.37]
[0 - 9.84]
[0 - 9.84]
Refseq Genes
Enpep

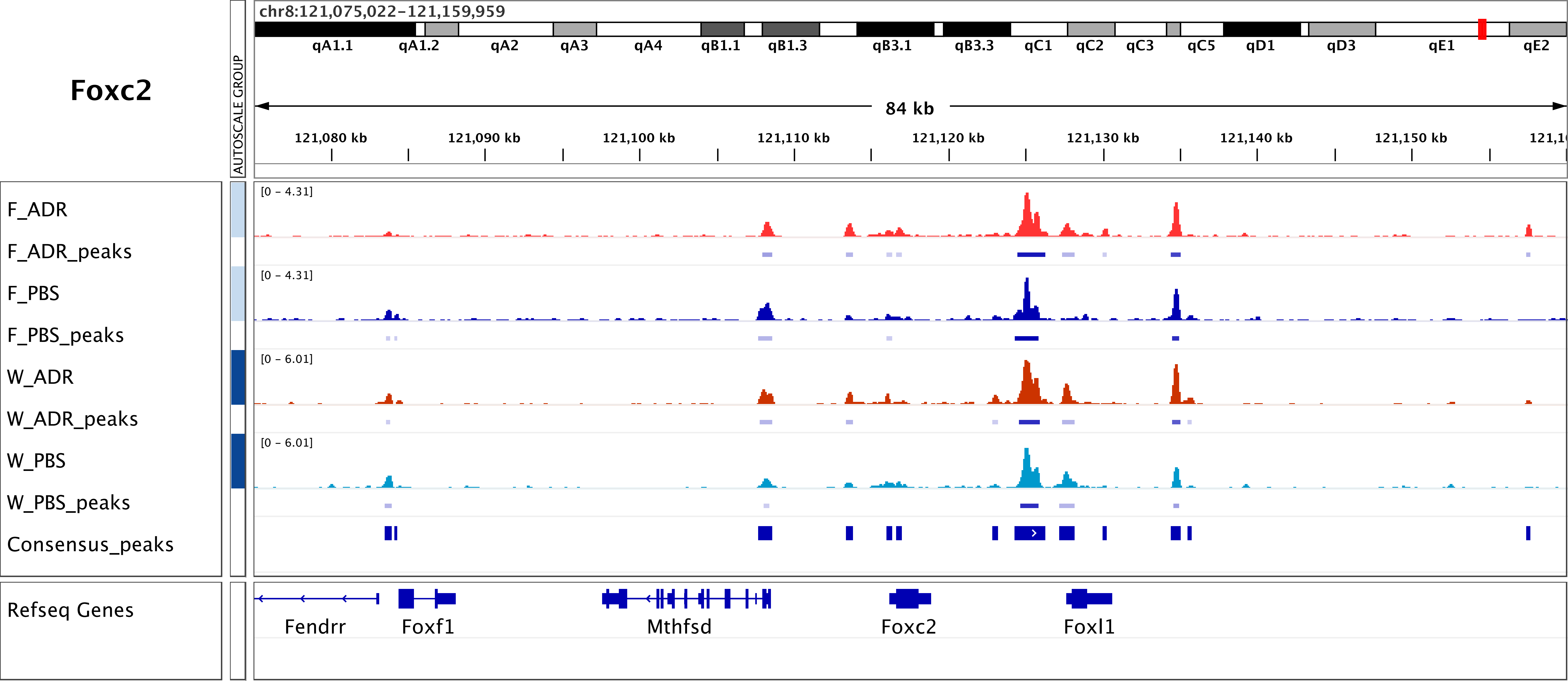

Foxc2
AUTOSCALE GROUP
chr8:121,075,022-121,159,959
qA1.1 qA1.2 qA2 qA3 qA4 qB1.1 qB1.3 qB3.1 qB3.3 qC1 qC2 qC3 qC5 qD1 qD3 qE1 qE2
84 kb
121,080 kb 121,090 kb 121,100 kb 121,110 kb 121,120 kb 121,130 kb 121,140 kb 121,150 kb
F_ADR
[0 - 4.31]
F_ADR_peaks
F_PBS
[0 - 4.31]
F_PBS_peaks
W_ADR
[0 - 6.01]
W_ADR_peaks
W_PBS
[0 - 6.01]
W_PBS_peaks
Consensus_peaks
Refseq Genes
Fendrr
Foxf1
Mthfsd
Foxc2
Foxl1

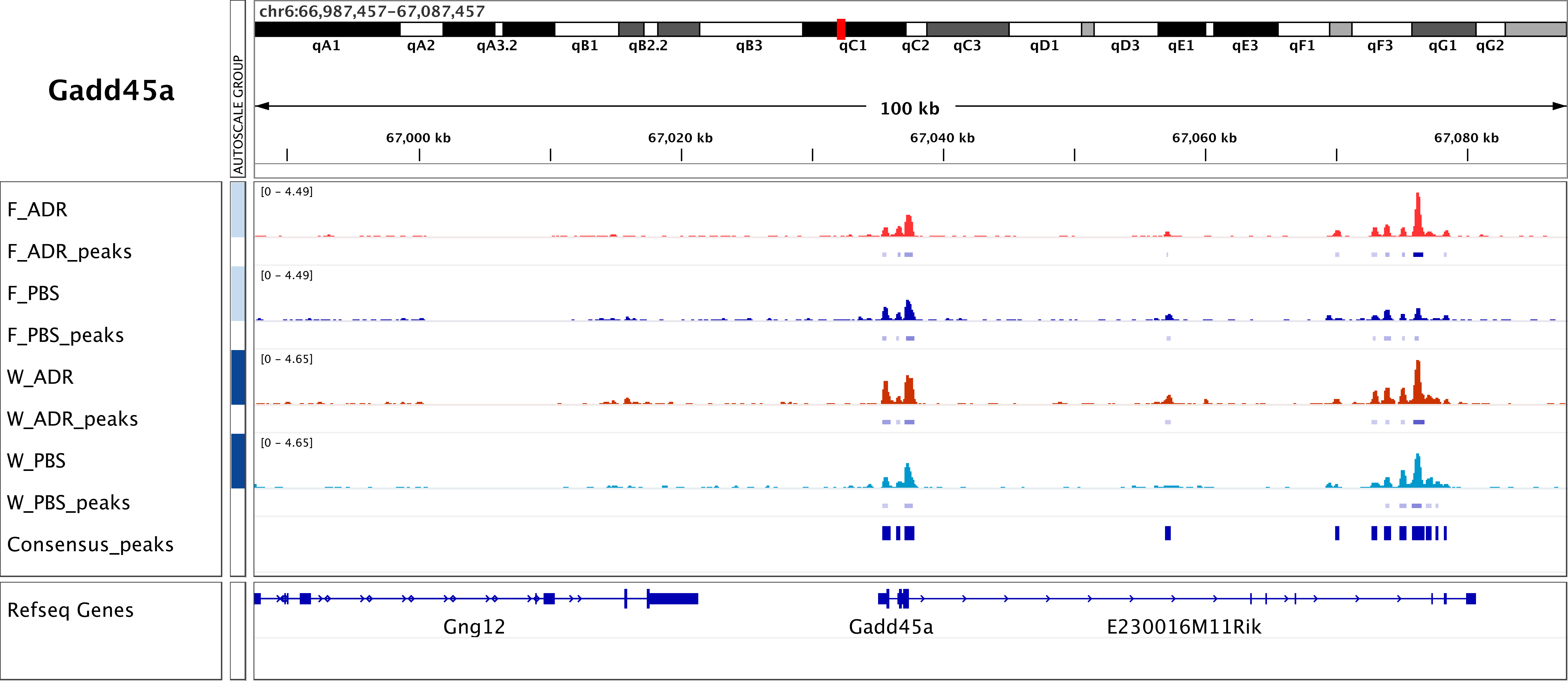

Gadd45a
AUTOSCALE GROUP
chr6:66,987,457-67,087,457
qA1
qA2
qA3.2
qB1
qB2.2
qB3
qC1
qC2
qC3
qD1
qD3
qE1
qE3
qF1
qF3
qG1
qG2
100 kb
67,000 kb
67,020 kb
67,040 kb
67,060 kb
67,080 kb
F_ADR
[0 - 4.49]
F_ADR_peaks
F_PBS
[0 - 4.49]
F_PBS_peaks
W_ADR
[0 - 4.65]
W_ADR_peaks
W_PBS
[0 - 4.65]
W_PBS_peaks
Consensus_peaks
Refseq Genes
Gng12
Gadd45a
E230016M11Rik

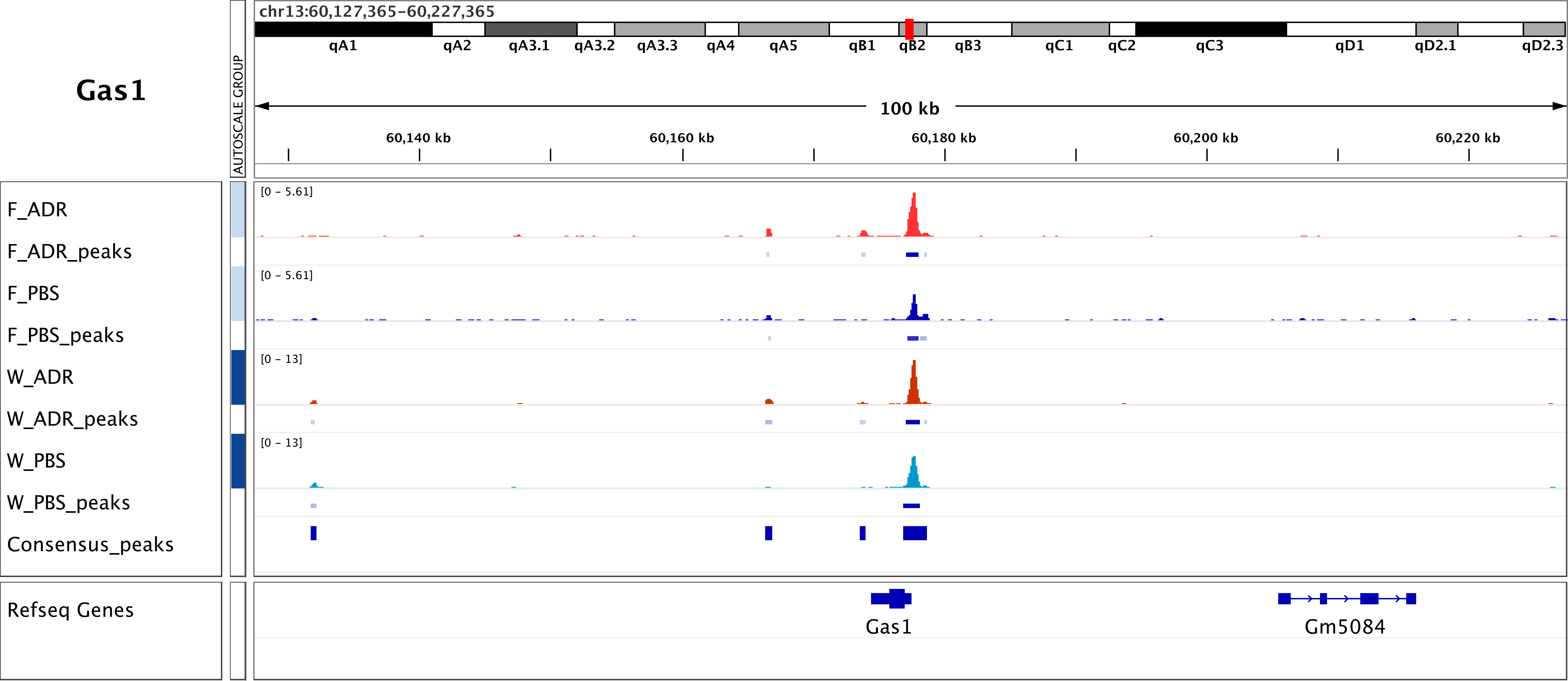
Gas1
AUTOSCALE GROUP
chr13:60,127,365-60,227,365
qA1
qA2
qA3.1
qA3.2
qA3.3
qA4
qA5
qB1
qB2
qB3
qC1
qC2
qC3
qD1
qD2.1
qD2.3
100 kb
60,140 kb
60,160 kb
60,180 kb
60,200 kb
60,220 kb
F_ADR
F_ADR_peaks
F_PBS
F_PBS_peaks
W_ADR
W_ADR_peaks
W_PBS
W_PBS_peaks
Consensus_peaks
[0 - 5.61]
[0 - 5.61]
[0 - 13]
[0 - 13]
Refseq Genes
Gas1
Gm5084

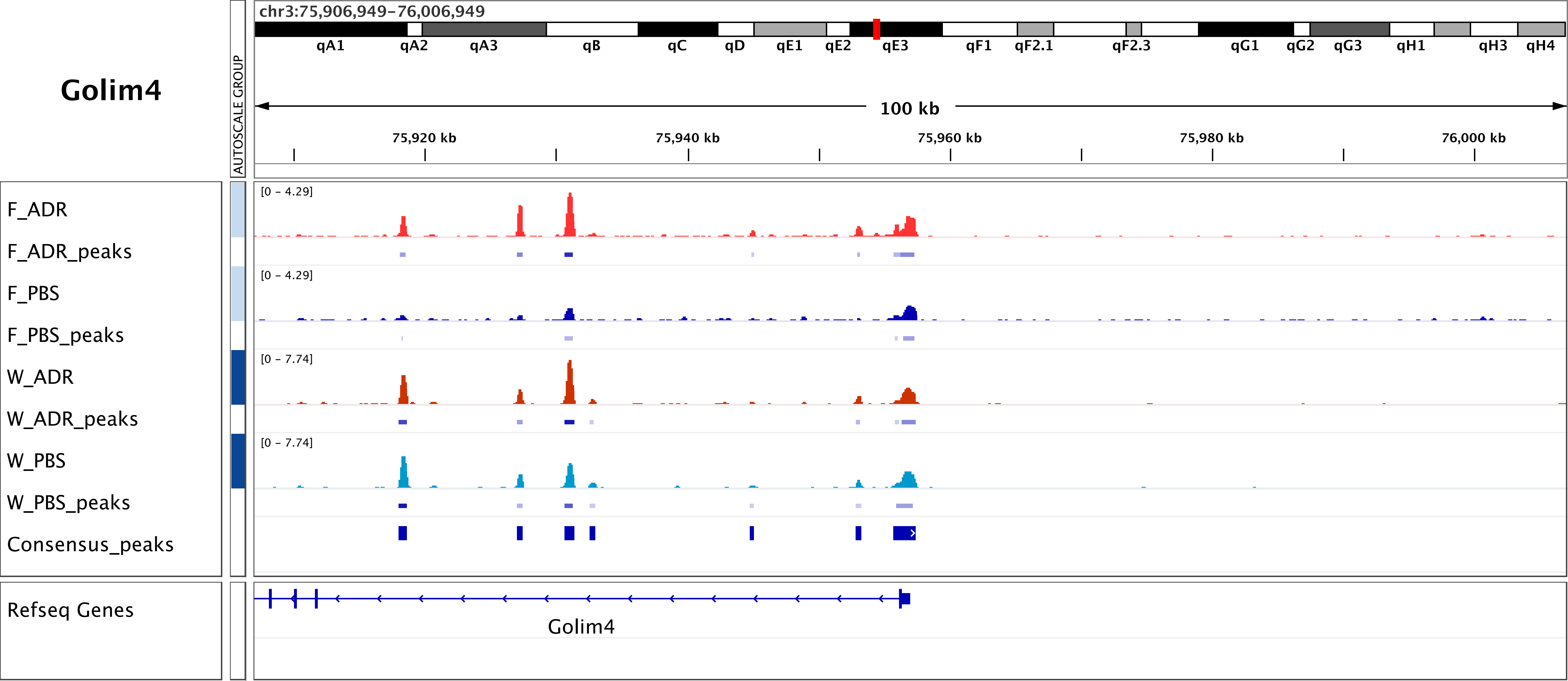
Golim4
AUTOSCALE GROUP
chr3:75,906,949-76,006,949
qA1
qA2
qA3
qB
qC
qD
qE1
qE2
qE3
qF1
qF2.1
qF2.3
qG1
qG2
qG3
qH1
qH3
qH4
100 kb
75,920 kb
75,940 kb
75,960 kb
75,980 kb
76,000 kb
F_ADR
[0 - 4.29]
F_ADR_peaks
F_PBS
[0 - 4.29]
F_PBS_peaks
W_ADR
[0 - 7.74]
W_ADR_peaks
W_PBS
[0 - 7.74]
W_PBS_peaks
Consensus_peaks
Refseq Genes
Golim4

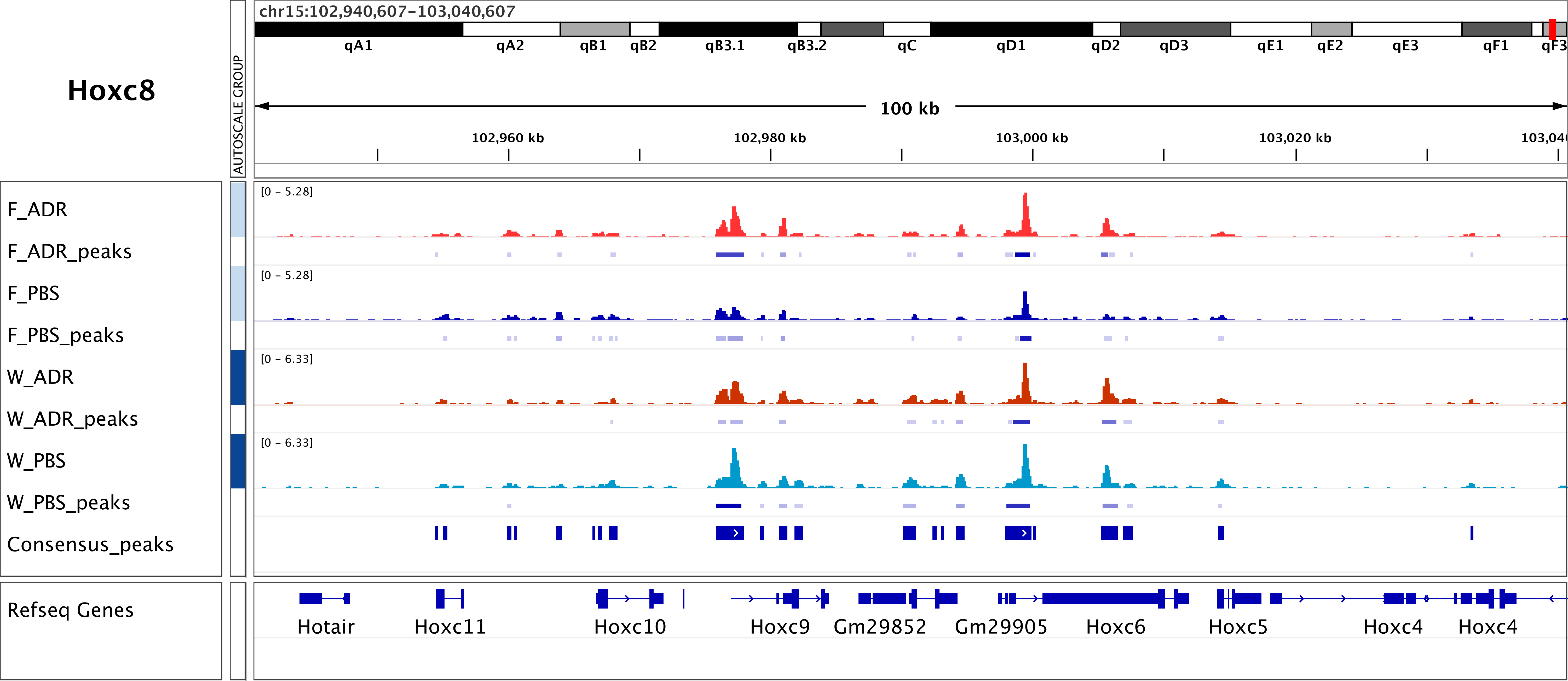

Hoxc8
chr15:102,940,607-103,040,607
qA1
qA2
qB1
qB2
qB3.1
qB3.2
qC
qD1
qD2
qD3
qE1
qE2
qE3
qF1
qF3
100 kb
102,960 kb
102,980 kb
103,000 kb
103,020 kb
AUTOSCALE GROUP
F_ADR
F_ADR_peaks
F_PBS
F_PBS_peaks
W_ADR
W_ADR_peaks
W_PBS
W_PBS_peaks
Consensus_peaks
[0 - 5.28]
[0 - 5.28]
[0 - 6.33]
[0 - 6.33]
Refseq Genes
Hotair
Hoxc11
Hoxc10
Hoxc9
Gm29852
Gm29905
Hoxc6
Hoxc5
Hoxc4
Hoxc4

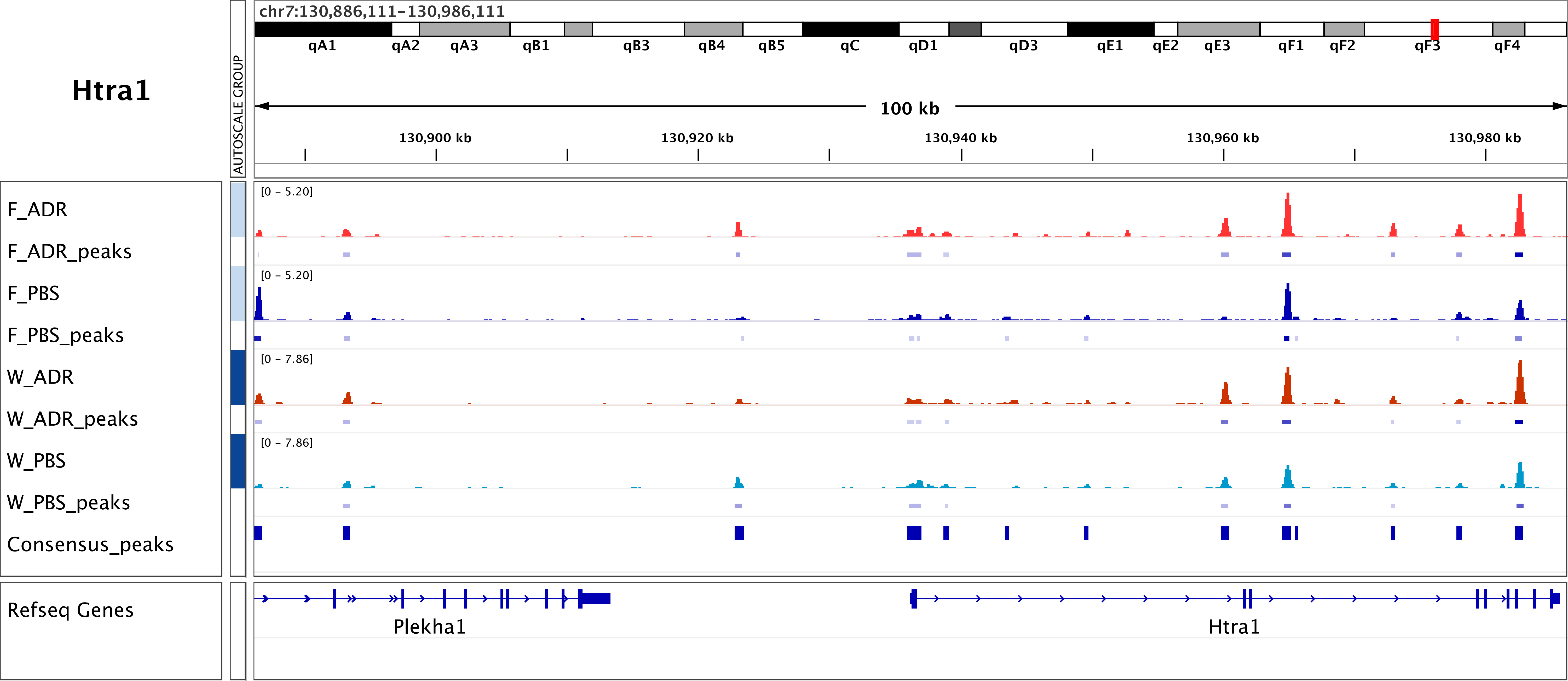

Htra1
AUTOSCALE GROUP
chr7:130,886,111-130,986,111
qA1
qA2
qA3
qB1
qB3
qB4
qB5
qC
qD1
qD3
qE1
qE2
qE3
qF1
qF2
qF3
qF4
100 kb
130,900 kb
130,920 kb
130,940 kb
130,960 kb
130,980 kb
F_ADR
F_ADR_peaks
F_PBS
F_PBS_peaks
W_ADR
W_ADR_peaks
W_PBS
W_PBS_peaks
Consensus_peaks
[0 - 5.20]
[0 - 5.20]
[0 - 7.86]
[0 - 7.86]
Refseq Genes
Plekha1
Htra1

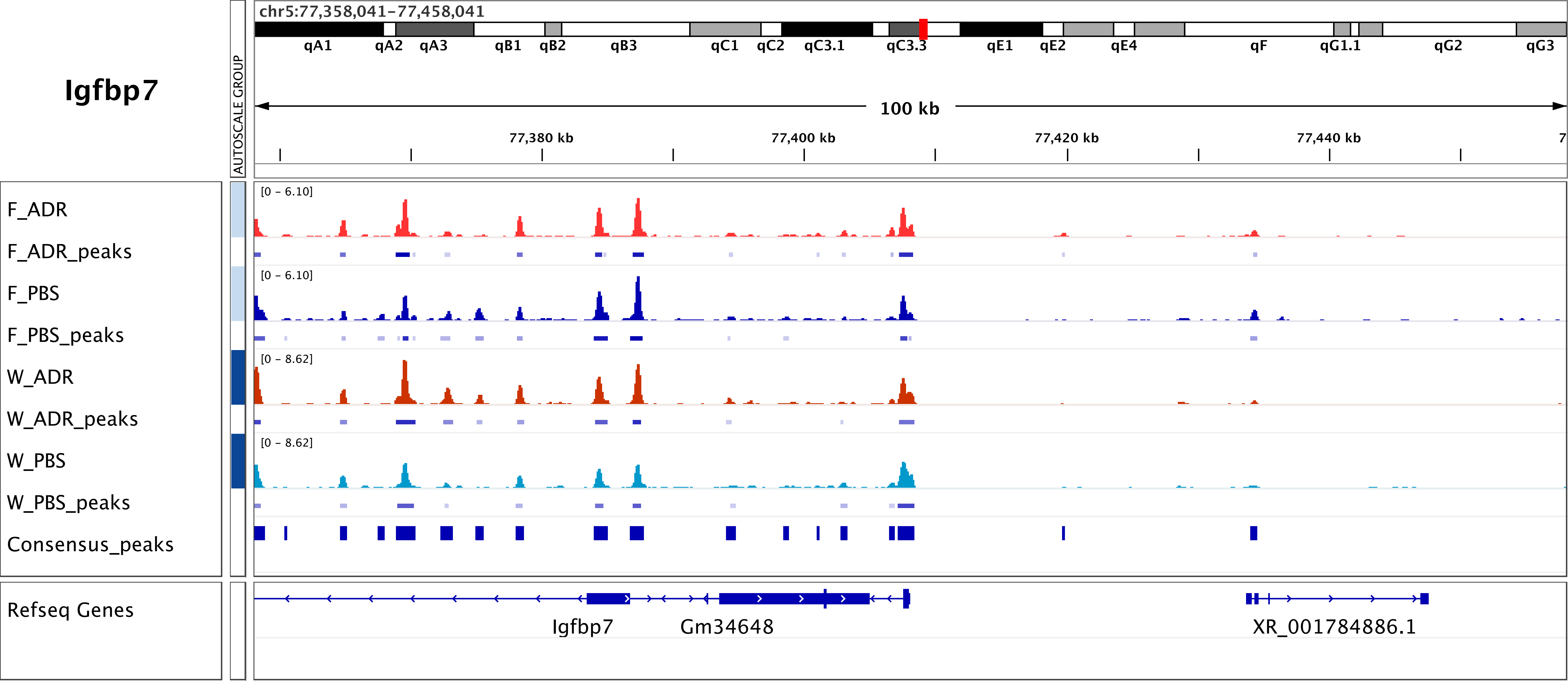

Igfbp7
AUTOSCALE GROUP
chr5:77,358,041-77,458,041
qA1
qA2
qA3
qB1
qB2
qB3
qC1
qC2
qC3.1
qC3.3
qE1
qE2
qE4
qF
qG1.1
qG2
qG3
100 kb
77,380 kb
77,400 kb
77,420 kb
77,440 kb
F_ADR
F_ADR_peaks
F_PBS
F_PBS_peaks
W_ADR
W_ADR_peaks
W_PBS
W_PBS_peaks
Consensus_peaks
[0 - 6.10]
[0 - 6.10]
[0 - 8.62]
[0 - 8.62]
Refseq Genes
Igfbp7
Gm34648
XR_001784886.1

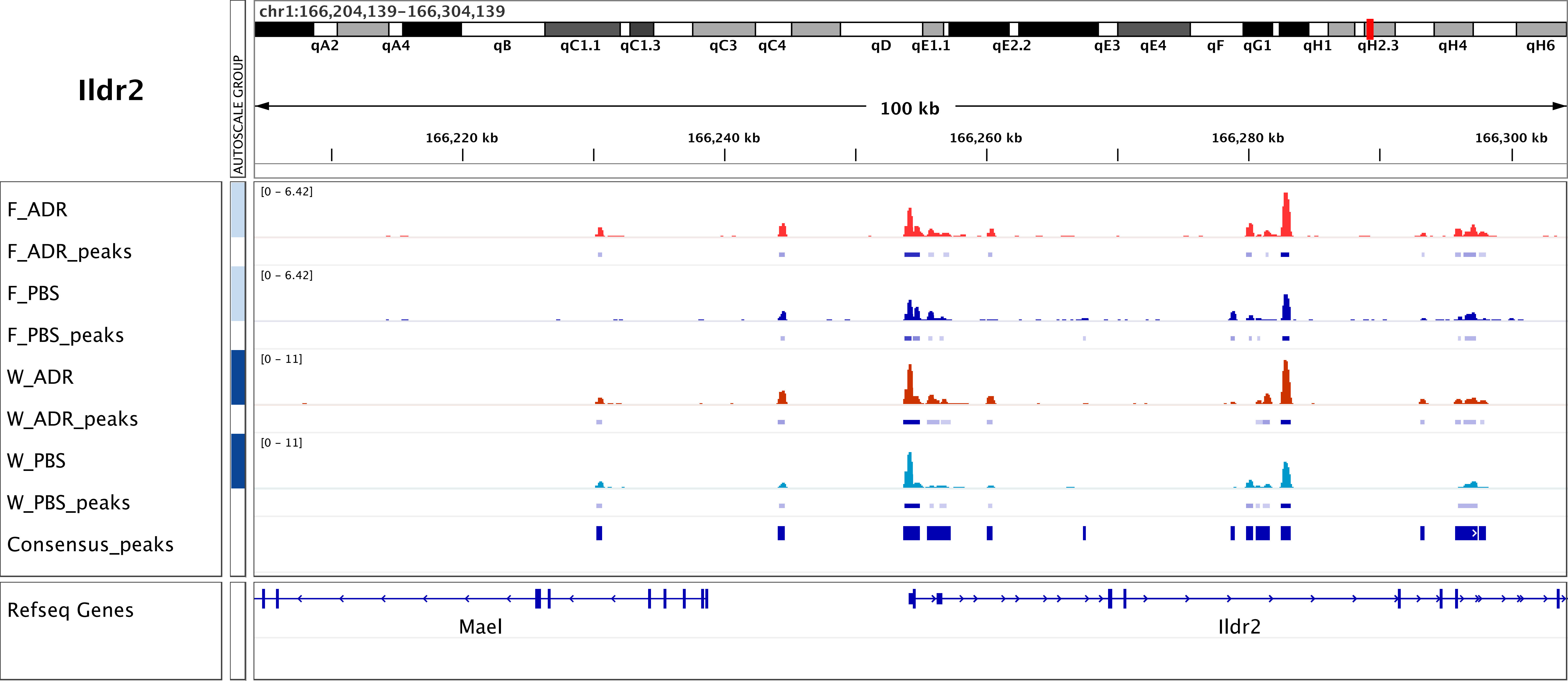

Ildr2
AUTOSCALE GROUP
chr1:166,204,139-166,304,139
qA2
qA4
qB
qC1.1
qC1.3
qC3
qC4
qD
qE1.1
qE2.2
qE3
qE4
qF
qG1
qH1
qH2.3
qH4
qH6
100 kb
166,220 kb
166,240 kb
166,260 kb
166,280 kb
166,300 kb
F_ADR
F_ADR_peaks
F_PBS
F_PBS_peaks
W_ADR
W_ADR_peaks
W_PBS
W_PBS_peaks
Consensus_peaks
[0 - 6.42]
[0 - 6.42]
[0 - 11]
[0 - 11]
Refseq Genes
Mael
Ildr2

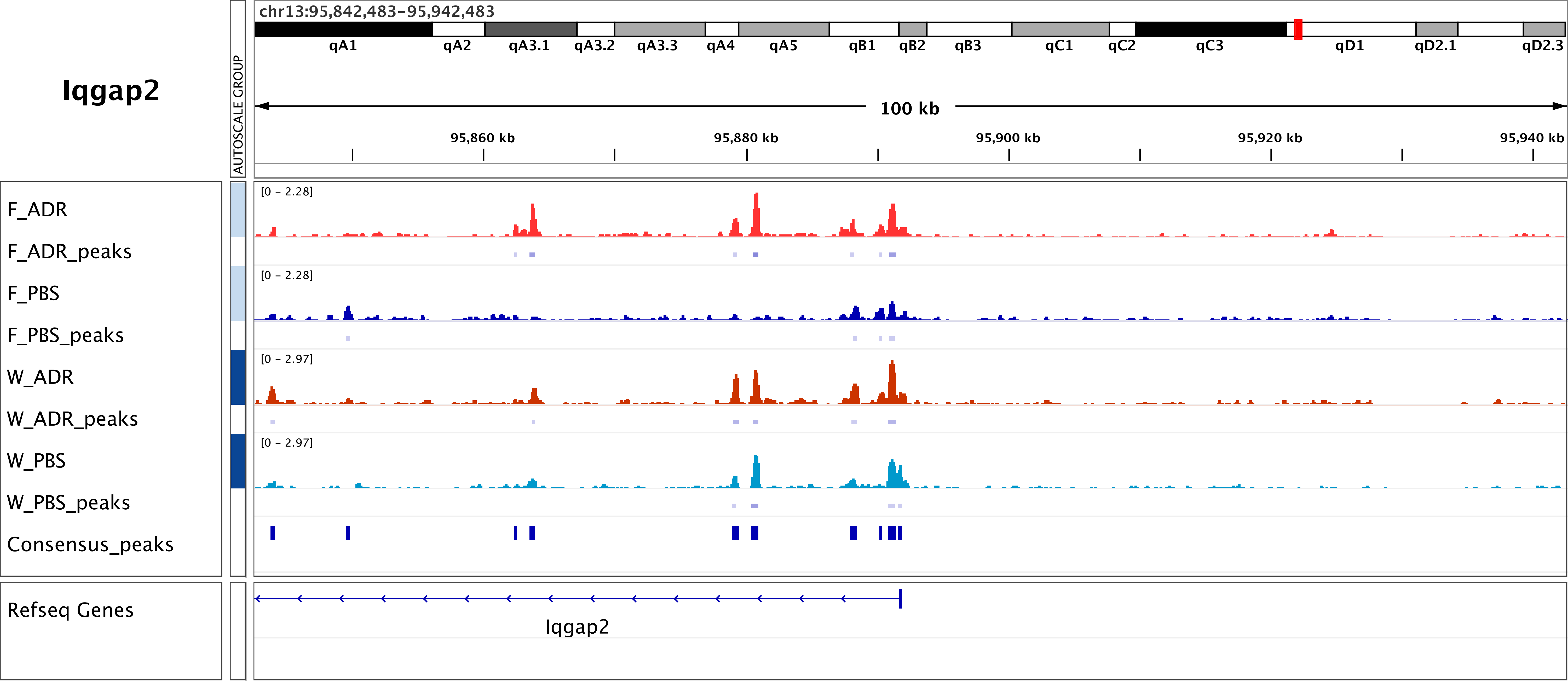
Iqgap2
AUTOSCALE GROUP
chr13:95,842,483-95,942,483
qA1
qA2
qA3.1
qA3.2
qA3.3
qA4
qA5
qB1
qB2
qB3
qC1
qC2
qC3
qD1
qD2.1
qD2.3
100 kb
95,860 kb
95,880 kb
95,900 kb
95,920 kb
95,940 kb
F_ADR
F_ADR_peaks
F_PBS
F_PBS_peaks
W_ADR
W_ADR_peaks
W_PBS
W_PBS_peaks
Consensus_peaks
[0 - 2.28]
[0 - 2.28]
[0 - 2.97]
[0 - 2.97]
Refseq Genes
Iqgap2

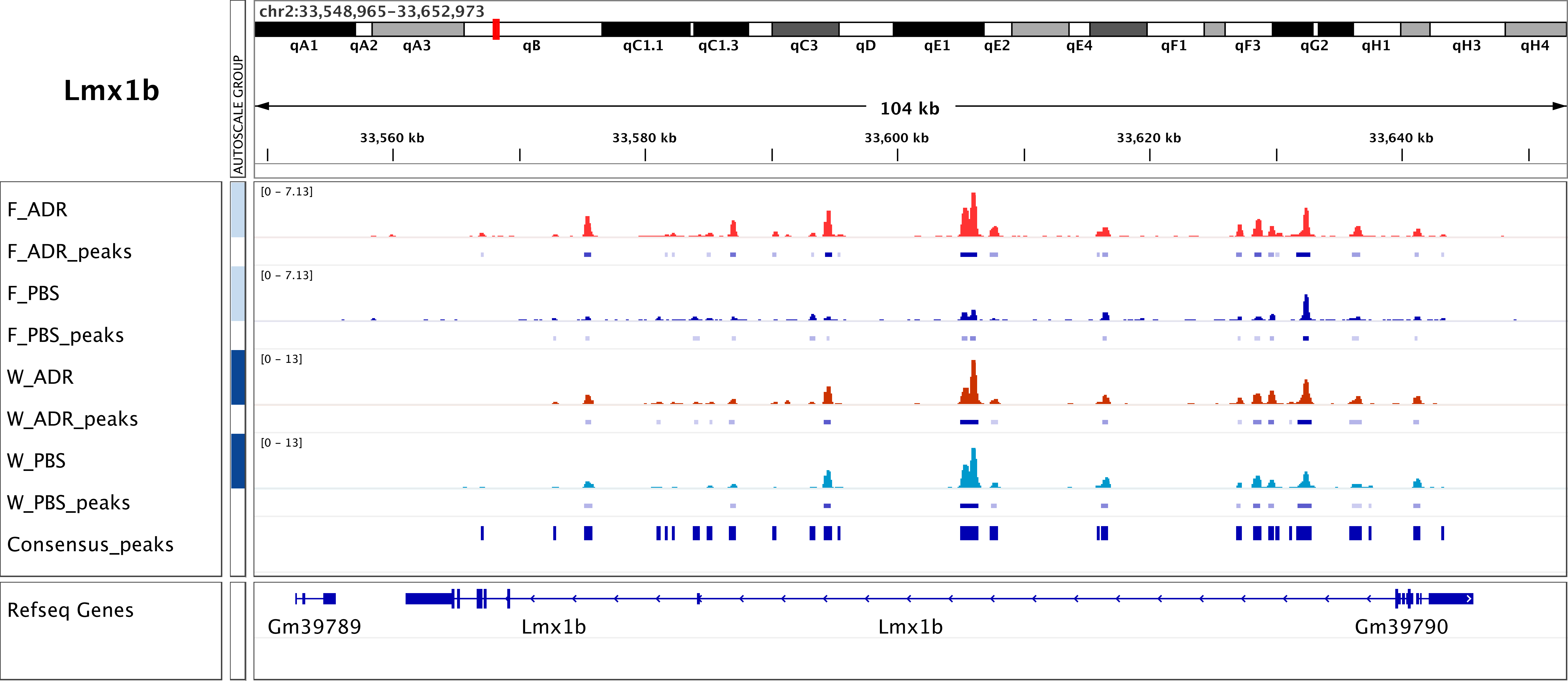

Lmx1b
AUTOSCALE GROUP
chr2:33,548,965-33,652,973
qA1
qA2
qA3
qB
qC1.1
qC1.3
qC3
qD
qE1
qE2
qE4
qF1
qF3
qG2
qH1
qH3
qH4
104 kb
33,560 kb
33,580 kb
33,600 kb
33,620 kb
33,640 kb
F_ADR
[0 - 7.13]
F_ADR_peaks
F_PBS
[0 - 7.13]
F_PBS_peaks
W_ADR
[0 - 13]
W_ADR_peaks
W_PBS
[0 - 13]
W_PBS_peaks
Consensus_peaks
Refseq Genes
Gm39789
Lmx1b
Lmx1b
Gm39790

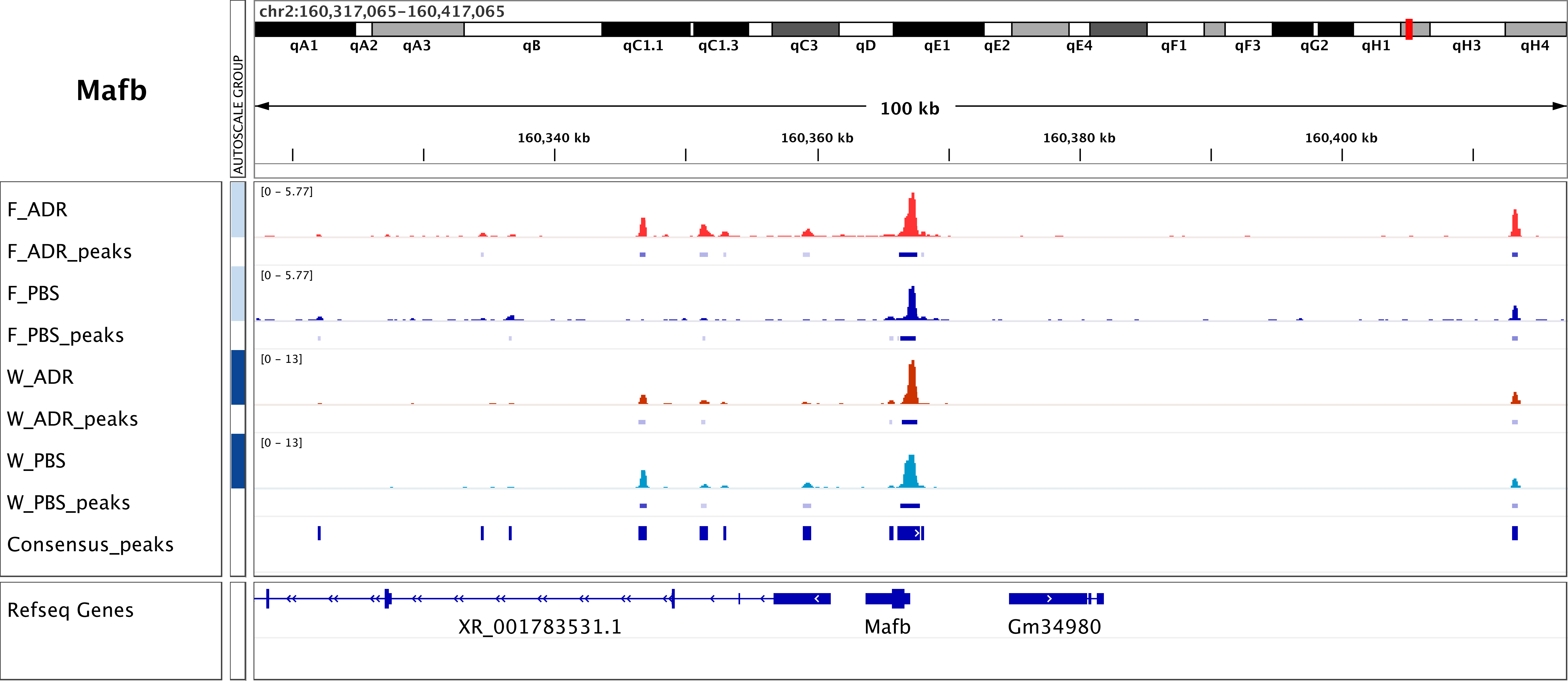

Mafb
AUTOSCALE GROUP
chr2:160,317,065-160,417,065
qA1
qA2
qA3
qB
qC1.1
qC1.3
qC3
qD
qE1
qE2
qE4
qF1
qF3
qG2
qH1
qH3
qH4
100 kb
160,340 kb
160,360 kb
160,380 kb
160,400 kb
F_ADR
[0 - 5.77]
F_ADR_peaks
F_PBS
[0 - 5.77]
F_PBS_peaks
W_ADR
[0 - 13]
W_ADR_peaks
W_PBS
[0 - 13]
W_PBS_peaks
Consensus_peaks
Refseq Genes
XR_001783531.1
Mafb
Gm34980

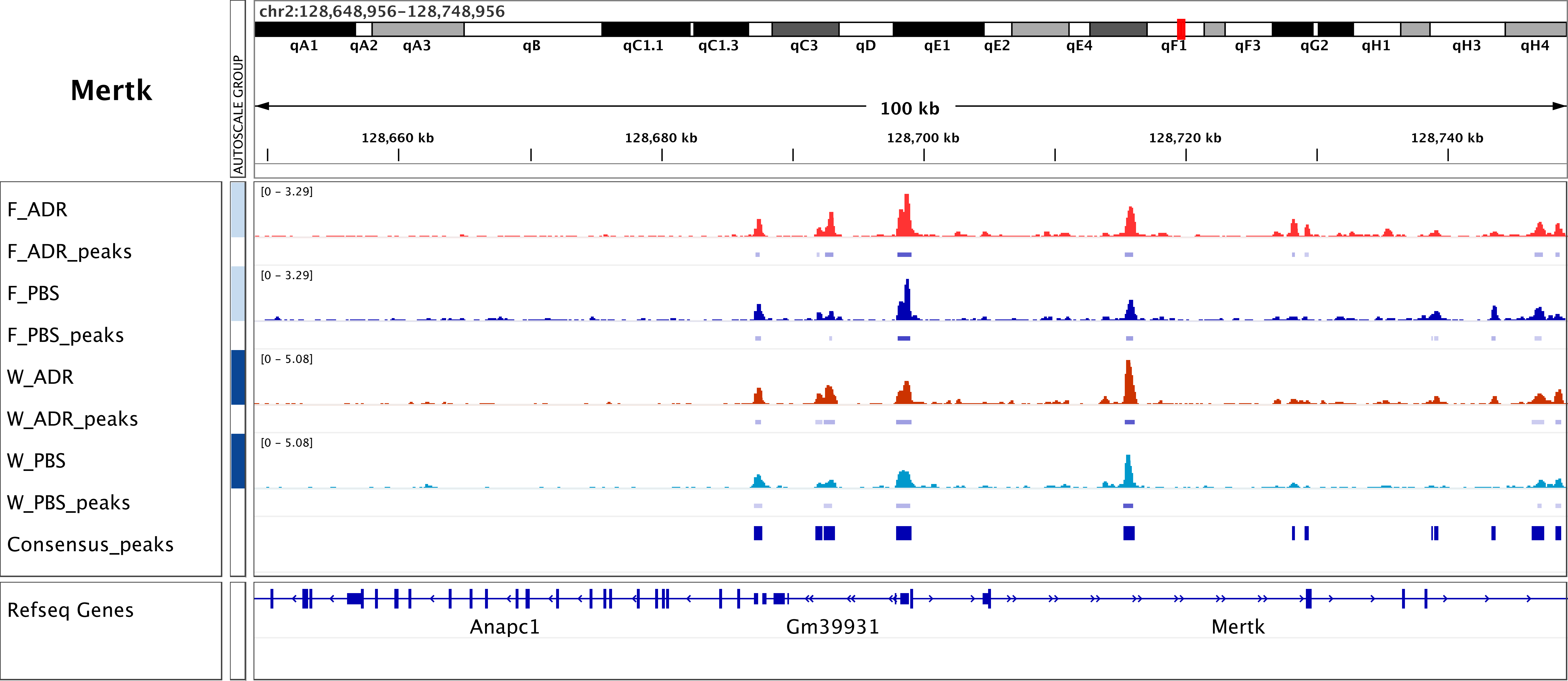
Mertk
AUTOSCALE GROUP
chr2:128,648,956-128,748,956
qA1 qA2 qA3 qB qC1.1 qC1.3 qC3 qD qE1 qE2 qE4 qF1 qF3 qG2 qH1 qH3 qH4
100 kb
128,660 kb
128,680 kb
128,700 kb
128,720 kb
128,740 kb
F_ADR
F_ADR_peaks
F_PBS
F_PBS_peaks
W_ADR
W_ADR_peaks
W_PBS
W_PBS_peaks
Consensus_peaks
[0 - 3.29]
[0 - 3.29]
[0 - 5.08]
[0 - 5.08]
Refseq Genes
Anapc1
Gm39931
Mertk

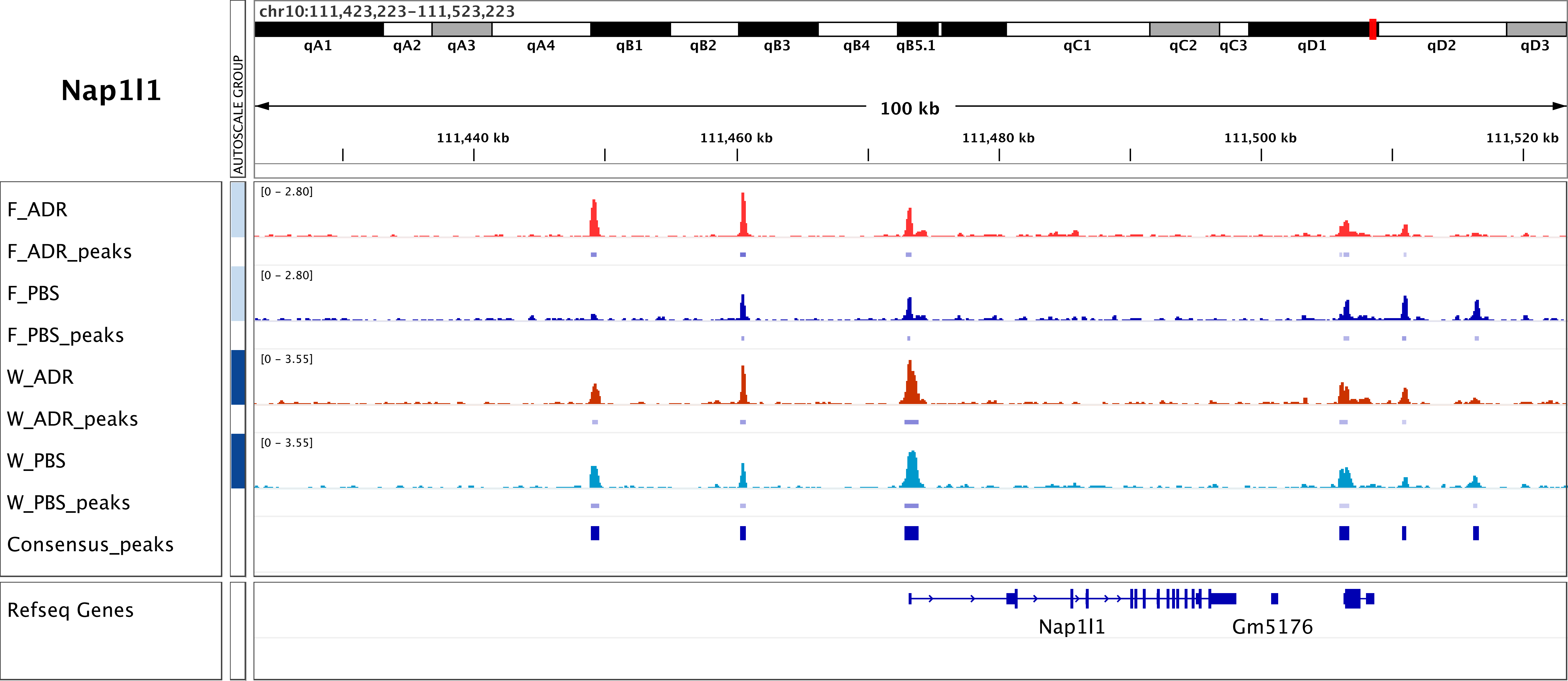
Nap1l1
AUTOSCALE GROUP
chr10:111,423,223-111,523,223
qA1
qA2
qA3
qA4
qB1
qB2
qB3
qB4
qB5.1
qC1
qC2
qC3
qD1
qD2
qD3
100 kb
111,440 kb
111,460 kb
111,480 kb
111,500 kb
111,520 kb
F_ADR
[0 - 2.80]
F_ADR_peaks
F_PBS
[0 - 2.80]
F_PBS_peaks
W_ADR
[0 - 3.55]
W_ADR_peaks
W_PBS
[0 - 3.55]
W_PBS_peaks
Consensus_peaks
Refseq Genes
Nap1l1
Gm5176

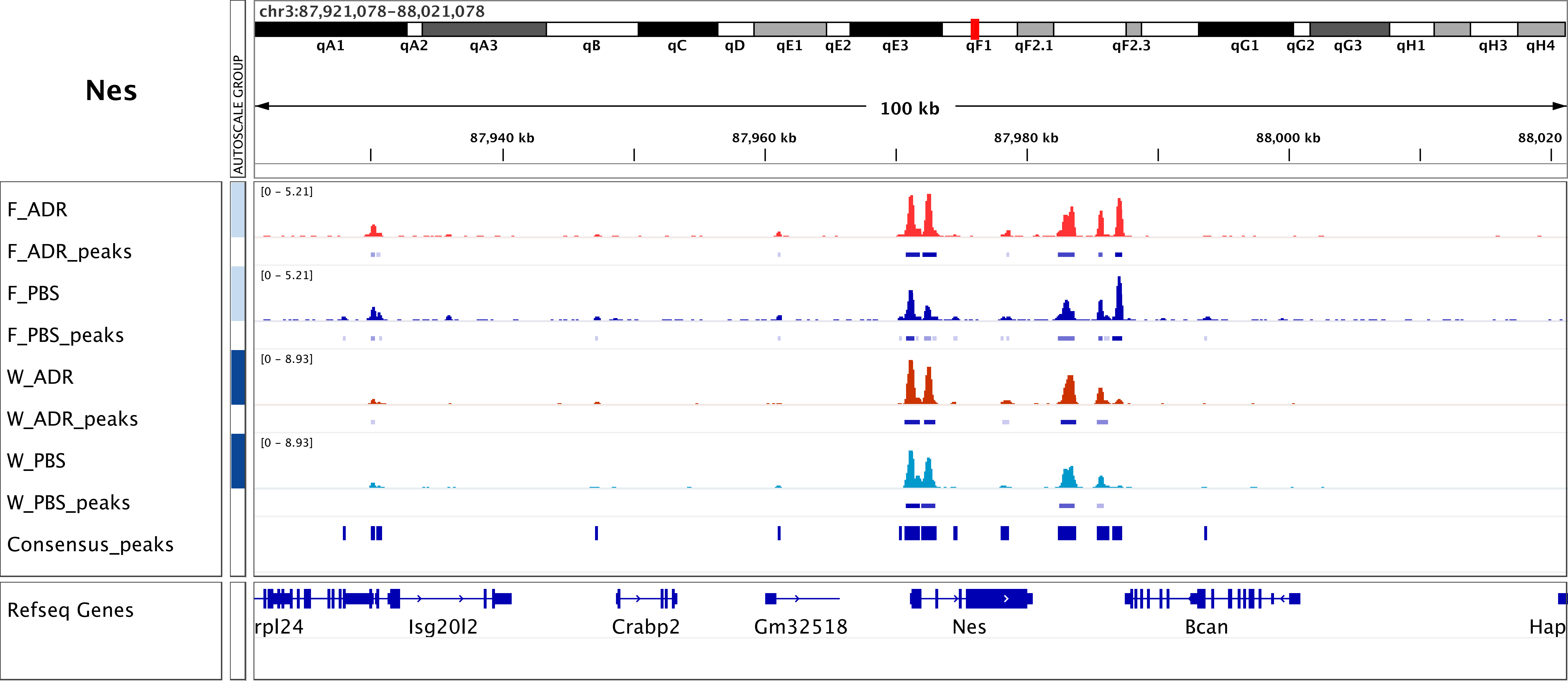
Nes
AUTOSCALE GROUP
chr3:87,921,078-88,021,078
qA1
qA2
qA3
qB
qC
qD
qE1
qE2
qE3
qF1
qF2.1
qF2.3
qG1
qG2
qG3
qH1
qH3
qH4
100 kb
87,940 kb
87,960 kb
87,980 kb
88,000 kb
88,020
F_ADR
F_ADR_peaks
F_PBS
F_PBS_peaks
W_ADR
W_ADR_peaks
W_PBS
W_PBS_peaks
Consensus_peaks
[0 - 5.21]
[0 - 5.21]
[0 - 8.93]
[0 - 8.93]
Refseq Genes
rpl24
Isg20l2
Crabp2
Gm32518
Nes
Bcan
Hap

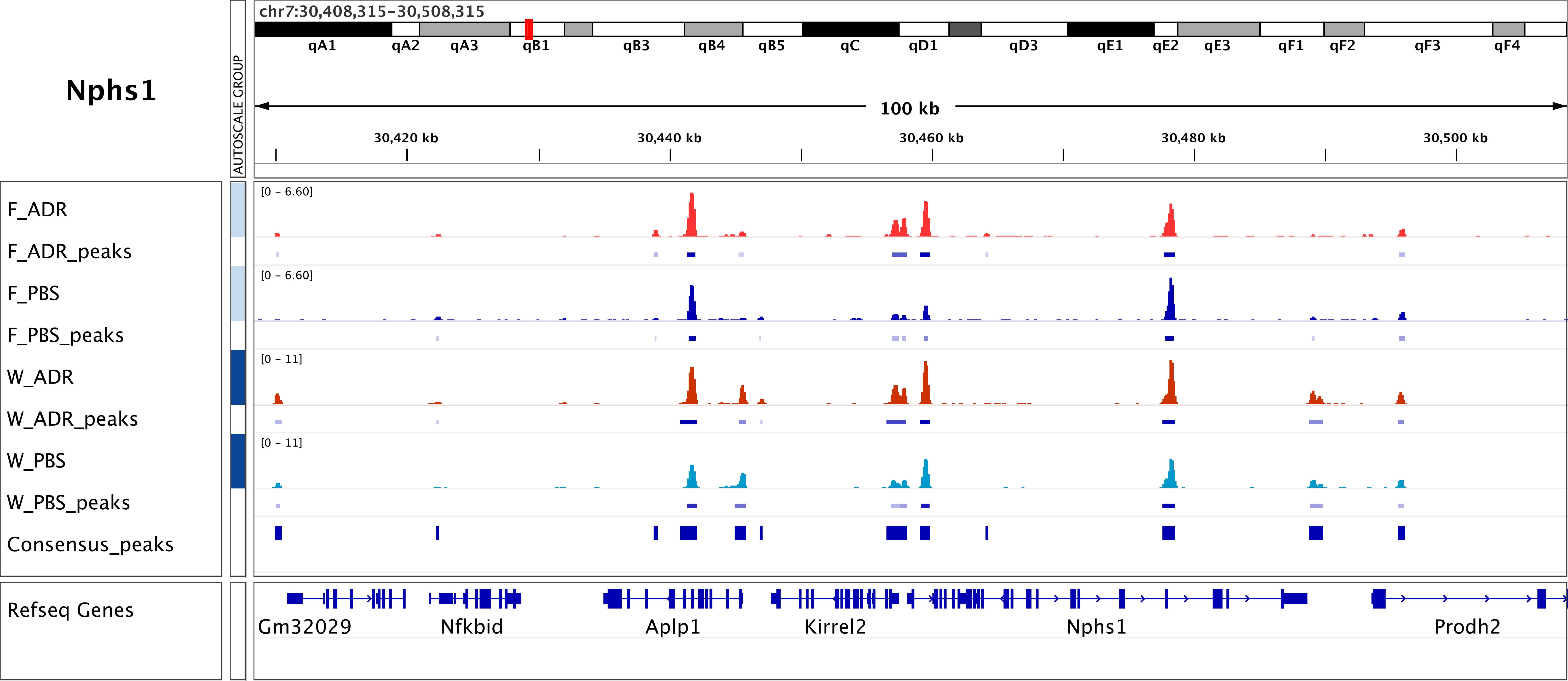

Nphs1
AUTOSCALE GROUP
chr7:30,408,315-30,508,315
qA1
qA2
qA3
qB1
qB3
qB4
qB5
qC
qD1
qD3
qE1
qE2
qE3
qF1
qF2
qF3
qF4
100 kb
30,420 kb
30,440 kb
30,460 kb
30,480 kb
30,500 kb
F_ADR
[0 - 6.60]
F_ADR_peaks
F_PBS
[0 - 6.60]
F_PBS_peaks
W_ADR
[0 - 11]
W_ADR_peaks
W_PBS
[0 - 11]
W_PBS_peaks
Consensus_peaks
Refseq Genes
Gm32029
Nfkbid
Aplp1
Kirrel2
Nphs1
Prodh2

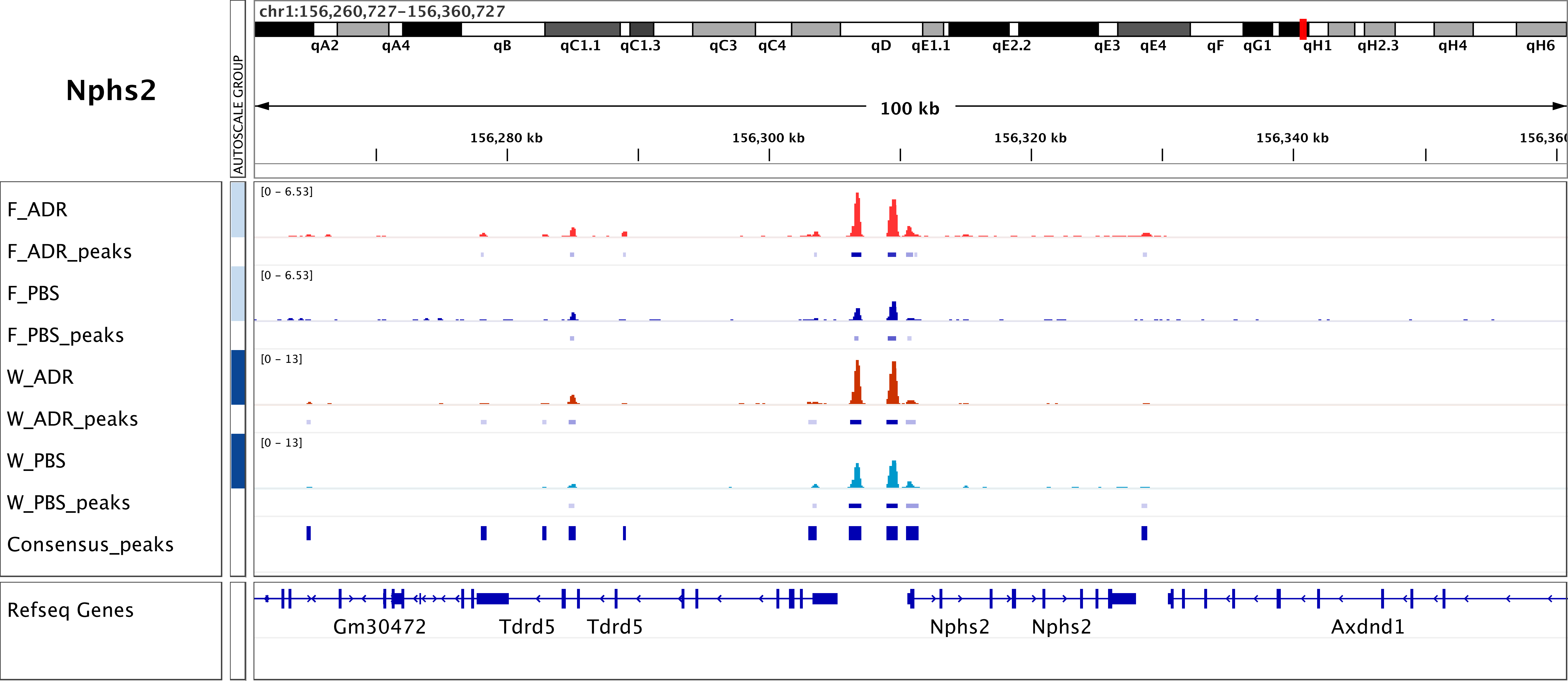

Nphs2
AUTOSCALE GROUP
chr1:156,260,727–156,360,727
qA2
qA4
qB
qC1.1
qC1.3
qC3
qC4
qD
qE1.1
qE2.2
qE3
qE4
qF
qG1
qH1
qH2.3
qH4
qH6
100 kb
156,280 kb
156,300 kb
156,320 kb
156,340 kb
F_ADR
F_ADR_peaks
F_PBS
F_PBS_peaks
W_ADR
W_ADR_peaks
W_PBS
W_PBS_peaks
Consensus_peaks
[0 – 6.53]
[0 – 6.53]
[0 – 13]
[0 – 13]
Refseq Genes
Gm30472
Tdrd5
Tdrd5
Nphs2
Nphs2
Axdnd1

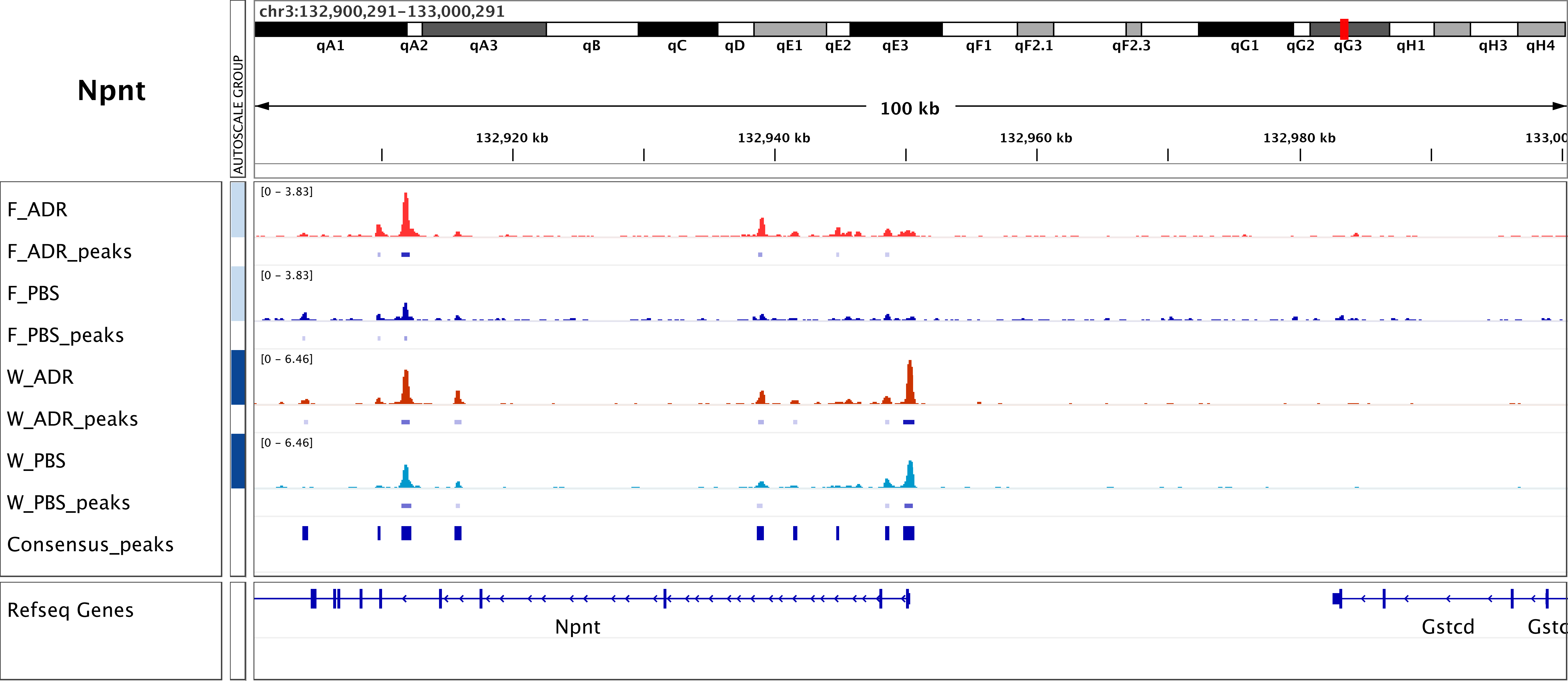
Npnt
AUTOSCALE GROUP
chr3:132,900,291-133,000,291
qA1
qA2
qA3
qB
qC
qD
qE1
qE2
qE3
qF1
qF2.1
qF2.3
qG1
qG2
qG3
qH1
qH3
qH4
100 kb
132,920 kb
132,940 kb
132,960 kb
132,980 kb
F_ADR
[0 - 3.83]
F_ADR_peaks
F_PBS
[0 - 3.83]
F_PBS_peaks
W_ADR
[0 - 6.46]
W_ADR_peaks
W_PBS
[0 - 6.46]
W_PBS_peaks
Consensus_peaks
Refseq Genes
Npnt
Gstcd

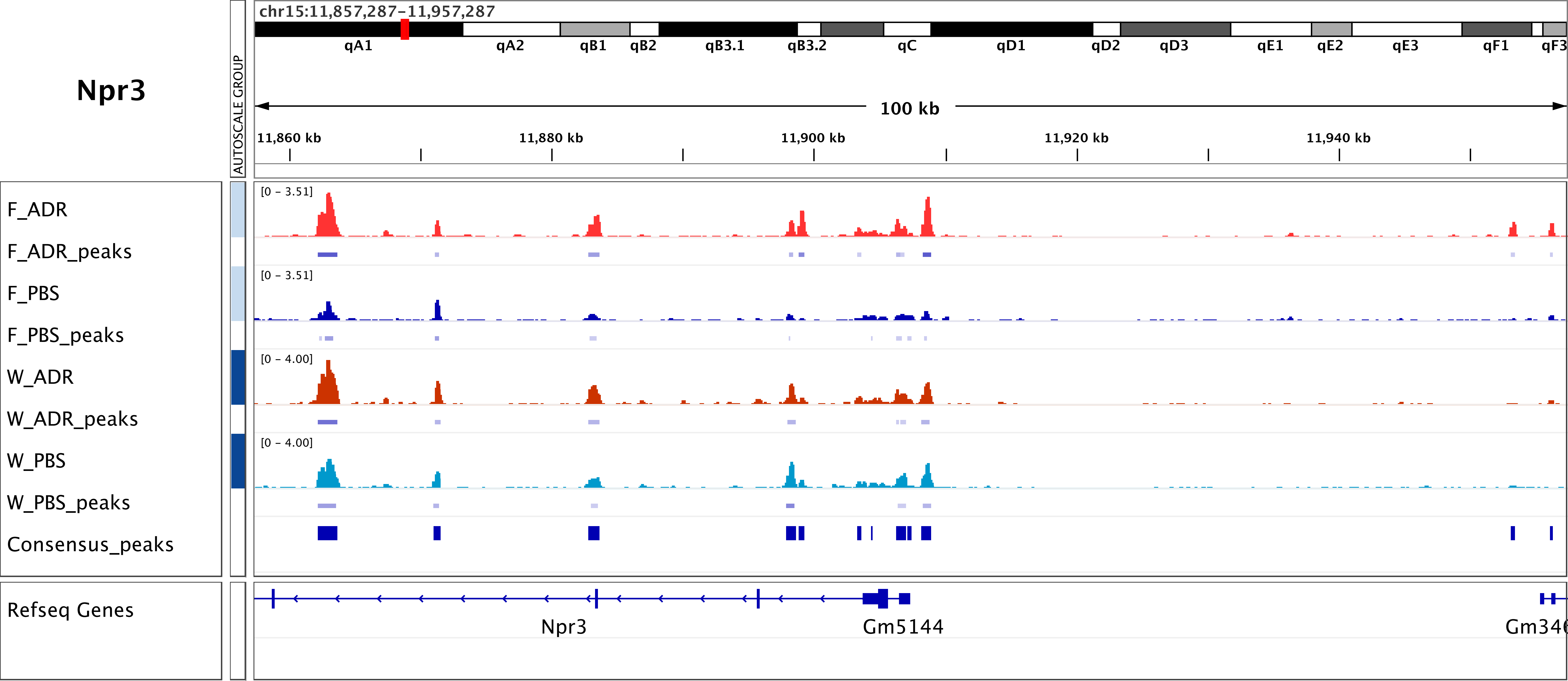

Npr3
AUTOSCALE GROUP
chr15:11,857,287-11,957,287
qA1
qA2
qB1
qB2
qB3.1
qB3.2
qC
qD1
qD2
qD3
qE1
qE2
qE3
qF1
qF3
100 kb
11,860 kb
11,880 kb
11,900 kb
11,920 kb
11,940 kb
F_ADR
F_ADR_peaks
F_PBS
F_PBS_peaks
W_ADR
W_ADR_peaks
W_PBS
W_PBS_peaks
Consensus_peaks
[0 - 3.51]
[0 - 3.51]
[0 - 4.00]
[0 - 4.00]
Refseq Genes
Npr3
Gm5144

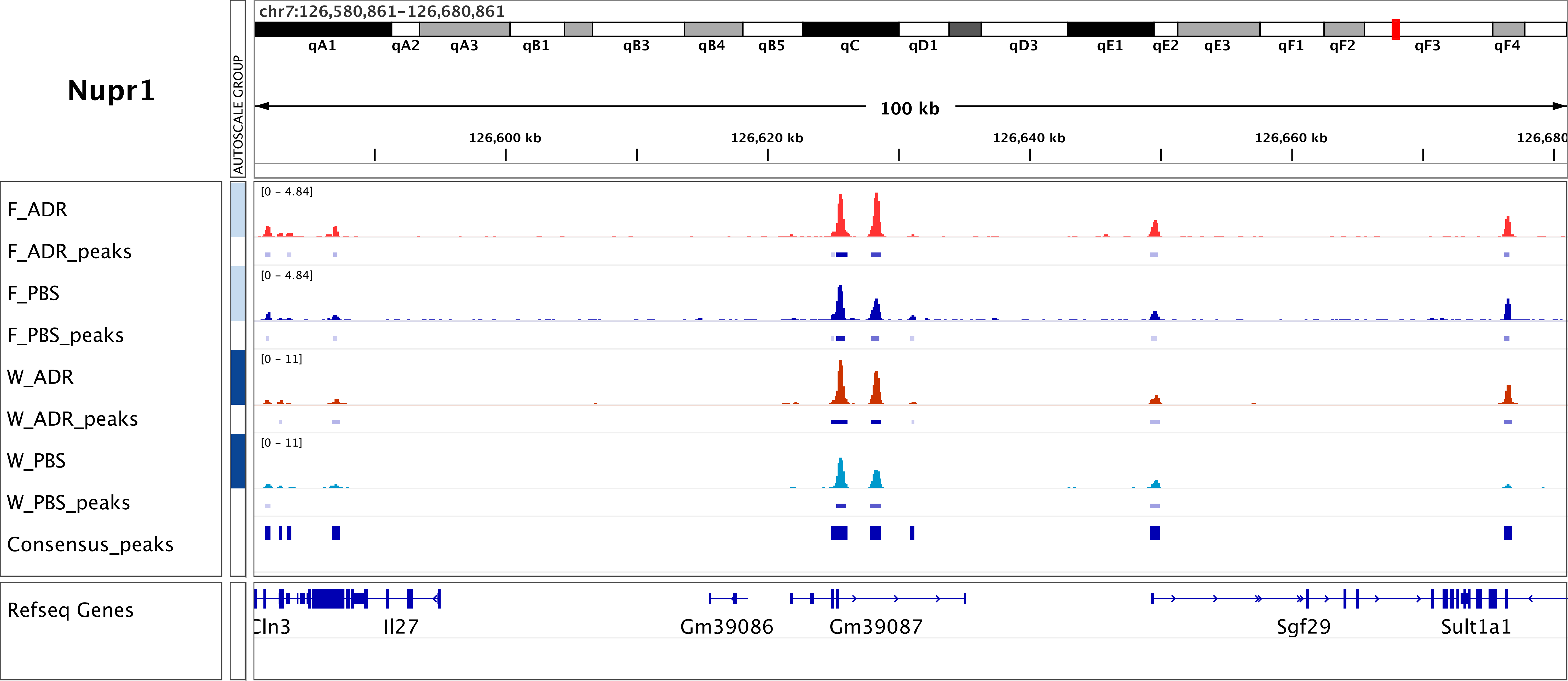
Nupr1
chr7:126,580,861-126,680,861
qA1 qA2 qA3 qB1 qB3 qB4 qB5 qC qD1 qD3 qE1 qE2 qE3 qF1 qF2 qF3 qF4
100 kb
126,600 kb
126,620 kb
126,640 kb
126,660 kb
126,680
AUTOSCALE GROUP
F_ADR
F_ADR_peaks
F_PBS
F_PBS_peaks
W_ADR
W_ADR_peaks
W_PBS
W_PBS_peaks
Consensus_peaks
[0 - 4.84]
[0 - 4.84]
[0 - 11]
[0 - 11]
Refseq Genes
Cln3
Il27
Gm39086
Gm39087
Sgf29
Sult1a1

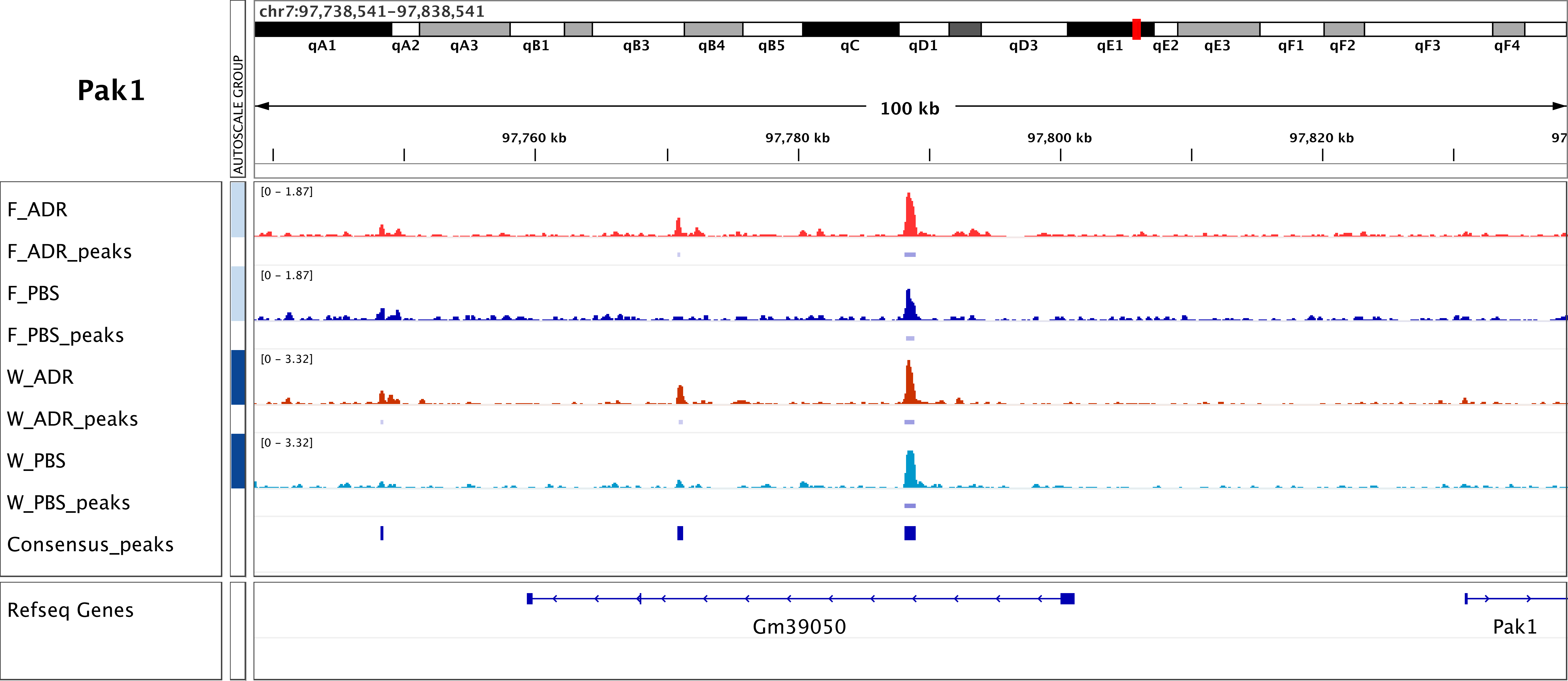
Pak1
AUTOSCALE GROUP
chr7:97,738,541-97,838,541
qA1
qA2
qA3
qB1
qB3
qB4
qB5
qC
qD1
qD3
qE1
qE2
qE3
qF1
qF2
qF3
qF4
100 kb
97,760 kb
97,780 kb
97,800 kb
97,820 kb
F_ADR
F_ADR_peaks
F_PBS
F_PBS_peaks
W_ADR
W_ADR_peaks
W_PBS
W_PBS_peaks
Consensus_peaks
[0 - 1.87]
[0 - 1.87]
[0 - 3.32]
[0 - 3.32]
Refseq Genes
Gm39050
Pak1

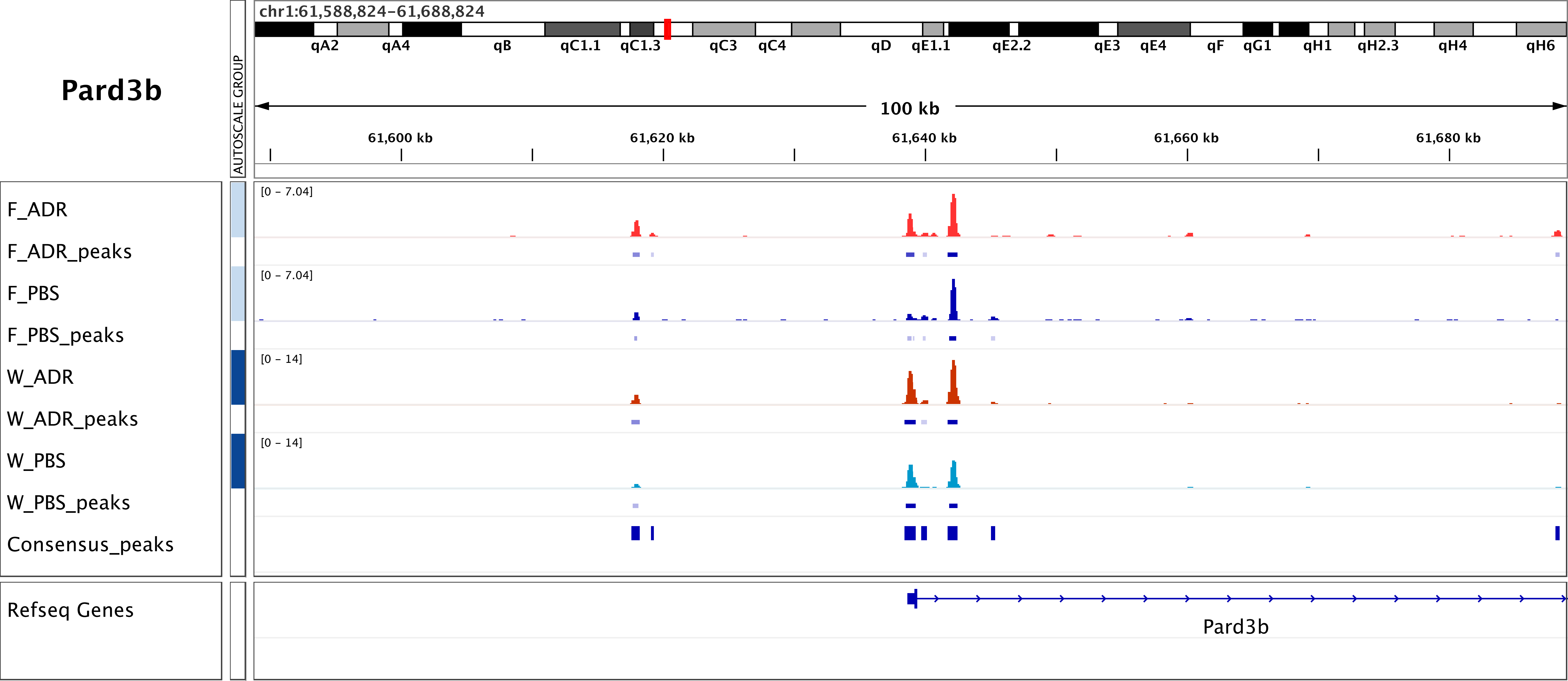

Pard3b
AUTOSCALE GROUP
chr1:61,588,824-61,688,824
qA2
qA4
qB
qC1.1
qC1.3
qC3
qC4
qD
qE1.1
qE2.2
qE3
qE4
qF
qG1
qH1
qH2.3
qH4
qH6
100 kb
61,600 kb
61,620 kb
61,640 kb
61,660 kb
61,680 kb
F_ADR
F_ADR_peaks
F_PBS
F_PBS_peaks
W_ADR
W_ADR_peaks
W_PBS
W_PBS_peaks
Consensus_peaks
[0 - 7.04]
[0 - 7.04]
[0 - 14]
[0 - 14]
Refseq Genes
Pard3b

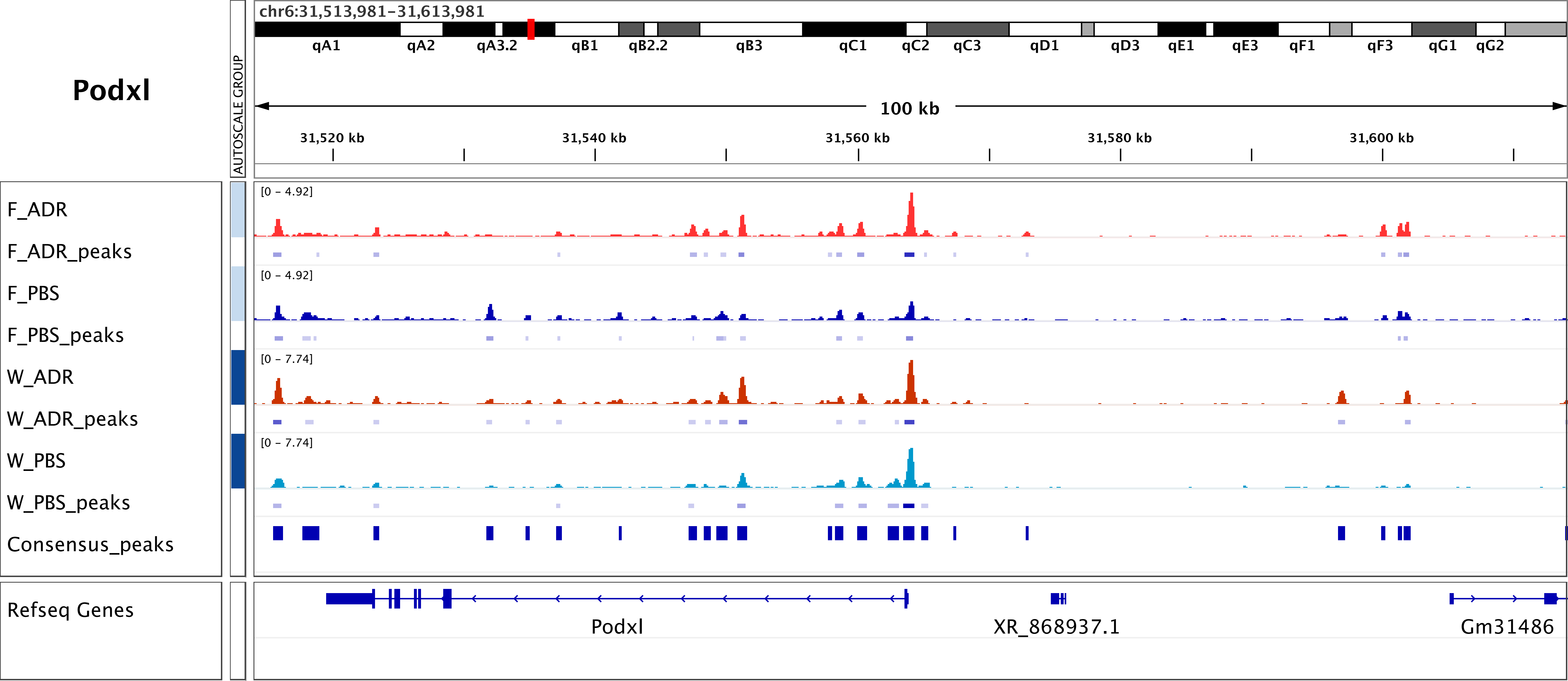
Podxl
AUTOSCALE GROUP
chr6:31,513,981-31,613,981
qA1
qA2
qA3.2
qB1
qB2.2
qB3
qC1
qC2
qC3
qD1
qD3
qE1
qE3
qF1
qF3
qG1
qG2
100 kb
31,520 kb
31,540 kb
31,560 kb
31,580 kb
31,600 kb
F_ADR
F_ADR_peaks
F_PBS
F_PBS_peaks
W_ADR
W_ADR_peaks
W_PBS
W_PBS_peaks
Consensus_peaks
[0 - 4.92]
[0 - 4.92]
[0 - 7.74]
[0 - 7.74]
Refseq Genes
Podxl
XR_868937.1
Gm31486

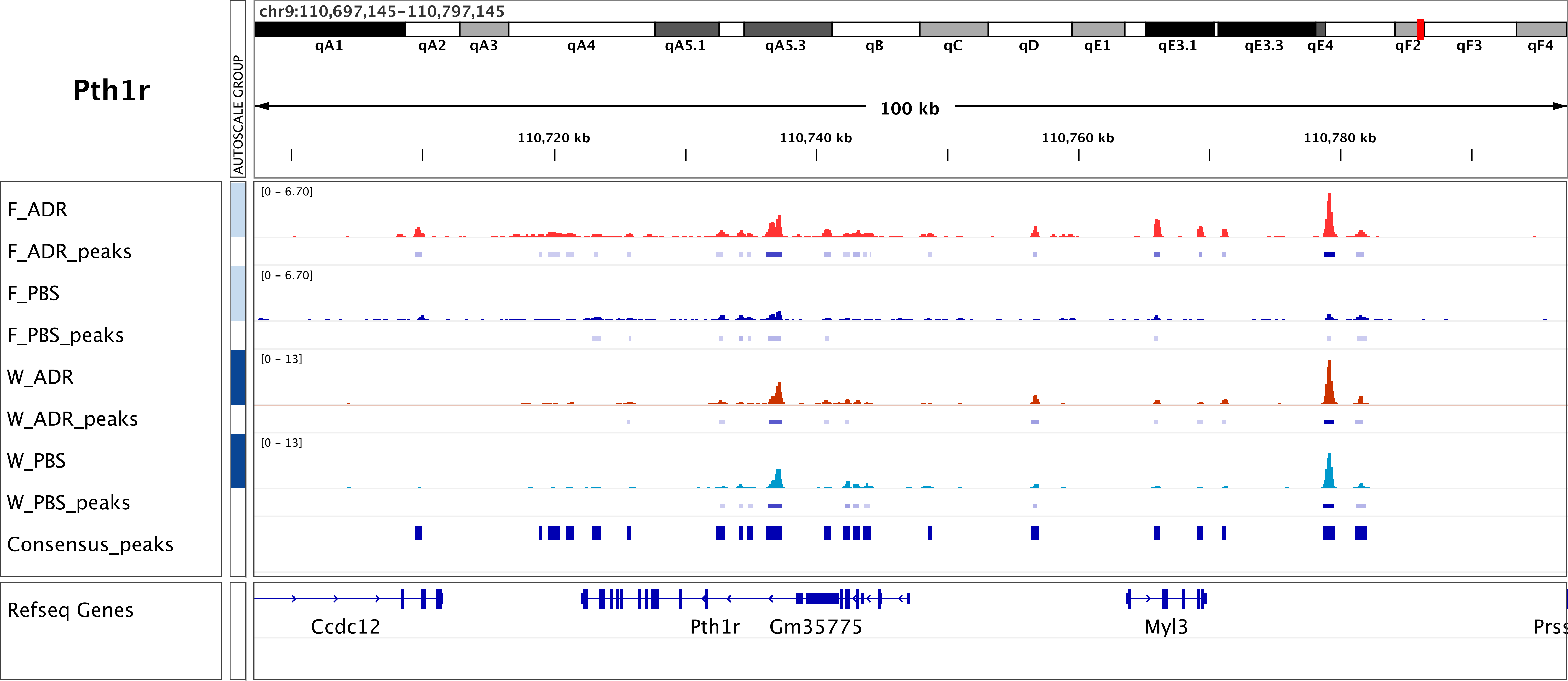

Pth1r
AUTOSCALE GROUP
chr9:110,697,145-110,797,145
qA1
qA2
qA3
qA4
qA5.1
qA5.3
qB
qC
qD
qE1
qE3.1
qE3.3
qE4
qF2
qF3
qF4
100 kb
110,720 kb
110,740 kb
110,760 kb
110,780 kb
F_ADR
[0 - 6.70]
F_ADR_peaks
F_PBS
[0 - 6.70]
F_PBS_peaks
W_ADR
[0 - 13]
W_ADR_peaks
W_PBS
[0 - 13]
W_PBS_peaks
Consensus_peaks
Refseq Genes
Ccdc12
Pth1r
Gm35775
Myl3

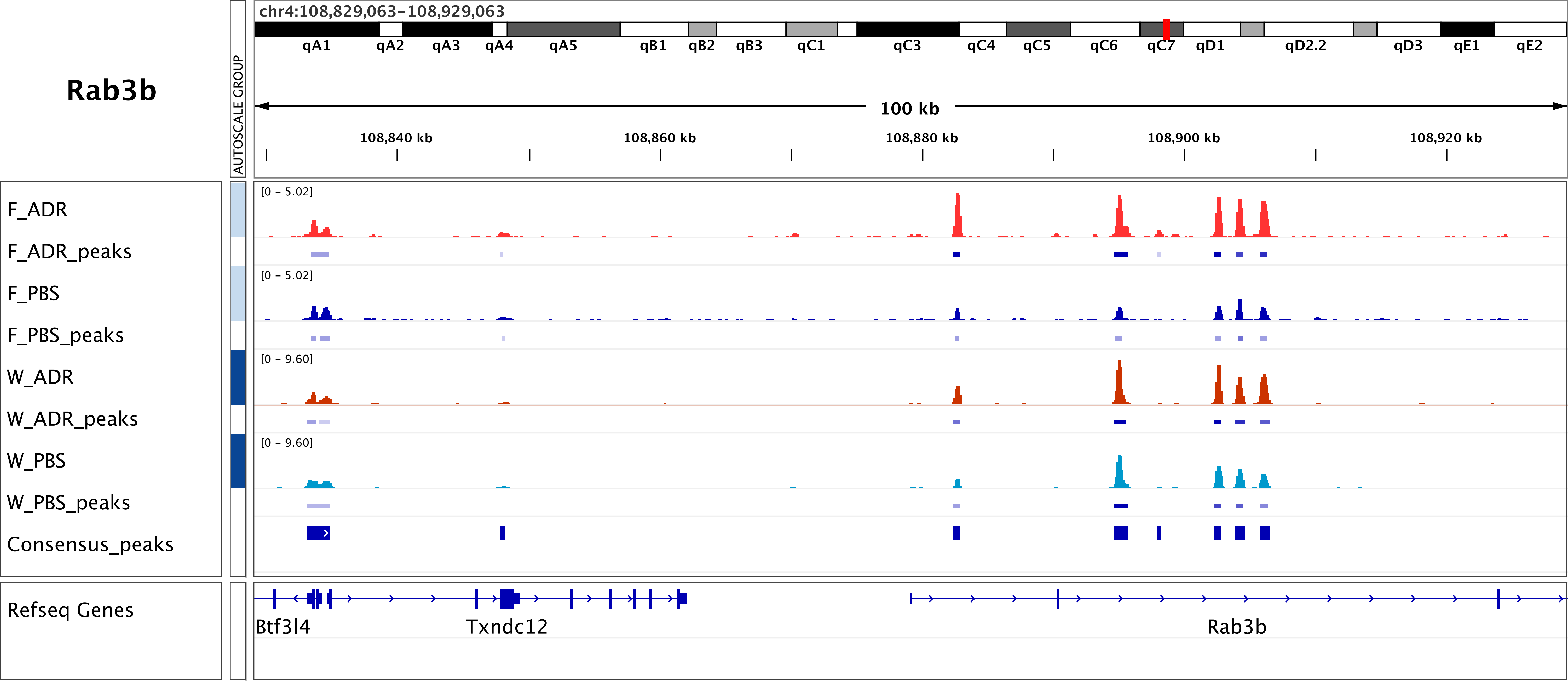
Rab3b
AUTOSCALE GROUP
chr4:108,829,063-108,929,063
qA1
qA2
qA3
qA4
qA5
qB1
qB2
qB3
qC1
qC3
qC4
qC5
qC6
qC7
qD1
qD2.2
qD3
qE1
qE2
100 kb
108,840 kb
108,860 kb
108,880 kb
108,900 kb
108,920 kb
F_ADR
F_ADR_peaks
F_PBS
F_PBS_peaks
W_ADR
W_ADR_peaks
W_PBS
W_PBS_peaks
Consensus_peaks
[0 - 5.02]
[0 - 5.02]
[0 - 9.60]
[0 - 9.60]
Refseq Genes
Btf3l4
Txndc12
Rab3b

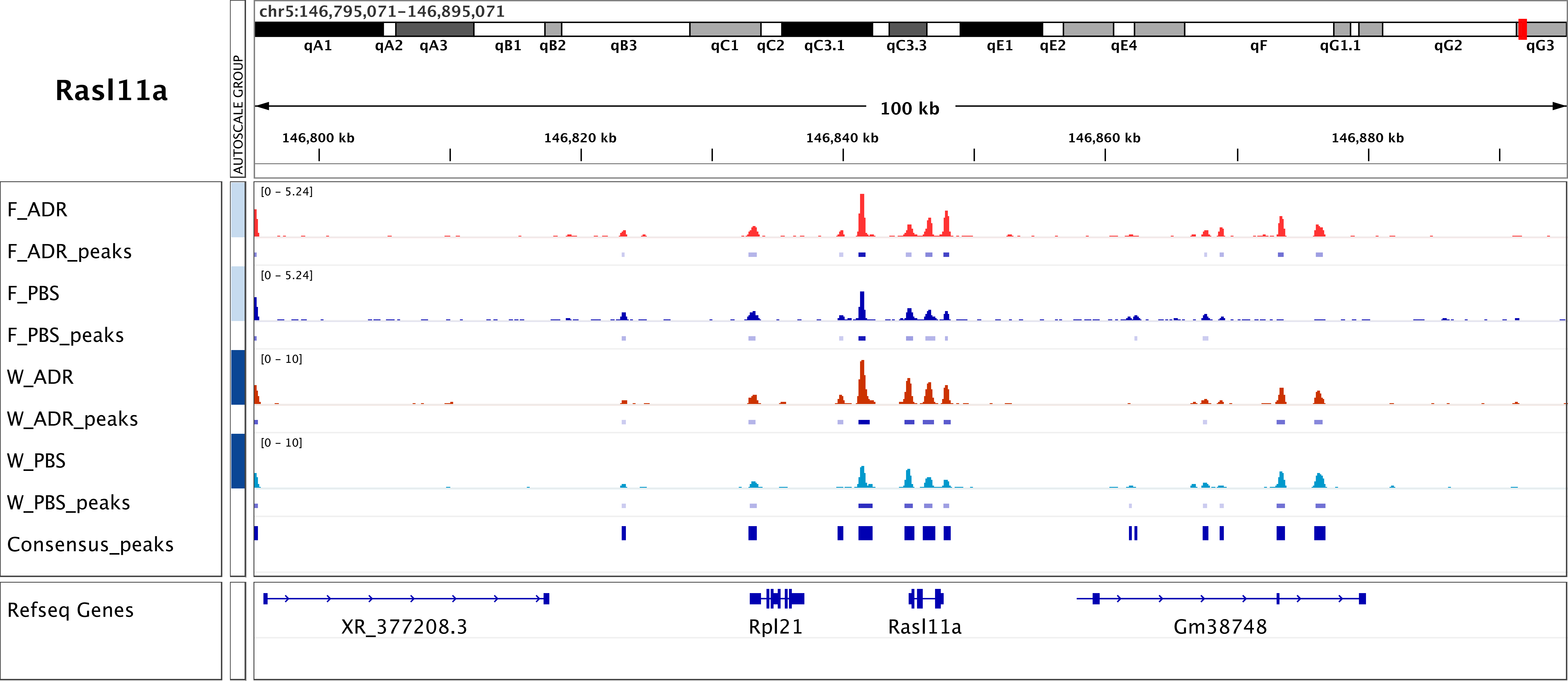
Rasl11a
AUTOSCALE GROUP
chr5:146,795,071–146,895,071
qA1
qA2
qA3
qB1
qB2
qB3
qC1
qC2
qC3.1
qC3.3
qE1
qE2
qE4
qF
qG1.1
qG2
qG3
100 kb
146,800 kb
146,820 kb
146,840 kb
146,860 kb
146,880 kb
F_ADR
[0 – 5.24]
F_ADR_peaks
F_PBS
[0 – 5.24]
F_PBS_peaks
W_ADR
[0 – 10]
W_ADR_peaks
W_PBS
[0 – 10]
W_PBS_peaks
Consensus_peaks
Refseq Genes
XR_377208.3
Rpl21
Rasl11a
Gm38748

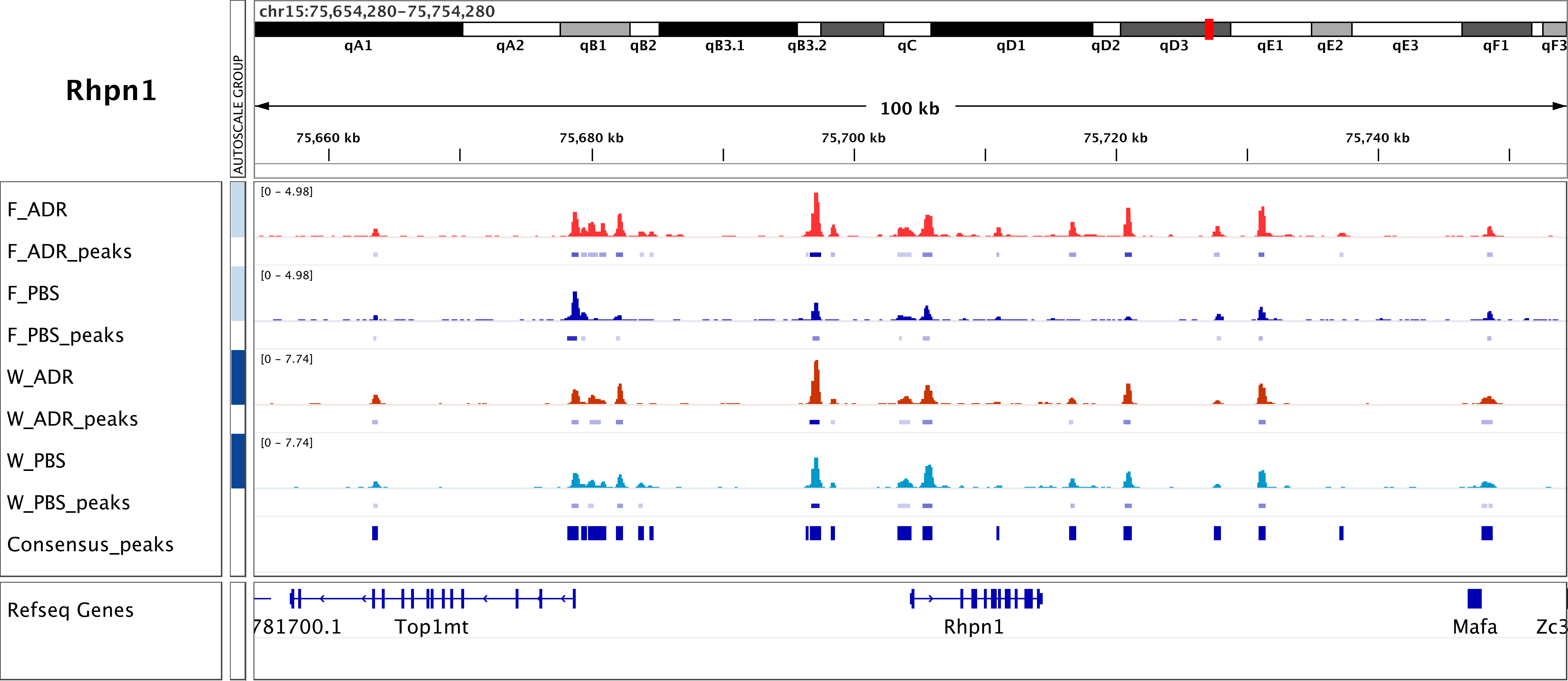

Rhpn1
AUTOSCALE GROUP
chr15:75,654,280-75,754,280
qA1
qA2
qB1
qB2
qB3.1
qB3.2
qC
qD1
qD2
qD3
qE1
qE2
qE3
qF1
qF3
100 kb
75,660 kb
75,680 kb
75,700 kb
75,720 kb
75,740 kb
F_ADR
F_ADR_peaks
F_PBS
F_PBS_peaks
W_ADR
W_ADR_peaks
W_PBS
W_PBS_peaks
Consensus_peaks
[0 - 4.98]
[0 - 4.98]
[0 - 7.74]
[0 - 7.74]
Refseq Genes
781700.1
Top1mt
Rhpn1
Mafa
Zc3

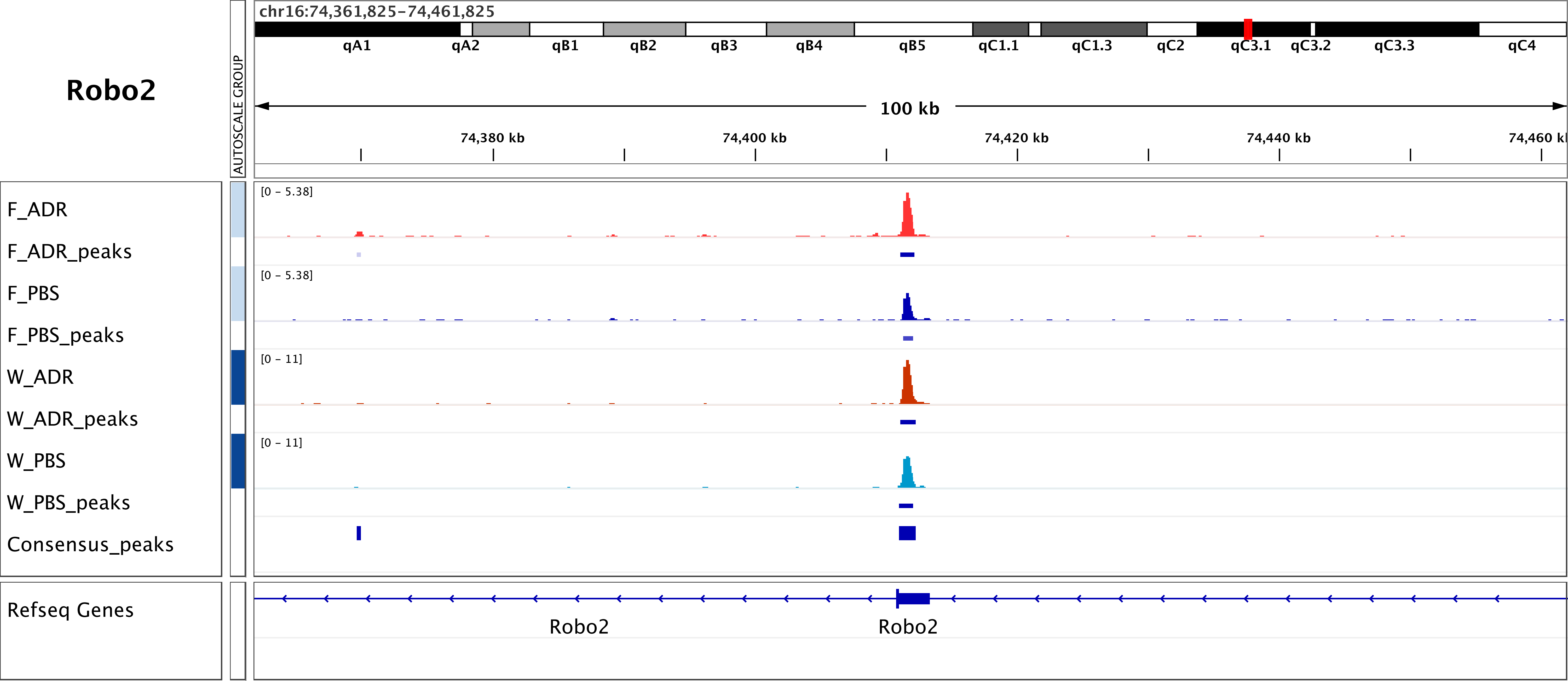
Robo2
AUTOSCALE GROUP
chr16:74,361,825-74,461,825
qA1
qA2
qB1
qB2
qB3
qB4
qB5
qC1.1
qC1.3
qC2
qC3.1
qC3.2
qC3.3
qC4
100 kb
74,380 kb
74,400 kb
74,420 kb
74,440 kb
F_ADR
[0 - 5.38]
F_ADR_peaks
F_PBS
[0 - 5.38]
F_PBS_peaks
W_ADR
[0 - 11]
W_ADR_peaks
W_PBS
[0 - 11]
W_PBS_peaks
Consensus_peaks
Refseq Genes
Robo2
Robo2

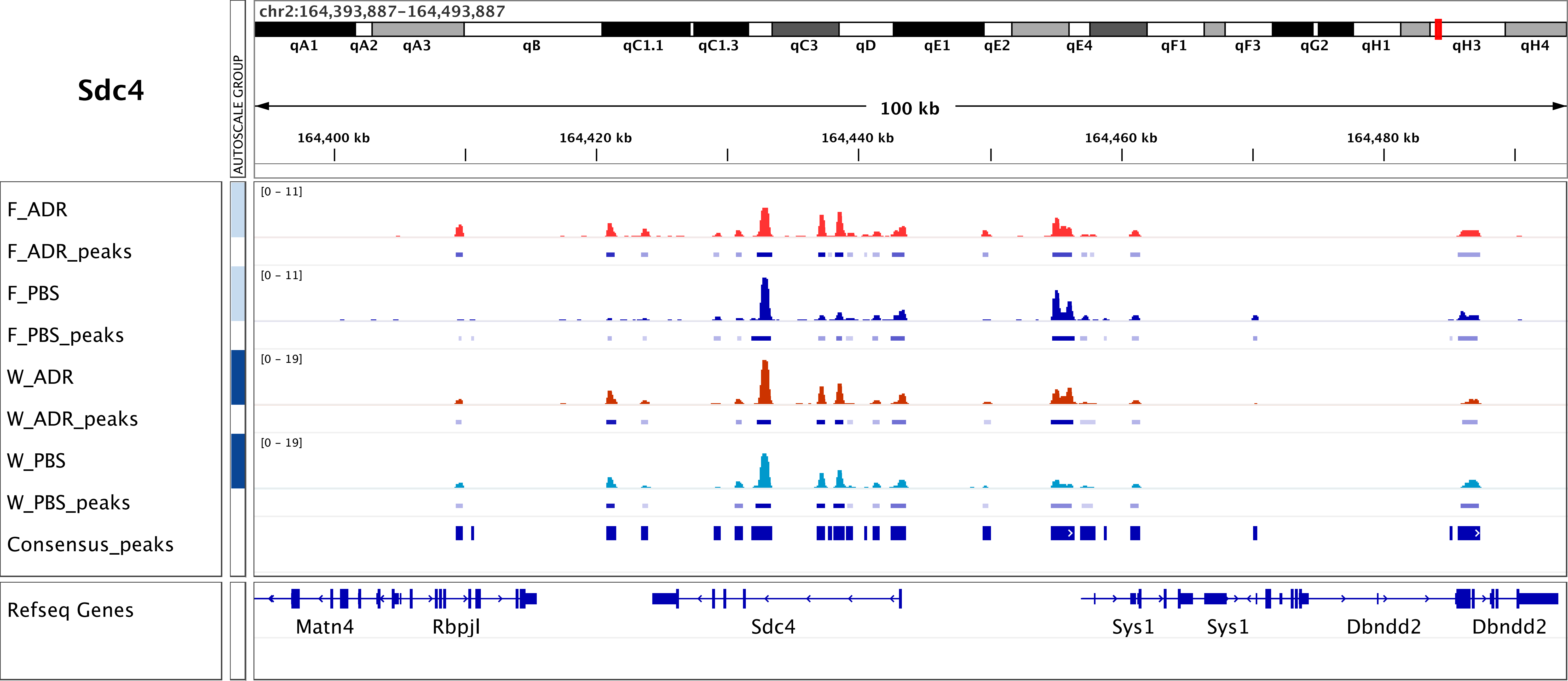

Sdc4
AUTOSCALE GROUP
chr2:164,393,887-164,493,887
qA1
qA2
qA3
qB
qC1.1
qC1.3
qC3
qD
qE1
qE2
qE4
qF1
qF3
qG2
qH1
qH3
qH4
100 kb
164,400 kb
164,420 kb
164,440 kb
164,460 kb
164,480 kb
F_ADR
F_ADR_peaks
F_PBS
F_PBS_peaks
W_ADR
W_ADR_peaks
W_PBS
W_PBS_peaks
Consensus_peaks
[0 - 11]
[0 - 11]
[0 - 19]
[0 - 19]
Refseq Genes
Matn4
Rbpjl
Sdc4
Sys1
Sys1
Dbndd2
Dbndd2

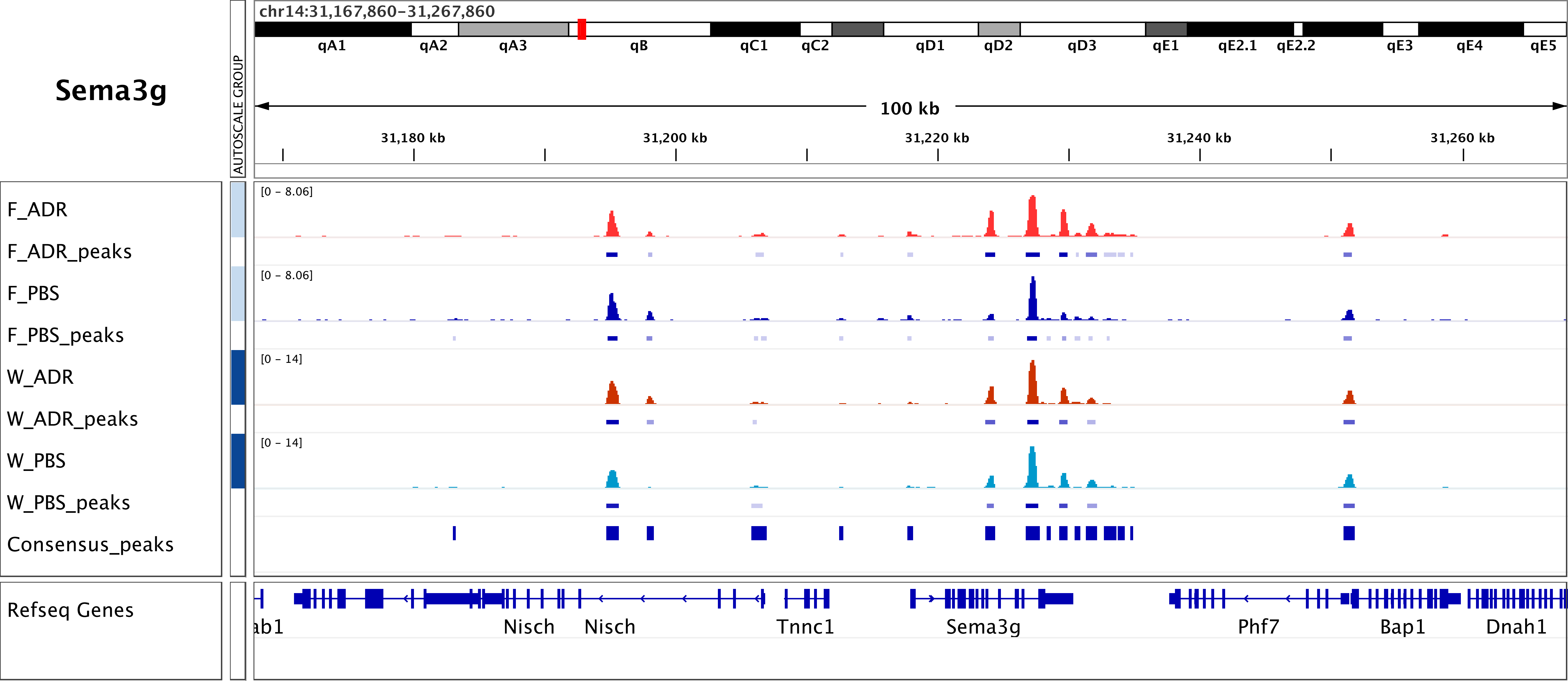

Sema3g
AUTOSCALE GROUP
chr14:31,167,860-31,267,860
qA1
qA2
qA3
qB
qC1
qC2
qD1
qD2
qD3
qE1
qE2.1
qE2.2
qE3
qE4
qE5
100 kb
31,180 kb
31,200 kb
31,220 kb
31,240 kb
31,260 kb
F_ADR
F_ADR_peaks
F_PBS
F_PBS_peaks
W_ADR
W_ADR_peaks
W_PBS
W_PBS_peaks
Consensus_peaks
[0 - 8.06]
[0 - 8.06]
[0 - 14]
[0 - 14]
Refseq Genes
ab1
Nisch
Nisch
Tnnc1
Sema3g
Phf7
Bap1
Dnah1

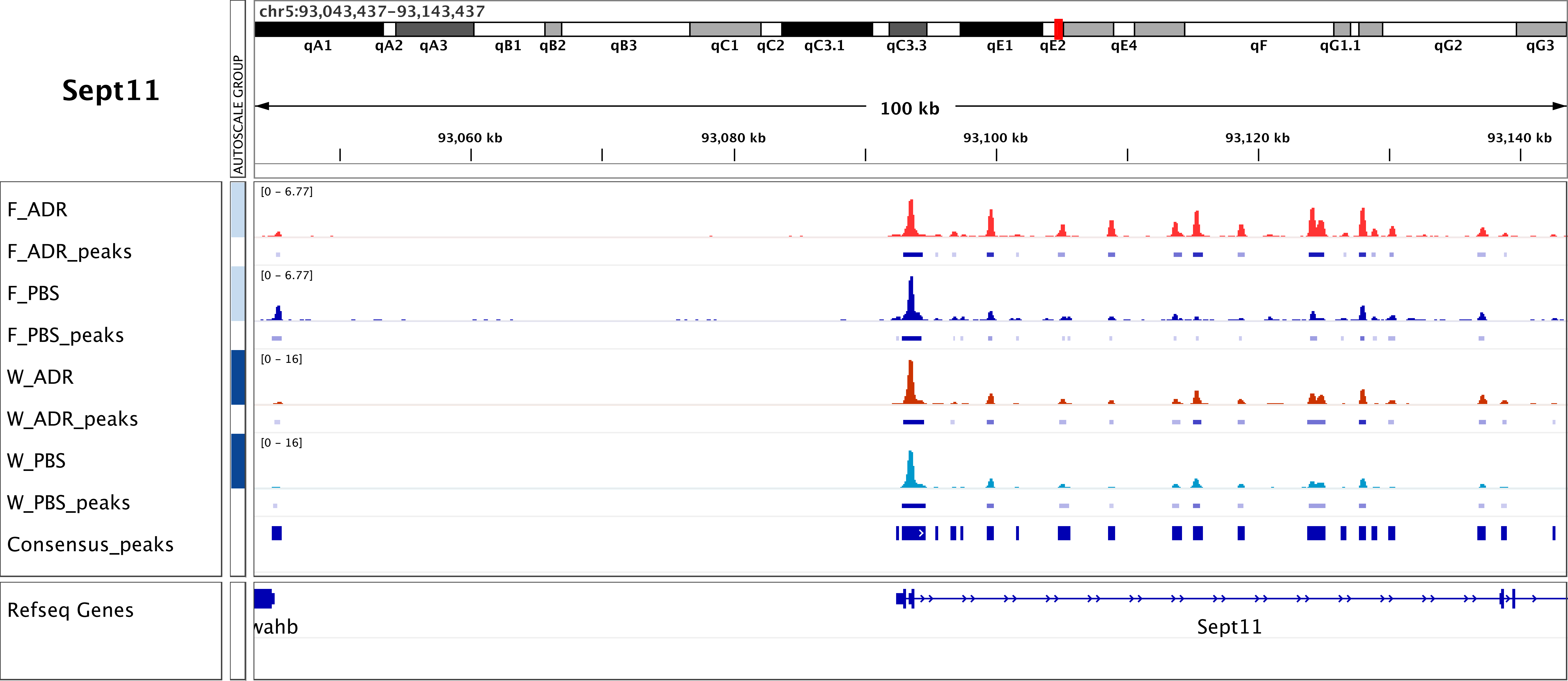

Sept11
AUTOSCALE GROUP
chr5:93,043,437-93,143,437
qA1
qA2
qA3
qB1
qB2
qB3
qC1
qC2
qC3.1
qC3.3
qE1
qE2
qE4
qF
qG1.1
qG2
qG3
100 kb
93,060 kb
93,080 kb
93,100 kb
93,120 kb
93,140 kb
F_ADR
[0 - 6.77]
F_ADR_peaks
F_PBS
[0 - 6.77]
F_PBS_peaks
W_ADR
[0 - 16]
W_ADR_peaks
W_PBS
[0 - 16]
W_PBS_peaks
Consensus_peaks
Refseq Genes
vahb
Sept11

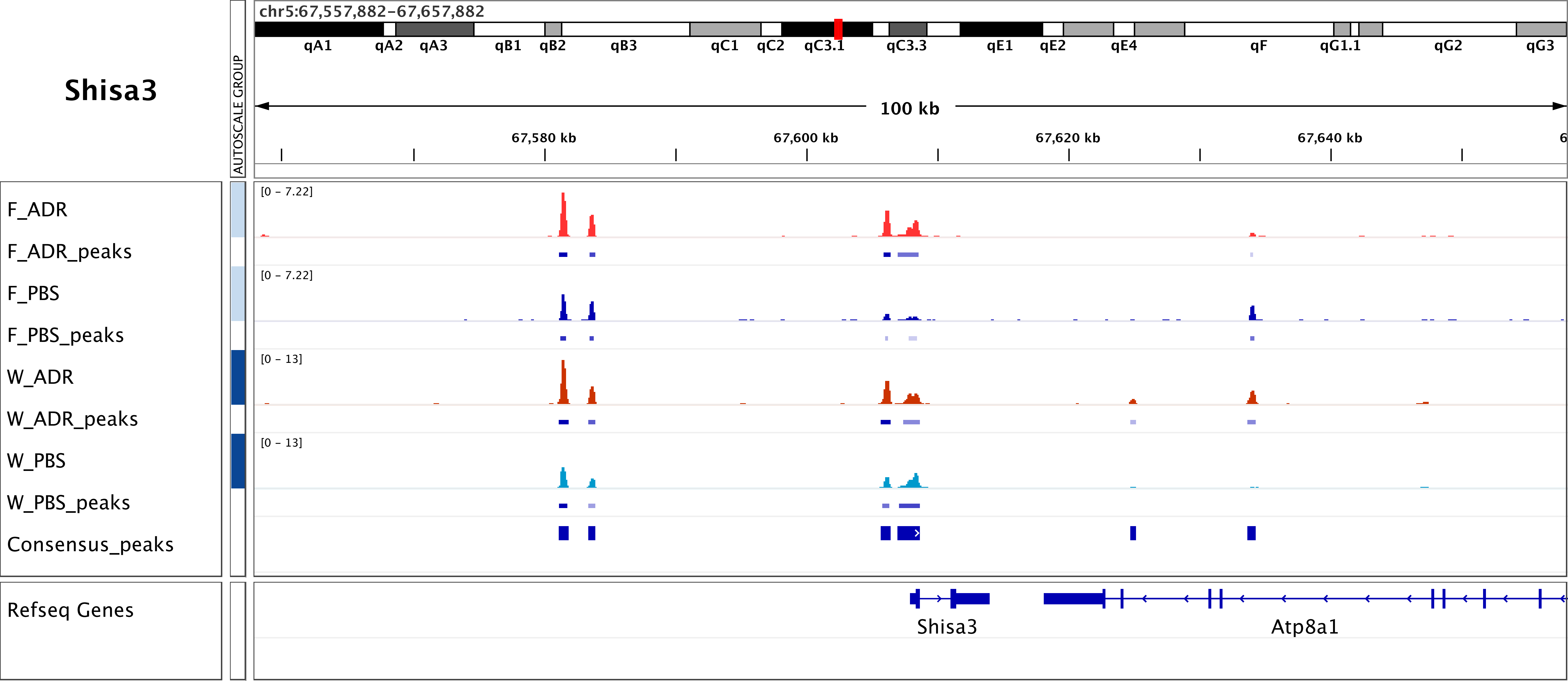

Shisa3
chr5:67,557,882-67,657,882
qA1
qA2
qA3
qB1
qB2
qB3
qC1
qC2
qC3.1
qC3.3
qE1
qE2
qE4
qF
qG1.1
qG2
qG3
AUTOSCALE GROUP
100 kb
67,580 kb
67,600 kb
67,620 kb
67,640 kb
F_ADR
F_ADR_peaks
F_PBS
F_PBS_peaks
W_ADR
W_ADR_peaks
W_PBS
W_PBS_peaks
Consensus_peaks
[0 - 7.22]
[0 - 7.22]
[0 - 13]
[0 - 13]
Refseq Genes
Shisa3
Atp8a1

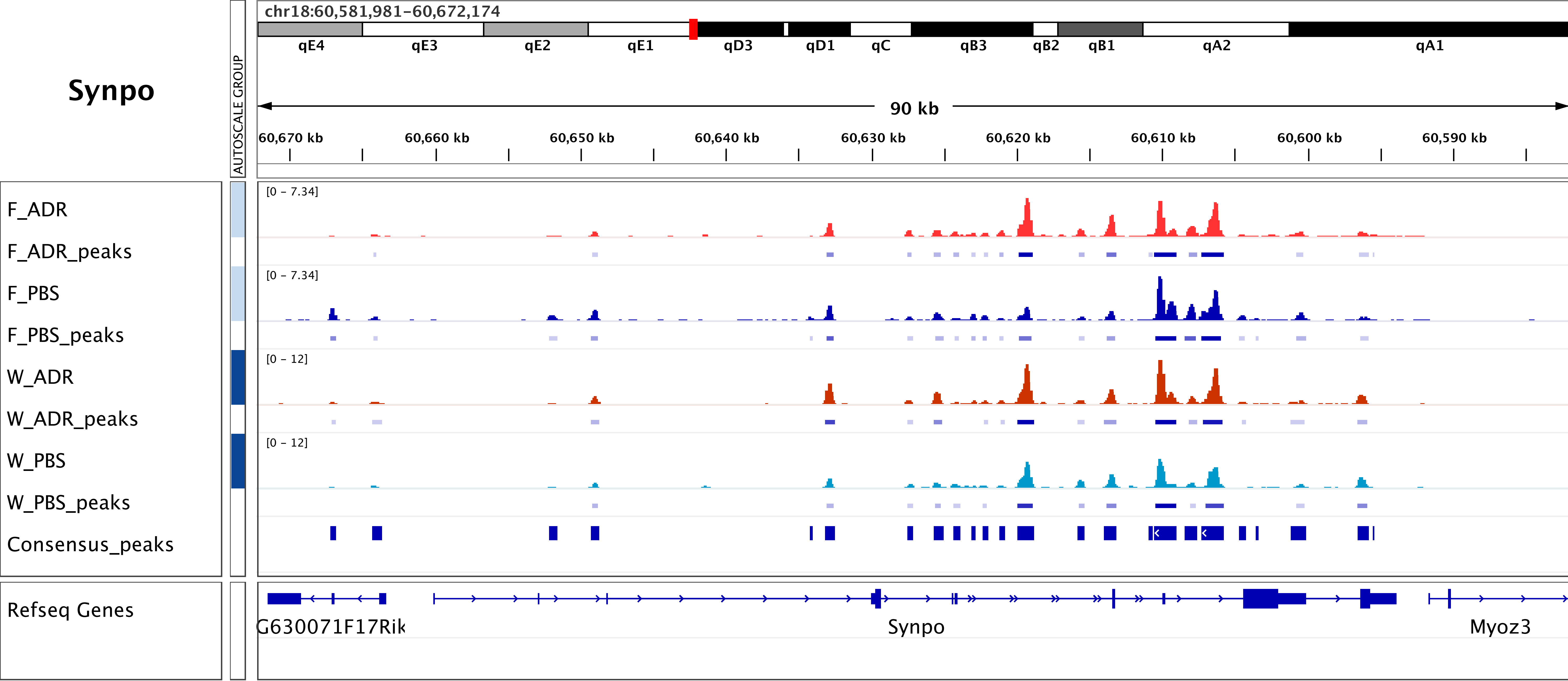
Synpo
AUTOSCALE GROUP
chr18:60,581,981-60,672,174
qE4
qE3
qE2
qE1
qD3
qD1
qC
qB3
qB2
qB1
qA2
qA1
90 kb
60,670 kb
60,660 kb
60,650 kb
60,640 kb
60,630 kb
60,620 kb
60,610 kb
60,600 kb
60,590 kb
F_ADR
[0 - 7.34]
F_ADR_peaks
F_PBS
[0 - 7.34]
F_PBS_peaks
W_ADR
[0 - 12]
W_ADR_peaks
W_PBS
[0 - 12]
W_PBS_peaks
Consensus_peaks
Refseq Genes
G630071F17Rik
Synpo
Myoz3

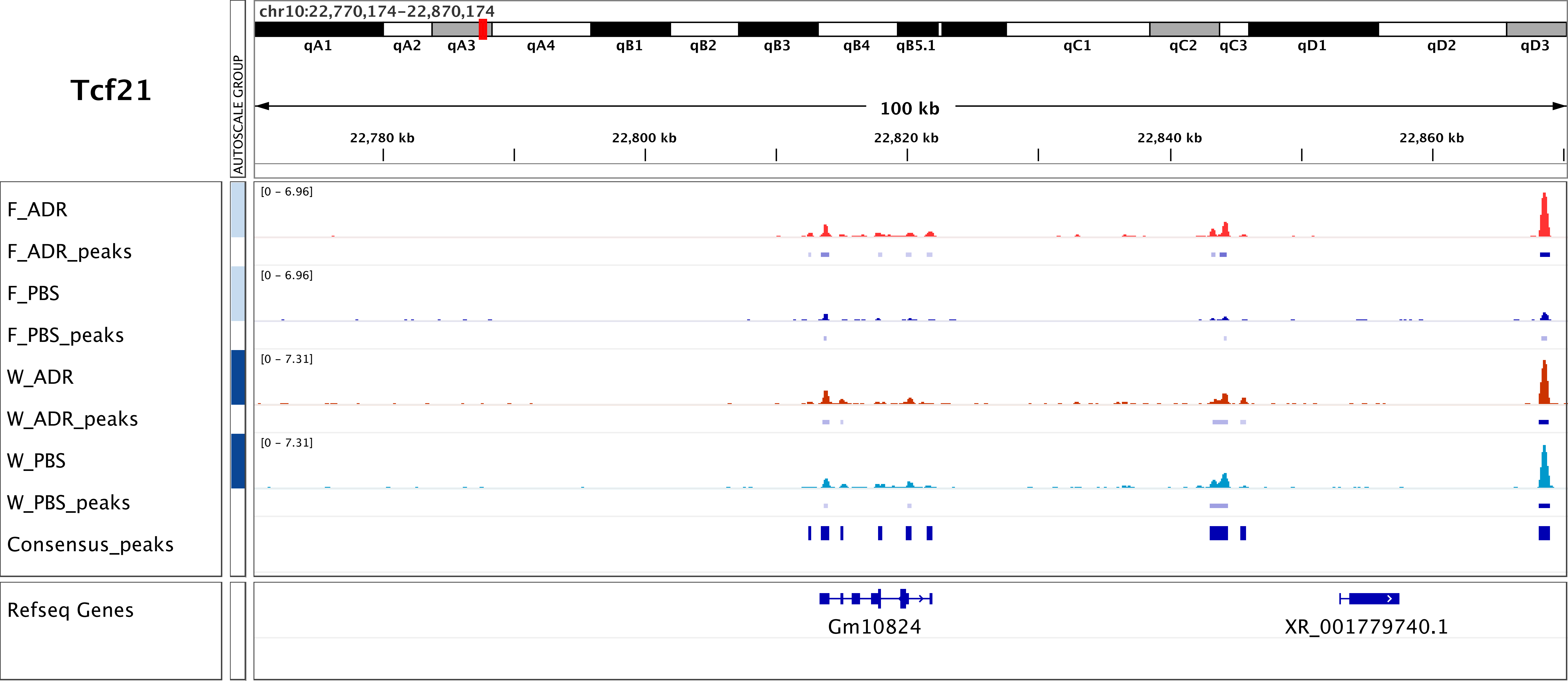

Tcf21
AUTOSCALE GROUP
chr10:22,770,174-22,870,174
qA1
qA2
qA3
qA4
qB1
qB2
qB3
qB4
qB5.1
qC1
qC2
qC3
qD1
qD2
qD3
100 kb
22,780 kb
22,800 kb
22,820 kb
22,840 kb
22,860 kb
F_ADR
[0 - 6.96]
F_ADR_peaks
F_PBS
[0 - 6.96]
F_PBS_peaks
W_ADR
[0 - 7.31]
W_ADR_peaks
W_PBS
[0 - 7.31]
W_PBS_peaks
Consensus_peaks
Refseq Genes
Gm10824
XR_001779740.1

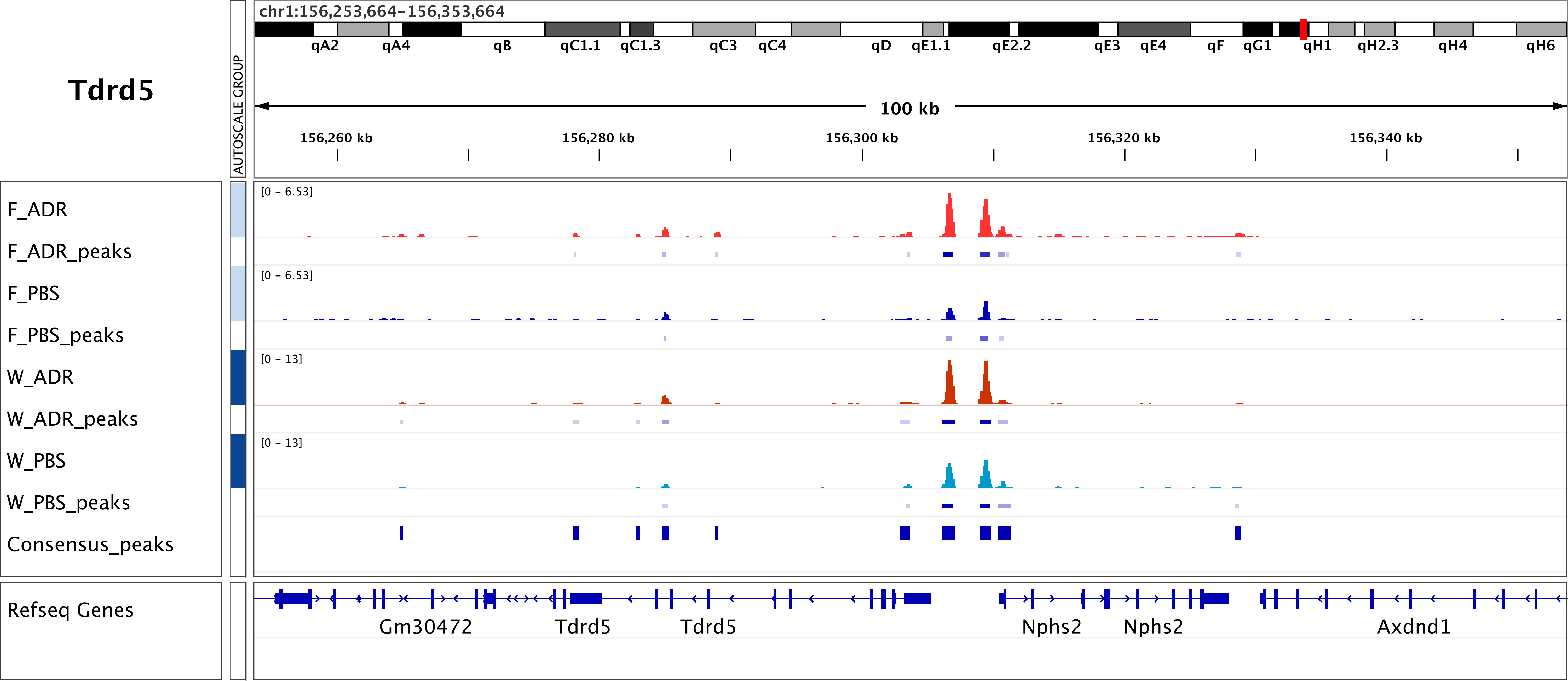
Tdrd5
chr1:156,253,664-156,353,664
qA2
qA4
qB
qC1.1
qC1.3
qC3
qC4
qD
qE1.1
qE2.2
qE3
qE4
qF
qG1
qH1
qH2.3
qH4
qH6
AUTOSCALE GROUP
100 kb
156,260 kb
156,280 kb
156,300 kb
156,320 kb
156,340 kb
F_ADR
[0 - 6.53]
F_ADR_peaks
F_PBS
[0 - 6.53]
F_PBS_peaks
W_ADR
[0 - 13]
W_ADR_peaks
W_PBS
[0 - 13]
W_PBS_peaks
Consensus_peaks
Refseq Genes
Gm30472
Tdrd5
Tdrd5
Nphs2
Nphs2
Axdnd1

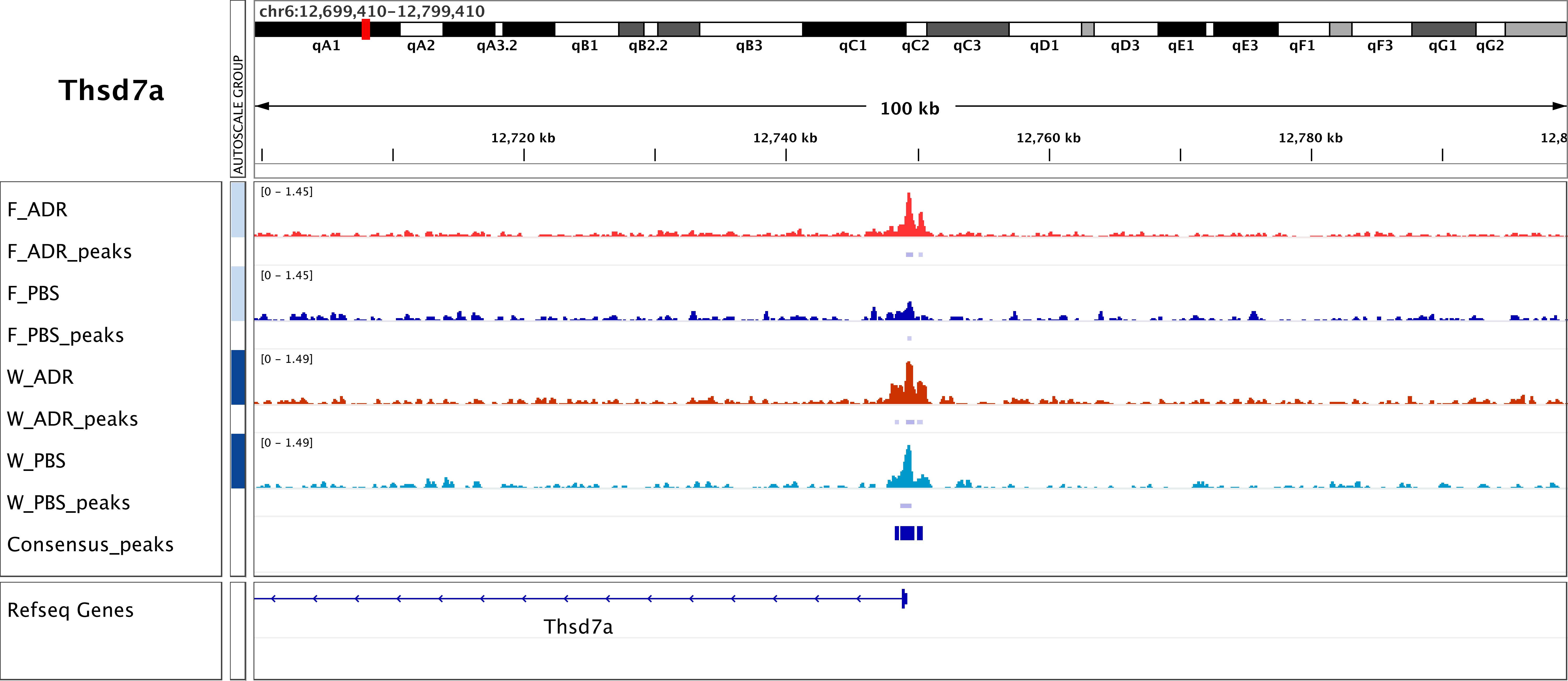

Thsd7a
AUTOSCALE GROUP
chr6:12,699,410-12,799,410
qA1
qA2
qA3.2
qB1
qB2.2
qB3
qC1
qC2
qC3
qD1
qD3
qE1
qE3
qF1
qF3
qG1
qG2
100 kb
12,720 kb
12,740 kb
12,760 kb
12,780 kb
F_ADR
F_ADR_peaks
F_PBS
F_PBS_peaks
W_ADR
W_ADR_peaks
W_PBS
W_PBS_peaks
Consensus_peaks
[0 - 1.45]
[0 - 1.45]
[0 - 1.49]
[0 - 1.49]
Refseq Genes
Thsd7a

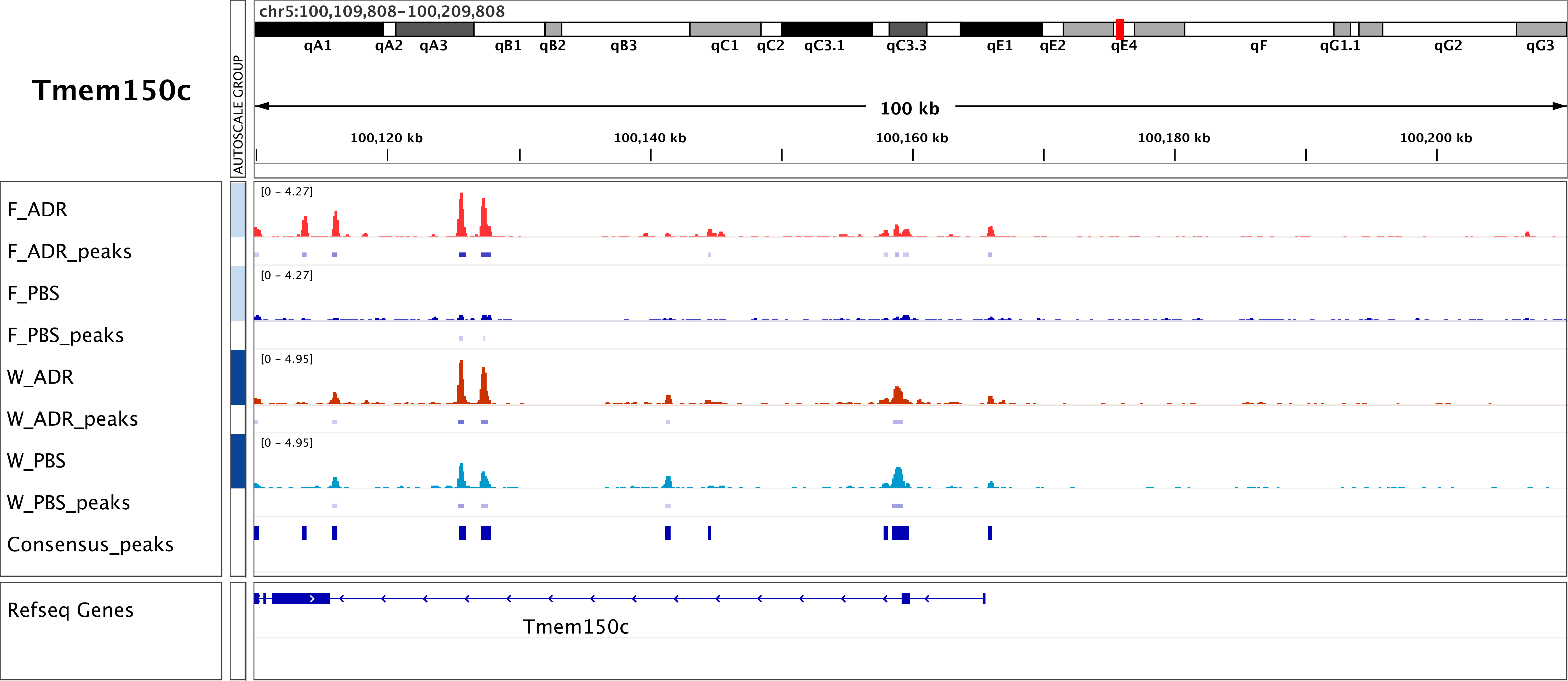

Tmem150c
AUTOSCALE GROUP
chr5:100,109,808-100,209,808
qA1
qA2
qA3
qB1
qB2
qB3
qC1
qC2
qC3.1
qC3.3
qE1
qE2
qE4
qF
qG1.1
qG2
qG3
100 kb
100,120 kb
100,140 kb
100,160 kb
100,180 kb
100,200 kb
F_ADR
F_ADR_peaks
F_PBS
F_PBS_peaks
W_ADR
W_ADR_peaks
W_PBS
W_PBS_peaks
Consensus_peaks
[0 - 4.27]
[0 - 4.27]
[0 - 4.95]
[0 - 4.95]
Refseq Genes
Tmem150c

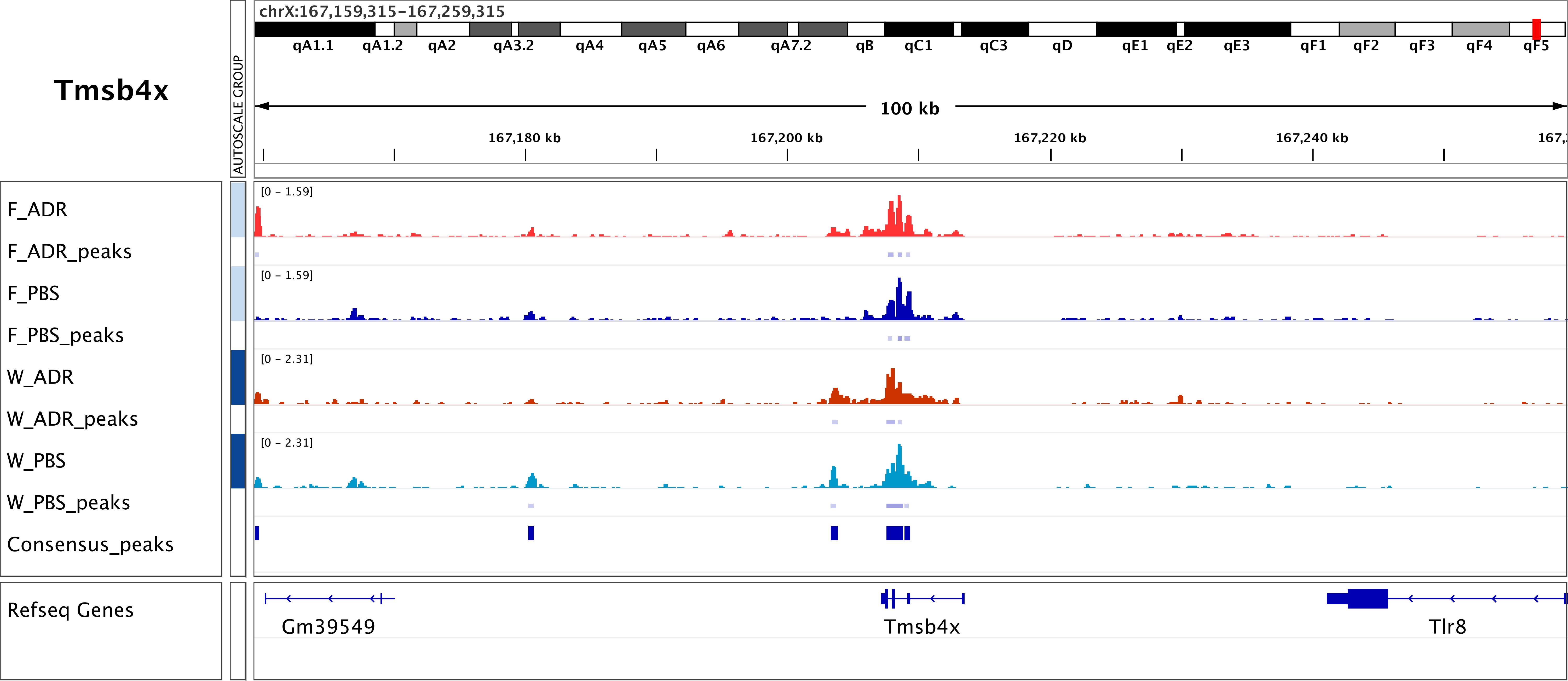
Tmsb4x
AUTOSCALE GROUP
chrX:167,159,315-167,259,315
qA1.1
qA1.2
qA2
qA3.2
qA4
qA5
qA6
qA7.2
qB
qC1
qC3
qD
qE1
qE2
qE3
qF1
qF2
qF3
qF4
qF5
100 kb
167,180 kb
167,200 kb
167,220 kb
167,240 kb
F_ADR
F_ADR_peaks
F_PBS
F_PBS_peaks
W_ADR
W_ADR_peaks
W_PBS
W_PBS_peaks
Consensus_peaks
[0 - 1.59]
[0 - 1.59]
[0 - 2.31]
[0 - 2.31]
Refseq Genes
Gm39549
Tmsb4x
Tlr8

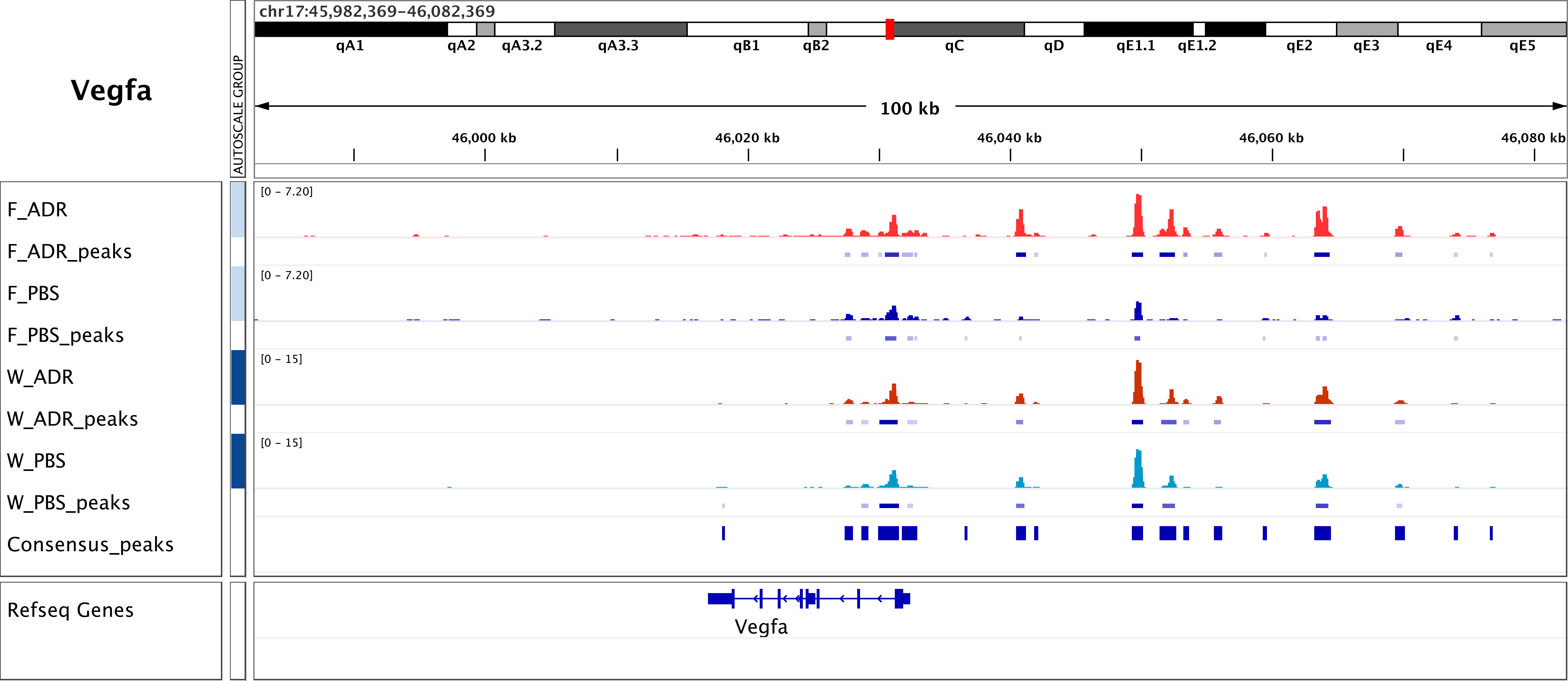

Vegfa
AUTOSCALE GROUP
chr17:45,982,369-46,082,369
qA1
qA2
qA3.2
qA3.3
qB1
qB2
qC
qD
qE1.1
qE1.2
qE2
qE3
qE4
qE5
100 kb
46,000 kb
46,020 kb
46,040 kb
46,060 kb
46,080 kb
F_ADR
[0 - 7.20]
F_ADR_peaks
F_PBS
[0 - 7.20]
F_PBS_peaks
W_ADR
[0 - 15]
W_ADR_peaks
W_PBS
[0 - 15]
W_PBS_peaks
Consensus_peaks
Refseq Genes
Vegfa

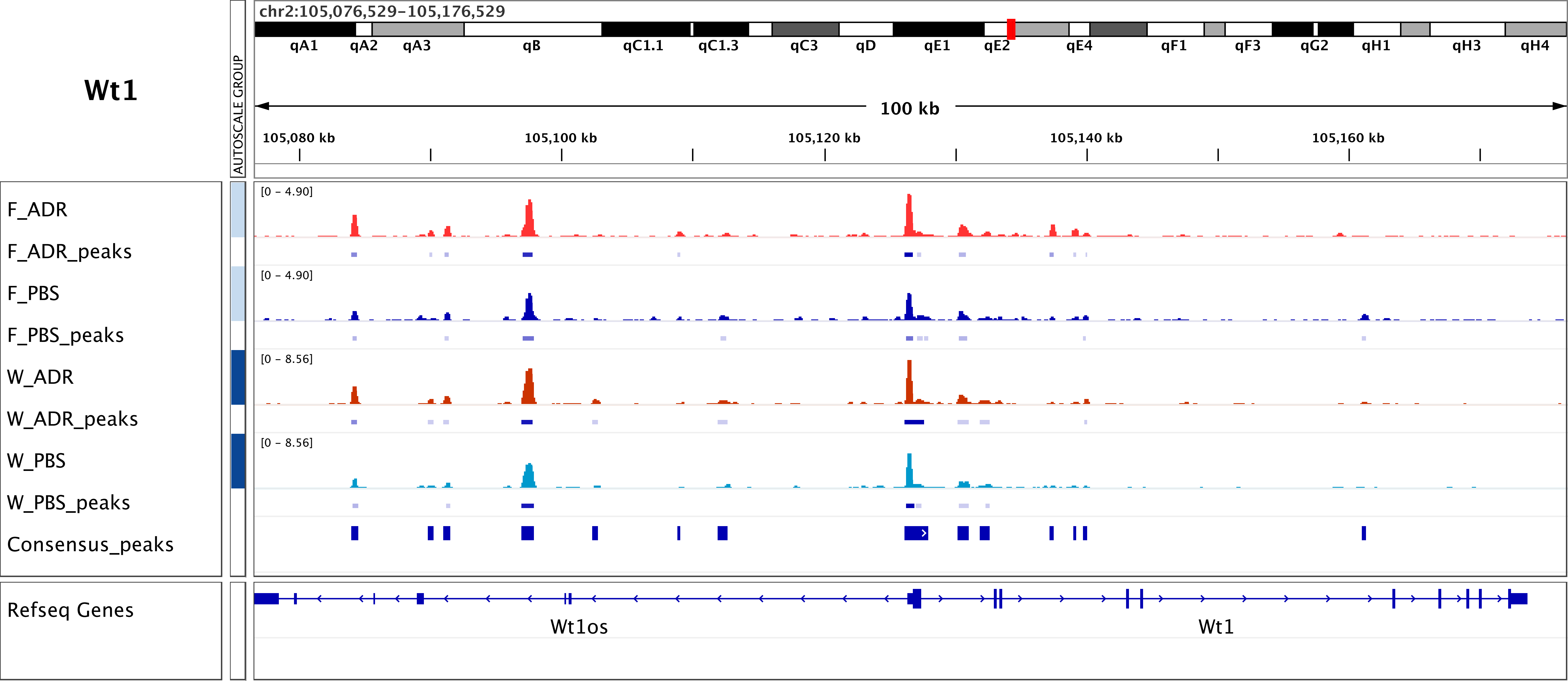

Wt1
AUTOSCALE GROUP
chr2:105,076,529-105,176,529
qA1
qA2
qA3
qB
qC1.1
qC1.3
qC3
qD
qE1
qE2
qE4
qF1
qF3
qG2
qH1
qH3
qH4
100 kb
105,080 kb
105,100 kb
105,120 kb
105,140 kb
105,160 kb
F_ADR
F_ADR_peaks
F_PBS
F_PBS_peaks
W_ADR
W_ADR_peaks
W_PBS
W_PBS_peaks
Consensus_peaks
[0 - 4.90]
[0 - 4.90]
[0 - 8.56]
[0 - 8.56]
Refseq Genes
Wt1os
Wt1

Figure S8

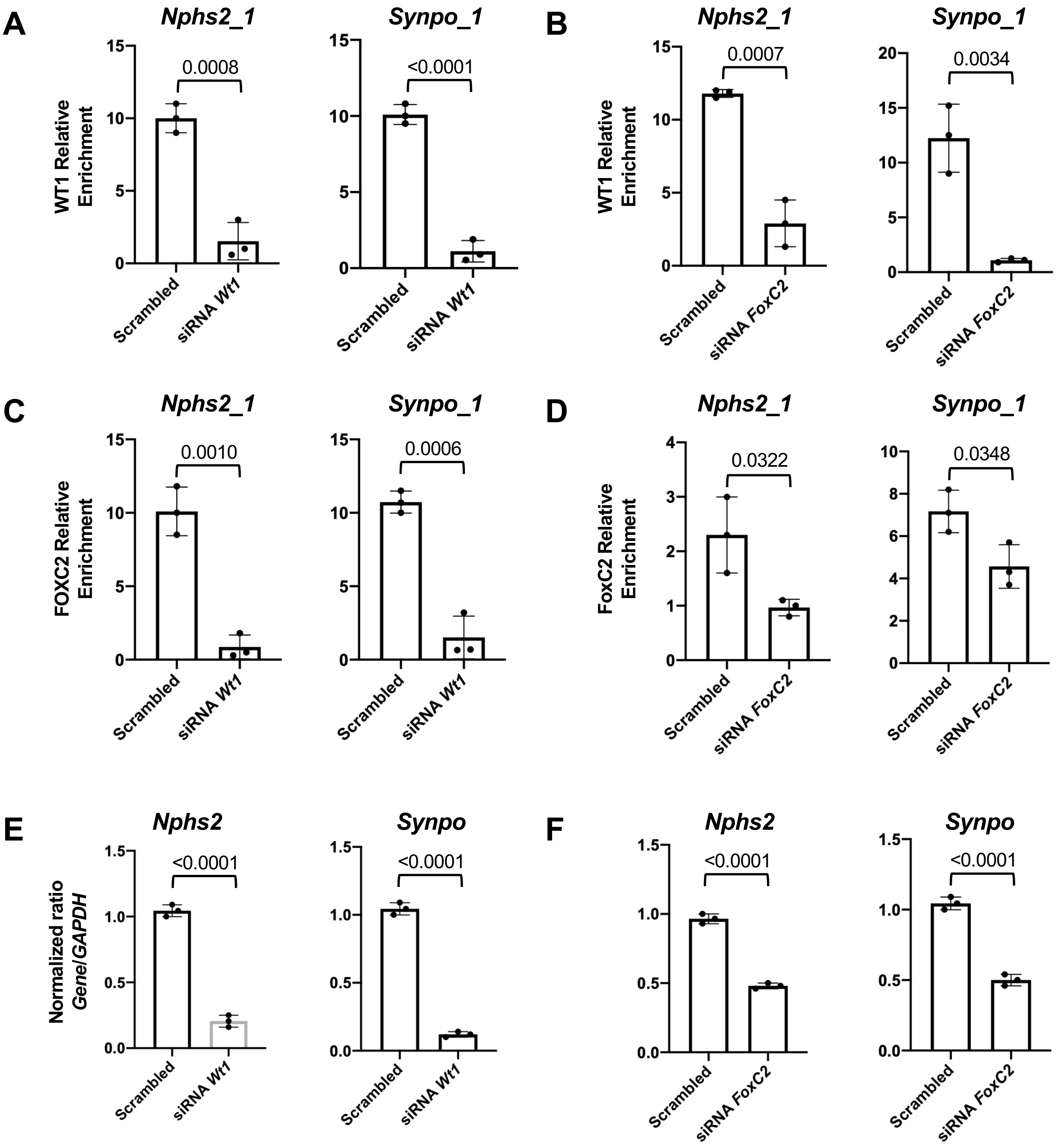

A
Nphs2_1
Synpo_1
WT1 Relative Enrichment
0.0008
<0.0001
Scrambled
siRNA Wt1
B
Nphs2_1
Synpo_1
0.0007
0.0034
siRNA FoxC2
C
FOXC2 Relative Enrichment
0.0010
0.0006
D
FoxC2 Relative Enrichment
0.0322
0.0348
E
Nphs2
Synpo
Normalized ratio Gene/GAPDH
<0.0001
F

**Figure S8. Interdependency of WT1 and FOXC2 binding in immortalized podocytes.** Cells were harvested after siRNA treatment for 72 hours. Each graph includes a scrambled and specific siRNA treatment. Only binding at *Nphs2_1* and *Synpo_1* sites were analyzed. (A) WT1 ChIP-qPCR after *Wt1* siRNA; (B) WT1 ChIP-qPCR after *FoxC2* siRNA. (C) FOXC2 ChIP-qPCR after *Wt1* siRNA; (D) FOXC2 ChIP-qPCR after *FoxC2* siRNA; (E) RT-qPCR expression of *Nphs2* and *Synpo* after treatment with *Wt1* siRNA. (F) same as (E) except *FoxC2* siRNA. Data are presented as mean ± SD. Two-tailed unpaired t-test.

# Figure S9

A

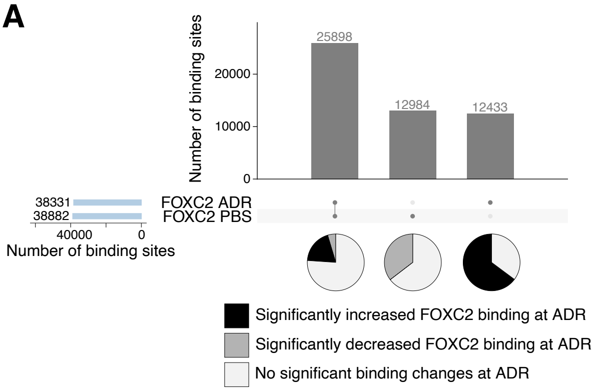


B

**FOXC2 bound in PBS**

| TF Family | Top TF | Top Motif | Adj. P |
|---|---|---|---|
| Three-zinc finger Kruppel-related (17) | KLF15 | | 1.74e-332 |
| ZBTB7A (1) | ZBTB7A | | 2.24e-271 |
| Ets-related (7) | ELK4 | | 1.90e-266 |
| E2F (4) | TFDP1 | | 1.09e-255 |
| ZBTB14 (1) | ZBTB14 | | 2.34e-243 |
| Fos-related (6) | FOSL2 | | 7.76e-220 |
| Hairy-related factors (3) | HES1 | | 5.72e-125 |
| Three-zinc finger Kruppel-related (17) | KLF6 | | 1.73e-109 |
| HINFP (1) | HINFP | | 5.89e-107 |
| MAZ (1) | MAZ | | 3.64e-104 |
| CTCF (3) | CTCF | | 1.09e-54 |
| Plagl1 (1) | PLAGL1 | | 1.22e-50 |
| CREB-related factors (4) | ATF1 | | 1.38e-47 |
| PAS domain factors (2) | HIF1A | | 2.99e-28 |
| Heteromeric CCAAT-binding (2) | NFYB | | 4.19e-22 |
| NF-kappaB-related factors (2) | RELA | | 6.29e-22 |
| VEZF1 (1) | VEZF1 | | 1.82e-17 |
| bHLH-ZIP (6) | MLX | | 6.38e-17 |
| THAP-related factors (1) | THAP11 | | 1.11e-15 |
| Maf-related (1) | MAFK | | 1.22e-13 |
| RFX-related factors (3) | RFX2 | | 1.22e-09 |
| Wt1 (1) | WT1 | | 7.60e-09 |
| STAT factors (2) | STAT1 | | 2.18e-08 |
| ZBTB7B (1) | ZBTB7B | | 1.50e-07 |
| XBP-1-related factors (1) | XBP1 | | 1.05e-06 |

**FOXC2 bound in ADR**

| TF Family | Top TF | Top Motif | Adj. P |
|---|---|---|---|
| Wt1 (1) | WT1 | | 1.74e-332 |
| Three-zinc finger Kruppel-related (17) | EGR1 | | 2.80e-184 |
| ZBTB14 (1) | ZBTB14 | | 4.52e-132 |
| E2F (2) | TFDP1 | | 1.12e-91 |
| Jun-related (1) | NRF1 | | 4.17e-74 |
| Three-zinc finger Kruppel-related (17) | KLF6 | | 1.73e-58 |
| Paired-related HD factors (1) | ESX1 | | 2.25e-56 |
| FOX (11) | FOXC2 | | 6.18e-49 |
| HD-LIM (1) | LMX1B | | 9.79e-46 |
| MAZ (1) | MAZ | | 1.52e-44 |
| HOX (10) | HOXA7 | | 1.75e-43 |
| HINFP (1) | HINFP | | 1.78e-42 |
| Hairy-related factors (1) | HES1 | | 4.00e-42 |
| ZFP14 (1) | ZFP14 | | 1.81e-14 |
| TEF-1-related factors (1) | TEAD3 | | 1.92e-13 |
| THAP-related factors (1) | THAP11 | | 4.23e-13 |
| Ets-related (2) | ELK4 | | 4.95e-11 |
| Tal-related (1) | TCF21 | | 7.05e-10 |
| Heteromeric CCAAT-binding (2) | NFYC | | 8.47e-10 |
| CEBP-related (2) | DBP | | 3.77e-09 |
| bHLH-ZIP (1) | USF2 | | 9.25e-08 |
| PAS domain factors (1) | ARNT | | 2.11e-07 |
| ZBTB7A (1) | ZBTB7A | | 4.66e-07 |

**Figure S9. Analyses of FOXC2 ChIP-seq.** (A) Upper: Upset plot of FOXC2 binding sites in ADR, PBS or both as indicated below bars; Lower: Pie charts show proportion of sites for each bar that have significantly increased, decreased, or no binding changes in ADR as indicated by boxes below charts. (B) Motifs enriched near sites where FOXC2 was bound in PBS (left) and ADR (right) conditions.

# Figure S10

**A**

**Increased binding at D9**

| TF Family | Top TF | Top Motif | Adj. P |
|---|---|---|---|
| FOX (11) | FOXC2 | | 2.66e-380 |
| HOX (13) | HOXA7 | | 2.14e-254 |
| Paired-related HD factors (1) | ESX1 | | 6.58e-183 |
| HD-LIM (1) | LMX1B | | 2.14e-151 |
| POU domain factors (1) | POU3F1 | | 2.8e-104 |
| Regulators of differentiation (1) | MEF2A | | 2.12e-102 |
| SOX-related factors (5) | SOX8 | | 1.62e-95 |
| PBX2 (1) | PBX2 | | 4.19e-95 |
| Maf-related (4) | MAFF | | 1.97e-94 |
| Maf-related (4) | Mafb | | 9.37e-60 |
| TEF-1-related factors (1) | TEAD3 | | 3.79e-94 |
| ARID-related (1) | ARID5A | | 3.21e-82 |
| STAT factors (3) | STAT2 | | 1.74e-67 |
| TBP-related factors (1) | TBP | | 3.22e-58 |
| CEBP-related (5) | NFIL3 | | 1.79e-46 |
| LIN54 (1) | LIN54 | | 2.37e-39 |
| Interferon-regulatory factors (1) | IRF1 | | 6.57e-38 |
| NR (4) | VDR | | 4.04e-35 |
| Tal-related (2) | TCF21 | | 2.1e-28 |
| CSL-related factors (1) | RBPJL | | 1.65e-27 |
| MEIS3 (1) | MEIS3 | | 2.37e-20 |
| Factors with multiple dispersed zinc fingers (1) | ZFP335 | | 9.48e-10 |

**Decreased binding at D9**

| TF Family | Top TF | Top Motif | Adj. P |
|---|---|---|---|
| Fos-related (4) | ATF3 | | 1.69e-332 |
| Maf-related (4) | MAFK | | 1.64e-145 |
| Maf-related (4) | Mafb | | 5.72e-06 |
| Ets-related (2) | GABPA | | 7.21e-34 |
| CEBP-related (5) | CEBPG | | 7.88e-33 |
| FOX (9) | FOXO1 | | 3.05e-26 |
| FOX (9) | FOXC2 | | 6.96e-15 |
| Regulators of differentiation (3) | MEF2A | | 2.98e-24 |
| NR (8) | NR4A1 | | 1.22e-22 |
| PBX2 (1) | PBX2 | | 7.55e-17 |
| ATF-4-related factors (1) | ATF4 | | 3.93e-16 |
| STAT factors (6) | STAT5A | | 1.81e-14 |
| HOX (9) | HOXA7 | | 8.43e-14 |
| Factors with multiple dispersed zinc fingers (1) | ZKSCAN5 | | 4.83e-12 |
| ARID-related (1) | ARID5A | | 4.33e-10 |
| POU domain factors (1) | POU3F1 | | 4.61e-09 |
| MEIS3 (1) | MEIS3 | | 1.22e-08 |
| SOX-related factors (2) | SOX8 | | 5.1e-08 |
| Tal-related (1) | TCF21 | | 6.37e-08 |
| TEF-1-related factors (1) | TEAD3 | | 4.98e-06 |

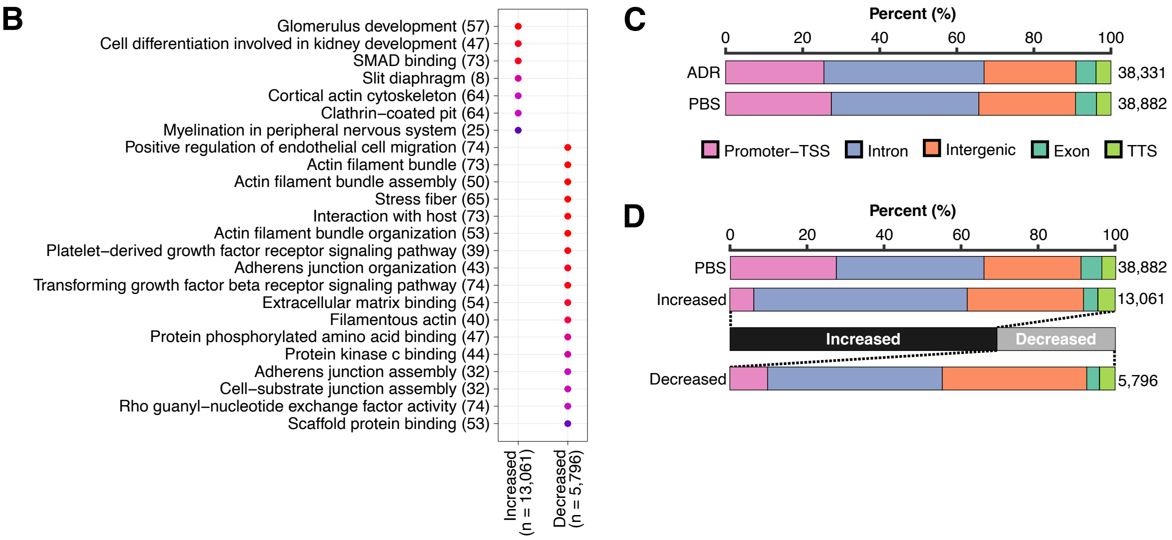

**Figure S10. Analysis of FOXC2 differential binding.** (A) Left: TF motifs enriched near sites where FOXC2 peak intensity significantly increased in ADR; Right: motifs enriched near sites where binding significantly decreased in ADR. (B) GO analysis based on peaks bound where FOXC2 binding increased (left column) or decreased (right column) in ADR. (C) Bar plot shows proportion of FOXC2 bound sites genome-wide by sequence type. (D) Genomic distribution stratified by increased and decreased FOXC2 binding in ADR (lower) compared to PBS (top).

## Figure S11

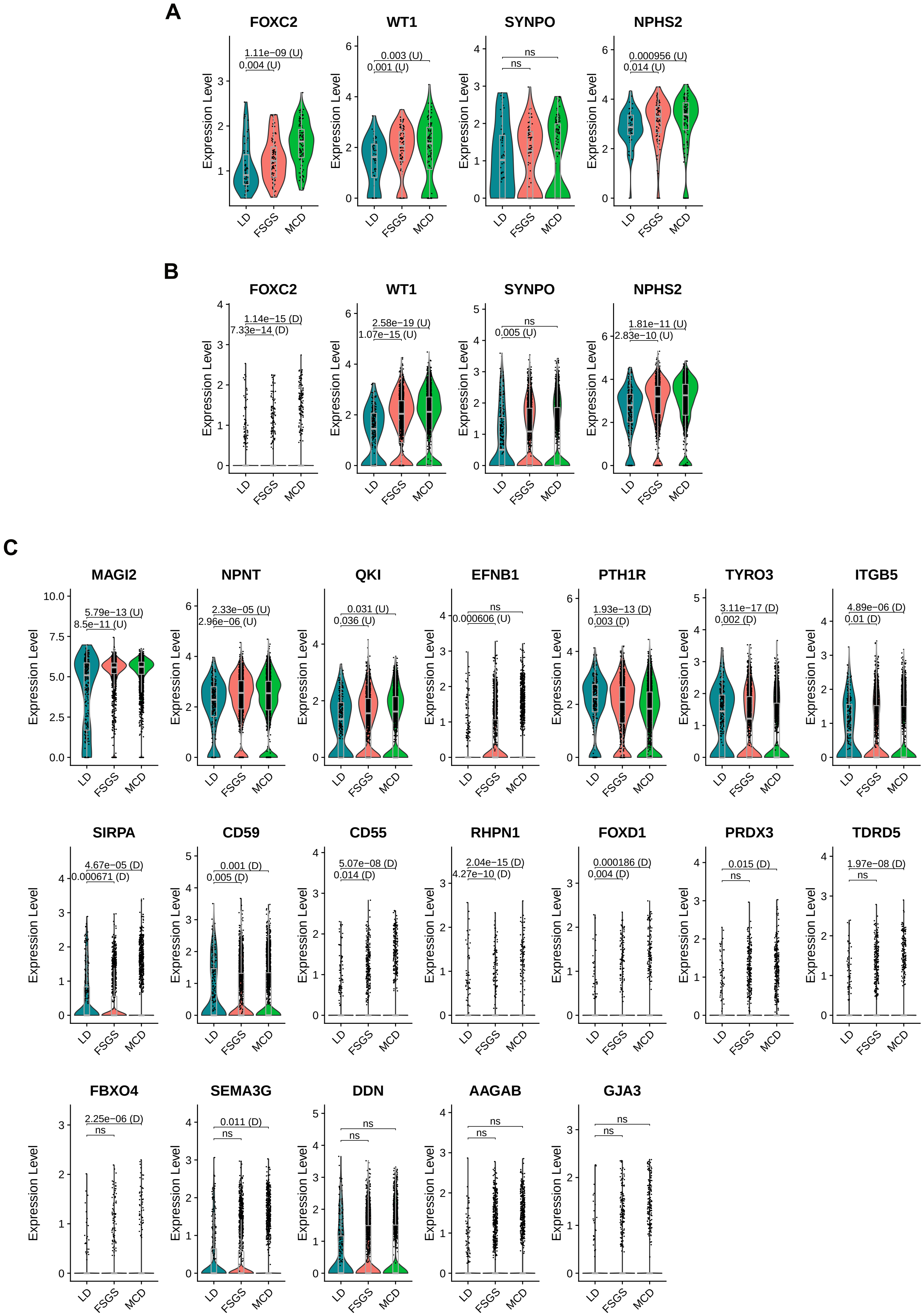
A
FOXC2
1.11e−09 (U)
0.004 (U)
WT1
0.003 (U)
0.001 (U)
SYNPO
ns
ns
NPHS2
0.000956 (U)
0.014 (U)
Expression Level
LD
FSGS
MCD
B
FOXC2
1.14e−15 (D)
7.33e−14 (D)
WT1
2.58e−19 (U)
1.07e−15 (U)
SYNPO
ns
0.005 (U)
NPHS2
1.81e−11 (U)
2.83e−10 (U)
C
MAGI2
5.79e−13 (U)
8.5e−11 (U)
NPNT
2.33e−05 (U)
2.96e−06 (U)
QKI
0.031 (U)
0.036 (U)
EFNB1
ns
0.000606 (U)
PTH1R
1.93e−13 (D)
0.003 (D)
TYRO3
3.11e−17 (D)
0.002 (D)
ITGB5
4.89e−06 (D)
0.01 (D)
SIRPA
4.67e−05 (D)
0.000671 (D)
CD59
0.001 (D)
0.005 (D)
CD55
5.07e−08 (D)
0.014 (D)
RHPN1
2.04e−15 (D)
4.27e−10 (D)
FOXD1
0.000186 (D)
0.004 (D)
PRDX3
0.015 (D)
ns
TDRD5
1.97e−08 (D)
ns
FBXO4
2.25e−06 (D)
ns
SEMA3G
0.011 (D)
ns
DDN
ns
ns
AAGAB
ns
ns
GJA3
ns
ns

**Figure S11. Differential expression of FOXC2 and its target genes in human podocytes.** (A) Violin plots show the expression of *FOXC2* and its known target genes (*WT1*, *SYNPO*, *NPHS2*) in the *FOXC2*-positive podocytes, stratified by the three different groups: Living Donor (LD), FSGS, and MCD. (B) Violin plots for the same genes across all podocytes, including *FOXC2*-negative cells. (C) Violin plots of 11 novel potential *FOXC2* target genes. Significance was determined using a two-sided Wilcoxon rank sum test. (U) denotes upregulation and (D) denotes downregulation relative to the LD reference. P values are in the Figure. Expression levels were calculated using the Seurat LogNormalize function with default parameters. Values represent log-normalized counts, defined as: Ln[(X/N *10,000)+1], where X is the raw counts for a given gene, and N is the total library size (total counts) for that cell. 1 is a pseudocount added to facilitate log transformation of zero values.
