# Supplementary material for: FOXC2 and WT1 regulate transcriptional reprogramming during the podocyte response to injury
Source: JCI Insight. 2026 Jun 8;11(11):e190175. doi: 10.1172/jci.insight.190175 (PMC13313500; doi:10.1172/jci.insight.190175)
Supplement: Unedited blot and gel images [file jciinsight-11-190175-s317.pdf]

The only gel is in Figure 2 and the entire gel is in the figure.
